# A Multiresonant Thermally Activated Delayed Fluorescent Dendrimer with Intramolecular Energy Transfer: Application for Efficient Host‐Free Green Solution‐Processed Organic Light‐emitting Diodes

**DOI:** 10.1002/adma.202415289

**Published:** 2025-01-09

**Authors:** Sen Wu, Dongyang Chen, Xiao‐Hong Zhang, Dianming Sun, Eli Zysman‐Colman

**Affiliations:** ^1^ Organic Semiconductor Centre EaStCHEM School of Chemistry University of St Andrews St Andrews Fife KY16 9ST UK; ^2^ Institute of Functional Nano & Soft Materials (FUNSOM) Joint International Research Laboratory of Carbon‐Based Functional Materials and Devices Soochow University Suzhou Jiangsu 215123 P. R. China; ^3^ Jiangsu Key Laboratory of Advanced Negative Carbon Technologies Soochow University Suzhou Jiangsu 215123 P. R. China

**Keywords:** dendrimer, energy transfer, multiresonant thermally activated delayed fluorescence, solution‐processed organic light‐emitting diodes

## Abstract

The development of narrowband emissive, bright, and stable solution‐processed organic light‐emitting diodes (SP‐OLEDs) remains a challenge. Here, a strategy is presented that merges within a single emitter a TADF sensitizer responsible for exciton harvesting and an MR‐TADF motif that provides bright and narrowband emission. This emitter design also shows strong resistance to aggregate formation and aggregation‐cause quenching. It is based on a known MR‐TADF emitter **DtBuCzB** with a donor‐acceptor TADF moiety consisting of either *tert*‐butylcarbazole donors (**tBuCzCO_2_HDCzB**) or second‐generation carbazole‐based donor dendrons (**2GtBuCzCO_2_HDCzB**) and a benzoate acceptor. The TADF moiety acts as an exciton harvesting antenna and transfers these excitons via Förster resonance energy transfer to the MR‐TADF emissive core. The SP‐OLEDs with **2GtBuCzCO_2_HDCzB** and **tBuCzCO_2_HDCzB** thus show very high maximum external quantum efficiencies (EQE_max_ of 27.9 and 22.0%) and minimal efficiency roll‐off out to 5000 cd m^−2^.

## Graphical abstract

An MR‐TADF dendrimer, **2GtBuCzCO_2_HDCzB**, features an encapsulated MR‐TADF core surrounded by a donor‐acceptor TADF moiety containing donor dendrons. The non‐doped SP‐OLEDs with **2GtBuCzCO_2_HDCzB** exhibit narrowband green emission peaking at 495 nm (FWHM of 30 nm) and show an EQE_max_ at 24.0% and small efficiency roll‐off, with EQE_1000_ of 20.2%.

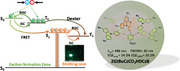

## 1 Introduction

Solution‐processed organic light‐emitting diodes (SP‐OLEDs) offer a cost‐effective and attractive alternative vacuum‐deposited devices (VD‐OLEDs);^[^ ^1^, ^2^, ^3^, ^4^ ^]^ however, historically, the performance of SP‐OLEDs has lagged behind that of VD‐OLEDs, which has impeded their adoption. To achieve comparably high external quantum efficiencies (EQE), judicious materials and device designs are required for SP‐OLEDs.^[^ ^5^, ^6^ ^]^ One design approach involves the utilization of thermally activated delayed fluorescence (TADF) emitters as materials harvest both singlet and triplet excitons to produce light owing to their small energy gap between the singlet and triplet excited states (Δ*E*_ST_) that enable the upconversion of triplet excitons to emissive singlets via reverse intersystem crossing (RISC).^[^ ^7^, ^8^, ^9^, ^10^ ^]^ Therefore, like phosphorescent OLEDs, TADF‐OLEDs can achieve 100% internal quantum efficiency.^[^ ^11^, ^12^, ^13^ ^]^

Multiresonant TADF (MR‐TADF) emitters, first reported by Hatakeyama et al., are narrowband emissive compounds, and so are particularly attractive for display applications.^[^ ^14^, ^15^ ^]^ Their rigid structure and the short‐range charge transfer (SRCT) character of their emissive S_1_ state are the origin for the narrowband emission and the moderately small Δ*E*_ST_ that enables TADF. However, their typically planar structure also makes these materials prone to aggregation‐cased quenching (ACQ). Thus, MR‐TADF OLEDs typically use the emitter at very low doping concentrations, which results in a narrow recombination zone that contributes to the severe efficiency roll‐off in the devices. The incorporation of bulky substituents on the MR‐TADF emitters has been demonstrated to be an effective strategy to mitigate against aggregation. Zhang et al.^[^ ^16^ ^]^ encapsulated the MR‐TADF core, **DtBuCzB**, with an *ortho,ortho*‐diDtCzPh group within **D‐Cz‐BN** and an *ortho*‐CzPh group in **S‐Cz‐BN** as a comparison (Figure S1, Supporting Information) as a strategy to mitigate aggregation. **D‐Cz‐BN** shows improved resistance to ACQ as the Φ_PL_ decreased modestly from 98 to 90% as the doping concentration increased from 1 to 30 wt% in 3,3‐bis(*N*‐carbazolyl)‐1,1′‐biphenyl compared to that of **S‐Cz‐BN** (95 to 84%) and the naked MR‐TADF core, **DtBuCzB**, (92 to 48%). The EQE_max_ of the OLEDs with **D‐Cz‐BN** exhibited less sensitivity to the doping concentration of the emitter, decreasing from 28.7 to 24.8% in comparison the devices with **S‐Cz‐BN** and **DtBuCzB** where the EQE_max_ decreased from 22.1 to 16.1% and 21.0 to 9.9%, respectively, as the doping concentration increased from 5 to 20 wt%. However, all devices (5 wt% emitter doping) showed significant efficiency roll‐off, with EQE_1000_ of 12.4, 11.4, and 11.7%, respectively. The device performance was significantly improved with the addition of **CTPCF3** as an assistant dopant in HF‐OLEDs, which showed EQE_max_ of 30.5, 37.2, and 27.5%, respectively, while the EQE_1000_ remained high at 26.2, 34.3 and 24.1%, respectively. Using a very similar emitter design, Jiang et al. reported **BN‐CP1** and **BN‐CP2**, two compounds featuring carbazoles positioned either at the *ortho* or *meta* positions of a pendant phenyl ring, which itself is substituted *para* to the boron atom of the MR‐TADF core **DtBuCzB** (Figure S1, Supporting Information).^[^ ^17^ ^]^ **BN‐CP1,** a carbazole analog to **D‐Cz‐BN**, is more resistant to ACQ, evidenced by the almost unchanged FWHM of 25 to 26 nm upon increasing the doping concentration from 1 to 30 wt% in DMIC‐TRZ film, while the FWHM broadens from 26 to 43 nm for **BN‐CP2** across the same range of doping concentrations. The device with **BN‐CP1** showed an unchanged electroluminescence (EL), λ_EL_ = 496 nm and FWHM = 25 nm, and a smaller decrease in the EQE_max_ from 40.0 to 33.3%, as the doping concentration increased from 5 to 30 wt%. By contrast, the device with **BN‐CP2** showed red‐shifted and broadened EL (λ_EL_ from 494 to 502 nm and FWHM from 25 to 33 nm) and the EQE_max_ decreased to 23.7 from 36.7% across the same doping concentration range. These two studies reveal the value of judiciously placed bulky substituents to mitigate ACQ. Following a similar strategy, Xie et al.^[^ ^18^ ^]^ reported another analog of **D‐Cz‐BN** that contains four *tert*‐butyl carbazoles on the pendant phenyl ring of the same **DtBuCzB** MR‐TADF core (**6TBN**, Figure S1, Supporting Information). **6TBN** also exhibited strong resistance to aggregation, reflecting in its almost unchanged emission spectra (λ_PL_ ranging from 497–499 nm and FWHM ranging from 25–29 nm) in mCP films at doping concentrations ranging from 10 to 100 wt%. The 20 wt% in mCP and host‐free SP‐OLEDs with **6TBN** showed an EQE_max_ of 23.0 and 12.3%, respectively. Luo et al. reported an encapsulated TADF emitter, **NBNN2** (Figure S1, Supporting Information)^[^ ^19^ ^]^ that contains the **BN2** MR‐TADF core, which is flanked by N‐centred and O‐bridging donor (DPXZ) moieties, all linked via a common carbazole scaffold. This sandwich configuration endows **NBNN2** a resistance to aggregation as the emission profile is almost unchanged (λ_PL_ ranging from 531 to 534 nm and FWHM ranging from 39 to 40 nm) as the doping concentration increased from 10 to 50 wt% in mCP; however, there is a rather significant decrease in the Φ_PL_ from 91 to 52%. Owing to the through‐space charge transfer excited states that form as a result of the interactions between **BN2** and the flanking groups the reverse intersystem crossing rate constant (*k*_RISC_) was found to be high for MR‐TADF emitters at 1.2×10^5^ s^−1^ in 10 wt% doped films in mCP. The devices with **NBNN2** showed a high EQE_max_ of 31.7% and a small efficiency roll‐off, with EQE_1000_ of 20.9% and EQE_3000_ of 16.7%.

Although the strategy of decorating MR‐TADF cores with bulky groups can effectively suppress the ACQ, the slow RISC rate (*k*_RISC_) of most MR‐TADF results in a build‐up of triplet excitons at high current density, leading ultimately to severe efficiency roll‐off due to exciton annihilation processes such as triplet‐triplet annihilation and triplet‐polaron annihilation (TPA).^[^ ^20^, ^21^, ^22^ ^]^ The most successful solution to address efficiency roll‐off to date has been to decouple exciton harvesting and emission within an HF‐OLED wherein the assistant dopant harvests the excitons before transferring their energy to the terminal MR‐TADF emitter by FRET.^[^ ^23^, ^24^, ^25^ ^]^ To date, there are only a few examples of solution‐processed MR‐TADF OLEDs and those documenting HF‐OLEDs are even more limited.^[^ ^26^, ^27^, ^28^, ^29^, ^30^, ^31^, ^32^ ^]^ This is likely due to the general poor solubility of the MR‐TADF emitter, and for HF‐OLEDs, the difficulty in obtaining homogenous films with high FRET efficiency in ternary solution‐processed blends.^[^ ^33^, ^34^ ^]^

**Figure 1:**
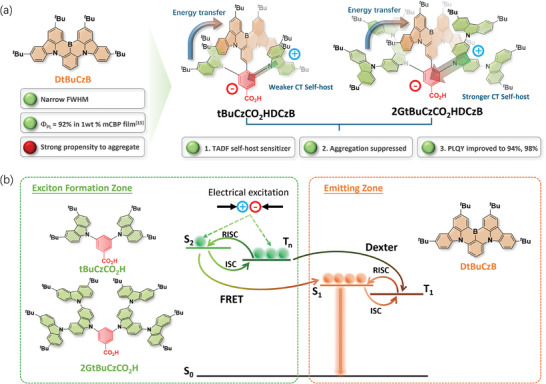
a) The chemical structures of **2GtBuCzCO_2_HDCzB** and **tBuCzCO_2_HDCzB**, and b) the design concept of the intermolecular energy transfer strategy between the peripheral pendant, **tBuCzCO_2_H** and **2GBuCzCO_2_H**, as well as the central MR‐TADF core **DtBuCzB**.

In this work, we propose an emitter design that combines within a single emitter structure a moiety that acts as a TADF sensitizer to efficiently harvest excitons that then get transferred by Förster resonance energy transfer (FRET) to an MR‐TADF moiety responsible for the narrowband emission. Compound **DtBuCzB** was selected as the MR‐TADF emitting core thanks to its fast radiative rate (k_r_ ≈10^8^ s^−1^), and narrowband emission (λ_PL_ = 483 nm, FWHM = 23 nm in toluene solution 10^−5^ M),^[^ ^35^ ^]^ while this moiety was sandwiched between to TADF dendrimers, **2GtBuCzCO_2_H**, bearing second‐generation *tert*‐butyl carbazole donor dendrons^[^ ^6^ ^]^ linked to a benzoate acceptor. These dendrimer groups in **2GtBuCzCO_2_HDCzB** act to shield the MR‐TADF core from intermolecular interactions (**Figure** **1**). For comparison, we also designed **tBuCzCO_2_HDCzB**, which only contains *tert*‐butyl carbazoles that are less effective at encapsulating the MR‐TADF emissive core. In 30 wt% doped film in mCP, **2GtBuCzCO_2_HDCzB** and **tBuCzCO_2_HDCzB** possess similarly high Φ_PL_ of 98 and 94%, Δ*E*_ST_ of 0.14 and 0.15 eV, τ_d_ of 102 and 143 µs, and *k*_RISC_ of 2.37 × 10^4^ and 1.23 × 10^4^ s^−1^. The combination of the *ortho‐*carbazole‐based donors and the *para* caboxyl group on the pendant phenyl ring of the MR‐TADF core **DtBuCzB** endows both emitters simultaneously with a strong resistance to ACQ and more efficient exciton harvesting, which translates to solution‐processed devices that show both higher EQE_max_ and lower efficiency roll‐off. Compared to the *tert‐*butyl carbazole‐substituted **tBuCzCO_2_HDCzB**, **2GtBuCzCO_2_HDCzB** exhibits improved resistance to aggregation and ACQ as well as a resistance to bimolecular exciton quenching processes in the SP‐OLEDs. The SP‐OLEDs with 30 wt% **2GtBuCzCO_2_HDCzB** and **tBuCzCO_2_HDCzB** in the EML showed high EQE_max_ of 27.9 and 22.0% and low efficiency roll‐off, with EQE_5000_ of 22.3 and 16.3%, respectively. Moreover, the EQE_max_ of the host‐free SP‐OLEDs with **2GtBuCzCO_2_HDCzB** remained high at 24.0% while the efficiency roll‐off remained low (EQE_1000_ of 20.2%), while the host‐free devices with **tBuCzCO_2_HDCzB** performed much worse, reflected in the much lower EQE_max_ of 11.4%.

## 2 Results and Discussion

### 2.1 Synthesis

The synthesis is outlined in Schemes S1 and S2 (Supporting Information). The intermediate **DCzB‐Bpin** was reacted with 4‐bromo‐3,5‐difluorobenzonitrile under Suzuki‐Miyaura cross‐coupling conditions to afford **DCzBDFCN** in 96% yield. **DCzBDFCN** was then reacted with 3,6‐di‐tert‐butylcarbazole and **2GtBuCz** under S_N_Ar conditions to obtain the target compounds, **2GtBuCzCO_2_HDCzB** and **tBuCzCO_2_HDCzB,** in 60 and 38% yield, respectively. The two emitters were purified by silica gel chromatography followed by gel permeation chromatography. During the S_N_Ar reaction, the benzonitrile was hydrolysed to the benzoate in both emitters, which was confirmed by high‐resolution mass spectrometry (HRMS) and elemental analysis (Figures S16 and S20, Supporting Information). By contrast, for **tBuCzCN** and **2GBuCzCN** the benzonitrile functionality was preserved under the S_N_Ar reaction conditions, albeit the product yields were low, especially for **2GBuCzCN** (yield: 9%). To more closely match the electronics of the structures of these model compounds with those of the emitters, we hydrolysed the nitrile groups of **tBuCzCN** and **2GBuCzCN**; however, only **tBuCzCO_2_H** could be isolated as a pure compound, in 82% yield. Given the small amounts of **2GBuCzCN** remaining and the established similar properties between this compound and **2GBuCzCO_2_H** (vide infra) we opted to not pursue the synthesis of **2GBuCzCO_2_H**. The structure and purity of **2GtBuCzCO_2_HDCzB** and **tBuCzCO_2_HDCzB** were confirmed by ^1^H and ^13^C NMR spectroscopy, high‐resolution mass spectrometry (HRMS), high‐performance liquid chromatography (HPLC), and elemental analysis (Figures S2–S21, Supporting Information).

### 2.2 Theoretical Calculations

**Figure 2:**
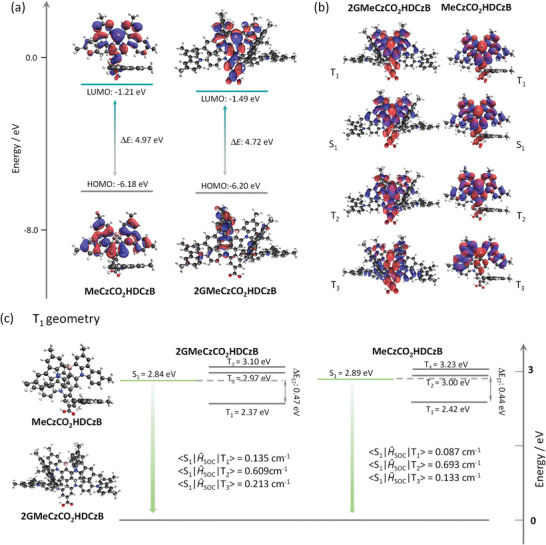
a) Distributions of the frontier molecular orbitals (isovalue: 0.02) of **2GMeCzCO_2_HDCzB** and **MeCzCO_2_HDCzB**, calculated at the M06‐2X/6‐31G(d,p) level in the gas phase. b) The natural transition orbitals (NTOs) (isovalue: 0.02) for S_1_, T_1_, T_2_, and T_3_ for **2GMeCzCO_2_HDCzB** and **MeCzCO_2_HDCzB** at the TDA‐DFT‐M06‐2X/6‐31G(d,p) in the gas phase. c) Optimized triplet‐state geometry and spin‐orbit coupling matrix element (SOCME) for **2GMeCzCO_2_HDCzB** and **MeCzCO_2_HDCzB** based on the optimized T_1_ geometry, calculated at the uM06‐2X/6‐31G(d,p) level in the gas phase.

A theoretical study was first undertaken to obtain insight into the optoelectronic properties of these compounds and to rationalize the proposed design. To reduce the computational cost, we computed the properties of model compounds **MeCzCO_2_H**, **2GMeCzCO_2_H**, **DCzB**, **MeCzCO_2_HDCzB**, and **2GMeCzCO_2_HDCzB** (Figure S22, Supporting Information) where the peripheral *tert*‐butyl groups have been replaced with methyl groups; notably, there is essentially no change in the electron density distributions or energies of **MeCzCO_2_HDCzB** and **tBuCzCO_2_HDCzB** (Figure S23, Supporting Information) and so these are appropriate model compounds. This structural change is not expected to lead to significant differences in the optoelectronic properties of monomolecular species in the gas phase calculations. The calculations were carried out using Density Functional Theory at the M06‐2X/6‐31G(d,p) level of theory in the gas phase and the relevant data were summarized in Table S1 (Supporting Information). The ground states of the reported emitting core **DCzB**, and the two new MR‐TADF emitters **MeCzCO_2_HDCzB** and **2GMeCzCO_2_HDCzB** were first optimized starting from a Chem3D generated structure. For **MeCzCO_2_HDCzB**, the HOMO is located on the **DCzB** core while the LUMO is distributed both on the **DCzB** moiety and the benzoate. For **2GMeCzCO_2_HDCzB**, the HOMO and the degenerate HOMO‐1 are located on separate donor dendrons, while as with **MeCzCO_2_HDCzB** the LUMO is located on both the **DCzB** and the benzoate moieties. The HOMO of **MeCzCO_2_HDCzB** and **2GMeCzCO_2_HDCzB** are predicted to be ‐6.18 and ‐6.20 eV, respectively, which are destabilized compared to the predicted HOMO for **DCzB** (−6.42 eV). This is due to the presence of the electron‐withdrawing benzoate in the former two compounds. The LUMOs are stabilized to −1.21 and −1.39 eV, respectively, compared to −1.14 eV for **DCzB** (Figure S24, Supporting Information), which is attributed to the presence of the electron‐withdrawing benzoate in the former two compounds. The S_1_/T_1_ states of **MeCzCO_2_HDCzB** and **2GMeCzCO_2_HDCzB** are predicted to be 3.16/2.71 and 3.15/2.69 eV, both of which are stabilized compared to **DCzB** (3.40/2.91 eV), resulting in a large Δ*E*_ST_ of 0.46/0.46 eV of similar magnitude to that of **DCzB** (0.49 eV). At the ADC(2)/cc‐pVDZ level of theory, which we had previously shown to accurately predict the excited‐state energies of MR‐TADF compounds,^[^ ^36^, ^37^ ^]^ the Δ*E*_ST_ of **DCzB** is 0.13 eV (Figure S25, Supporting Information), which matches the experimental Δ*E*_ST_ of 0.13 eV for **DtBuCzB** in (Figure S30, Supporting Information). The size of **MeCzCO_2_HDCzB** and **2GMeCzCO_2_HDCzB** precludes the use of this higher level of theory, but we can infer from the similar DFT calculated Δ*E*_ST_ values to that of **DCzB** that these two compounds will likewise have comparably small singlet‐triplet gaps. The natural transition orbitals (NTOs) at the optimized T_1_ geometry for the S_1_ and T_1_ states of both **MeCzCO_2_HDCzB** and **2GMeCzCO_2_HDCzB** are primarily located on the **DCzB** core and show the characteristic alternating pattern of increasing and decreasing electron density in the excited states compared to the ground state that describes a short‐range charge transfer (SRCT) excited state (Figure 2b). The NTOs of the closely lying T_2_ state is distributed across on the central benzene ring of the MR‐TADF core and the benzoate, and that of T_3_ is localized on the carbazole of the MR‐TADF core of **MeCzCO_2_HDCzB** and has LRCT character for **2GMeCzCO_2_HDCzB**. The spin‐orbital coupling matrix element (SOCME) between S_1,_ and T_1_, performed based on the T_1_ state geometry, are 0.135 and 0.087 cm^−1^ for **2GMeCzCO_2_HDCzB** and **MeCzCO_2_HDCzB,** respectively, while those between two close‐lying triplet states and S_1_ are generally much larger at 0.609 and 0.213 cm^−1^ for **2GMeCzCO_2_HDCzB** and 0.693 and 0.133 cm^−1^ for **MeCzCO_2_HDCzB,** indicating a RISC mechanism that is likely to occur via spin‐vibronic coupling involving one or more these states.^[^ ^36^ ^]^ The photoluminescence FWHM can inferred from the reorganization energy (λ). The similarly small calculated λ values of 0.11, 0.22, and 0.24 eV were obtained for **DCzB**, **2GMeCzCO_2_HDCzB**, and **MeCzCO_2_HDCzB** (Figure S23, Supporting Information), indicating that narrowband emission is expected for these compounds.^[^ ^39^, ^40^ ^]^ The relatively larger λ values for **2GMeCzCO_2_HDCzB** and **MeCzCO_2_HDCzB** are attributed to the increased flexibility of D‐A TADF pendants compared to the more rigid DCzB structure.

To substantiate the emitter design, we modeled two fragments of the parent emitters, **2GMeCzCO_2_H** and **MeCzCO_2_H** (Figure S26, Supporting Information), emulating the D‐A part of the compounds. This was done to verify whether their energy levels were higher than those of the MR‐TADF core, **DMeCzB**, such that energy transfer between the two fragments would be possible. The HOMO of **MeCzCO_2_H** is located on carbazoles and the benzene ring, while for **2GMeCzCO_2_H** the HOMO is localized on the carbazole donor dendrons; the LUMOs for both molecules are located on the benzoate moieties. The values of the HOMO and LUMO are −6.35 and −1.10 eV for **2GMeCzCO_2_H** and −6.67 and −0.74 eV for **MeCzCO_2_H**, respectively. The S_1_/T_1_ states of **MeCzCO_2_H** and **2GMeCzCO_2_H** are 4.05/3.60 and 3.93/3.45 eV, which are sufficiently higher in energy than those of the MR‐TADF core **DCzB** (3.40/2.91 eV), thus indicating that there should be a thermodynamic driving force for energy transfer from the D‐A fragment to the MR‐TADF core.

### 2.3 Electrochemistry

We next investigated the optoelectronic properties in dilute DMF solution. The energies of FMOs were inferred from the electrochemistry, where the oxidation and reduction potentials (*E*_ox_/*E*_red_) were obtained using cyclic voltammetry (CV) and differential pulse voltammetry (DPV) in deaerated DMF (Figure S27, Supporting Information). **2GtBuCzCO_2_HDCzB** exhibits two irreversible oxidation waves with *E*_ox_ of 1.06 and 1.21 V determined from the DPV peak values, which are ascribed to the oxidation of **DtBuCzB** core (1.03 V) and the peripheral carbazole donor dendron (1.19 V),^[^ ^6^ ^]^ respectively. The *E*_ox_ values for **tBuCzCO_2_HDCzB** are similar at 1.01 and 1.17 V, respectively. **2GtBuCzCO_2_HDCzB** and **tBuCzCO2HDCzB** show reversible reduction waves at *E*_red_ of −1.71 and −1.61 V, determined from the DPV peak values, respectively, which are stabilized compared to that of **DtBuCzB** (−1.73 V), ascribed to the reduction of the benzoate‐substituted **DtBuCzB**. The HOMOs for **2GtBuCzCO_2_HDCzB** and **tBuCzCO_2_HDCzB** are calculated from the first *E*_ox_ and the corresponding HOMO/LUMO values are −5.40/−2.63 eV and −5.35/−2.73 eV, leading to a HOMO‐LUMO gap of 2.77 and 2.62 eV, for **2GtBuCzCO_2_HDCzB** and **tBuCzCO_2_HDCzB**, respectively.

### 2.4 Photophysics

**Figure 3:**
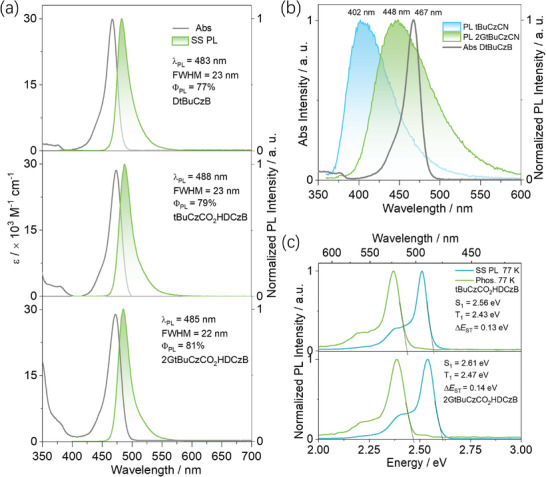
a) Absorption and steady‐state PL (SS‐PL) spectra of **DtBuCzB**, **tBuCzCO_2_HDCzB** and **2GtBuCzCO_2_HDCzB** in dilute toluene at room temperature (λ_exc_  =  340 nm); b) Absorption and steady‐state PL spectra of **DtBuCzB** and **tBuCzCN** and **2GtBuCzCN** in toluene at a concentration of 10^−5^ M, respectively. c) Steady‐state PL (77 K) and phosphorescence spectra (1–10 ms, 77 K) of **tBuCzCO_2_HDCzB** and **2GtBuCzCO_2_HDCzB** in toluene (λ_exc_  =  340 nm).

The photophysical properties of **2GtBuCzCO_2_HDCzB** and **tBuCzCO_2_HDCzB** in solution were first investigated. The UV–vis absorption spectra of **2GtBuCzCO_2_HDCzB** and **tBuCzCO_2_HDCzB** in toluene show an identical intense (ε ≈ 29 × 10^3^ M^−1^ cm^−1^) SRCT transition at ≈470 nm as that of **DtBuCzB** (**Figure** **3**a), which indicates that the electronics of this state are not perturbed by the presence of the donor dendrons. The higher energy band (300–400 nm) of **tBuCzCO_2_HDCzB** and **2GtBuCzCO_2_HDCzB** is more intense compared to that of **DtBuCzB**, which arises from the contribution from a π−π* transition localized on the carbazole donor dendrons. The steady‐state PL spectra of **2GtBuCzCO_2_HDCzB** and **tBuCzCO_2_HDCzB** are narrow (FWHM of 21 and 22 nm) and peak at λ_PL_ of 485 and 487 nm, respectively, which are almost identical to the emission of **DtBuCzB** (λ_PL_ = 483 nm with FWHM of 23 nm). **DtBuCzB**, **tBuCzCO_2_HDCzB**, and **2GtBuCzCO_2_HDCzB** have similar photoluminescence quantum yields (Φ_PL_) of 77, 79, and 81% (Figure S41, Supporting Information), respectively, in degassed toluene solution. The small Stokes shift of 15 and 13 nm for **tBuCzCO_2_HDCzB**, and **2GtBuCzCO_2_HDCzB** in toluene, respectively and the modest positive solvatochromism (Figure S29, Supporting Information) are characteristic of an emissive state of SRCT character.

**tBuCzCO_2_H** and **tBuCzCN** have similar emission spectra (Figure S28, Supporting Information) and excited‐state energies at 77 K in toluene (S_1_/T_1_ = 3.38/3.02 eV and S_1_/T_1_ = 3.38/3.03 eV, respectively), indicating that the benzoate and benzonitrile groups similarly affect the photophysical properties of these two compounds. Therefore, **2GtBuCzCN** and **tBuCzCN** can be employed as components in the subsequent energy transfer process study. As shown in Figure 1, **2GtBuCzCN** and **tBuCzCN** emit at λ_PL_ of 448 and 402 nm, have large FWHM of 79 and 62 nm, and low photoluminescence quantum yields, Φ_PL_, of 33 and 19% (Figure S41, Supporting Information), respectively. The emission spectra of **2GtBuCzCN** and **tBuCzCN** largely overlap with the absorption spectrum of **DtBuCzB** (λ_abs_ = 467 nm, Figure 3b), meaning that FRET is expected to be efficiency between each of these two pairs of molecules.

The energies of the S_1_/T_1_ energies of **2GtBuCzCO_2_HDCzB** and **tBuCzCO_2_HDCzB** were extracted from the onsets of the steady‐state photoluminescence and delayed emission spectra in frozen toluene in 77 K, while the Δ*E*_ST_ is the energy difference between these two states. The S_1_/T_1_/Δ*E*_ST_ energies of **2GtBuCzCO_2_HDCzB** and **tBuCzCO_2_HDCzB** are 2.61/2.47/0.14 and 2.56/2.43/0.13 eV, respectively (Figure 3c), which are similar to those of **DtBuCzB** (2.57/2.44/0.13 eV in toluene at 77 K, Figure S30, Supporting Information). The similar energies of these low‐lying excited states indicate that the pedant **tBuCzCO_2_H** and **2GtBuCzCO_2_H** moieties do not materially affect the electronics of the emissive MR‐TADF core in **2GtBuCzCO_2_HDCzB** and **tBuCzCO_2_HDCzB**. This is due to the higher excited‐state energies of the **tBuCzCO_2_H** and **2GtBuCzCO_2_H**, which was confirmed by an analysis of the excited‐state energies of the related model compounds **tBuCzCN** and **tBuCzCN** (Figure S31, Supporting Information). Notably, delayed emission is detected in toluene for **2GtBuCzCO_2_HDCzB** and **tBuCzCO_2_HDCzB**, with τ_d_ of 38 and 34 µs, respectively, which is absent in **DtBuCzB** (Figure S32, Supporting Information).

**Figure 4:**
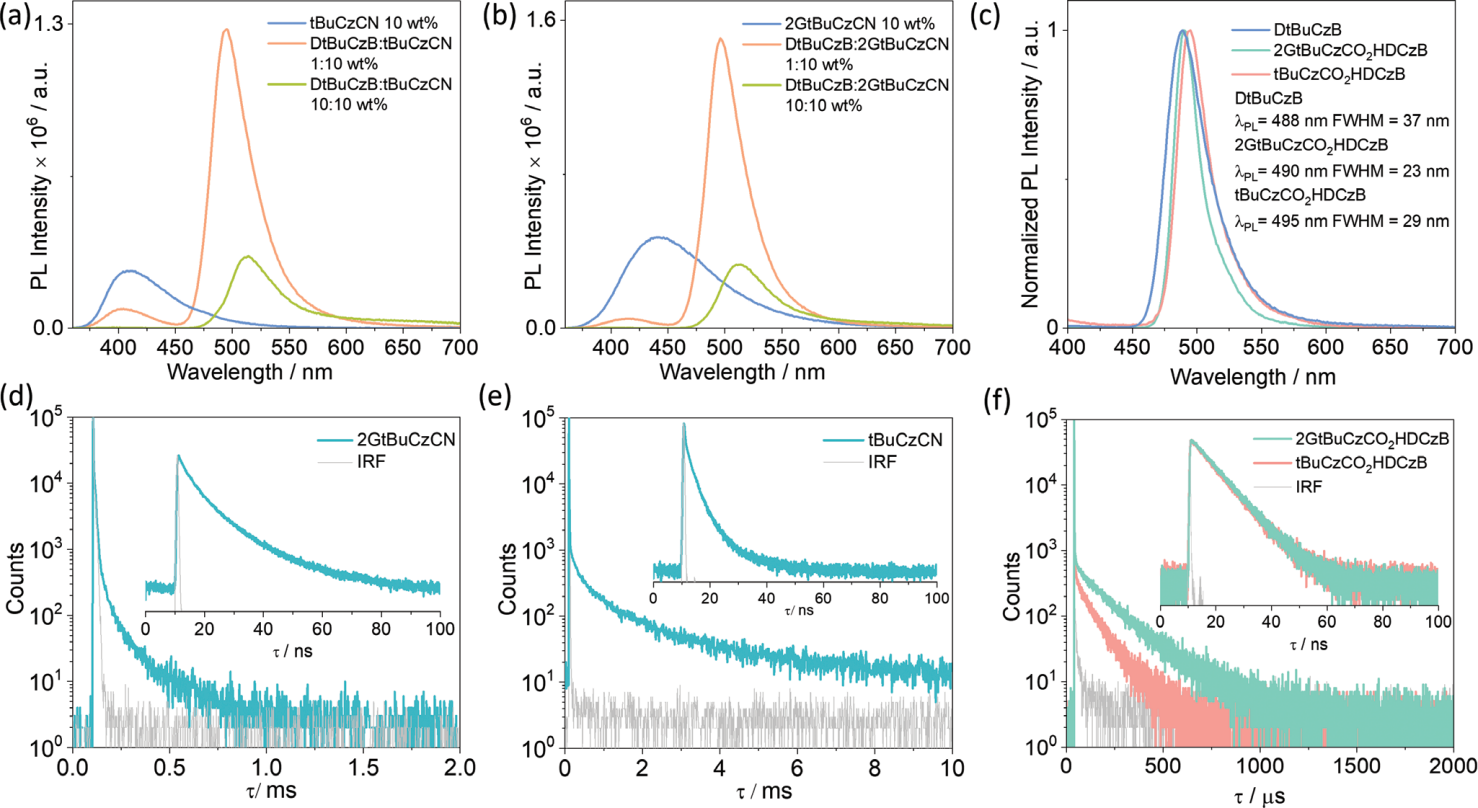
Steady‐state PL of a) **tBuCzCN** and b) **2GtBuCzCN** in 10 wt% PMMA and the co‐doped film of **DtBuCzB**: **tBuCzCN**/**2GtBuCzC** with a ratio of 1:10 and 10:10 wt% PMMA film. c) Steady‐state PL spectra of 1 wt% doped film of **tBuCzCO_2_HDCzB** and **2GtBuCzCO_2_HDCzB** in PMMA, λ_exc_ = 340 nm. Time‐resolved PL decays of d) **2GtBuCzCN**, e) **tBuCzCN**, and f) **2GtBuCzCO_2_HDCzB** and **tBuCzCO_2_HDCzB** in 1 wt% doped film in PMMA film, λ_exc_ = 375 nm.

We next investigated the photophysical properties of 1 wt% spin‐coated doped thin films in PMMA, which would permit the interrogation of monomolecular species in the solid state and preclude the host‐guest interaction (Table S3, Supporting Information). FRET efficiencies between **2GtBuCzCN** or **tBuCzCN** and **DtBuCzB** were calculated using the co‐doped **2GtBuCzCN**/**DtBuCzB** and **tBuCzCN**/**DtBuCzB** films. The emission intensity of **2GtBuCzCN** (λ_PL_: 428 nm, 1 wt% doped film in PMMA) significantly drops in the **2GtBuCzCN**/**DtBuCzB** (1:1) doped films in PMMA, while the intensity of the emission of **tBuCzCN** (λ_PL_: 402 nm, 1 wt% doped film in PMMA) decreases only a little in the **tBuCzCN**/**DtBuCzB** (1:1) doped films (Figure S33, Supporting Information). Based on the emission intensity changes observed for **2GtBuCzCN** or **tBuCzCN** in their binary and ternary films,^[^ ^23^ ^]^ the FRET efficiencies were calculated to be 85 and 33%, respectively, which are consistent with the degree of spectral overlap (Figure 3b), indicating a higher FRET efficiency for the **2GtBuCzCN**/**DtBuCzB** system. In the more concentrated 10:10 wt% doped films of **2GtBuCzCN/DtBuCzB** and **tBuCzCN**/**DtBuCzB** in PMMA, the FRET efficiencies increased to 100 and 99% (**Figure** **4**a,b), respectively. Given the short distance between the peripheral **2GtBuCzCN**/**DCzCN** moieties and the central **DtBuCzB** emissive group, the FRET efficiencies in **2GtBuCzCO_2_HDCzB** and **tBuCzCO_2_HDCzB** are quantitative, reflective in the single observed narrowband emission at λ_PL_ of 490 (FWHM of 23 nm) and 495 nm (FWHM of 29 nm), respectively. A quantitative energy transfer was also observed in the 10:10 wt% doped films of **tBuCzCO_2_H/DtBuCzB** in PMMA (Figure S34, Supporting Information). The PL spectra are narrower compared to that of **DtBuCzB** (λ_PL_ of 488 nm FWHM of 37 nm) as shown in Figure 4c. The S_1_ energies of **2GtBuCzCO_2_HDCzB** (2.57 eV) and **tBuCzCO_2_HDCzB** (2.56 eV) were inferred from the high‐energy onset of the steady‐state PL at 77 K, while the T_1_ energies, determined from the onset of the time‐gated (1‐10 ms) emission at 77 K, are 2.43 and 2.43 eV for **2GtBuCzCO_2_HDCzB** and **tBuCzCO_2_HDCzB**, respectively. The corresponding Δ*E*_ST_ values are 0.14 and 0.13 eV, respectively (Figure S35, Supporting Information). The S_1_/T_1_ energies of **2GtBuCzCO_2_HDCzB** and **tBuCzCO_2_HDCzB** are very similar to those of **DtBuCzB** (S_1_/T_1_ = 2.62/2.49 eV, Δ*E*_ST_ = 0.13 eV, Figure S36, Supporting Information) measured under same conditions. This alignment of the state energies suggests that the emission of **2GtBuCzCO_2_HDCzB** and **tBuCzCO_2_HDCzB** originates from the emitting **DtBuCzB** core. The S_1_/T_1_ energies of **2GtBuCzCN** and **tBuCzCN** are 3.20/2.97 eV and 3.34/2.98 eV (Figure S37, Supporting Information), respectively, measured under the same conditions, with corresponding Δ*E*_ST_ of 0.23 and 0.36 eV. This implies that *k*_RISC_ in **2GtBuCzCN** will be faster than in **tBuCzCN** and thus the former will be a much more efficient exciton harvester, reflected in their respective diverging τ_d_ of 80.4 µs and 1.1 ms (Figure 4). The S_1_/T_1_ energies and Δ*E*_ST_ of **tBuCzCN** are also almost identical to those of **tBuCzCO_2_H** (3.39/3.02 eV and 0.37 eV, Figure S38, Supporting Information), thus leading to both compounds having similar τ_d_ of 0.9 ms (Figure S39, Supporting Information), which are similar to those values in toluene. The higher energies of these states than those of **DtBuCzB** explain the origin of the efficient FRET between the two moieties within **2GtBuCzCO_2_HDCzB** and **tBuCzCO_2_HDCzB**. In addition, **2GtBuCzCO_2_HDCzB** and **tBuCzCO_2_HDCzB** have higher Φ_PL_ of 71 and 56% than **DtBuCzB** (43%) and a greater amount of delayed fluorescence (Φ_d_ of 34 and 17% for **2GtBuCzCO_2_HDCzB** and **tBuCzCO_2_HDCzB**) compared to **DtBuCzB** (Φ_d_ of 6%) in 1 wt% doped film in PMMA, indicating suppressed non‐radiative due to the steric encapsulation from the pendant donor dendron moieties. This is also reflected in the relative magnitudes of τ_d_, increasing from 85 µs for **DtBuCzB** (Figure S40, Supporting Information) to τ_d_ of 91 and 159 µs for **tBuCzCO_2_HDCzB** and **2GtBuCzCO_2_HDCzB**, respectively, in 1 wt% doped film in PMMA. The calculated kinetics constants reveal that the *k*_RISC_ of 9.17×10^3^ and 7.85×10^3^ s^−1^for **2GtBuCzCO_2_HDCzB** and **tBuCzCO_2_HDCzB** are faster compared to 3.03 ×10^3^ s^−1^ for **DtBuCzB**.

**Figure 5:**
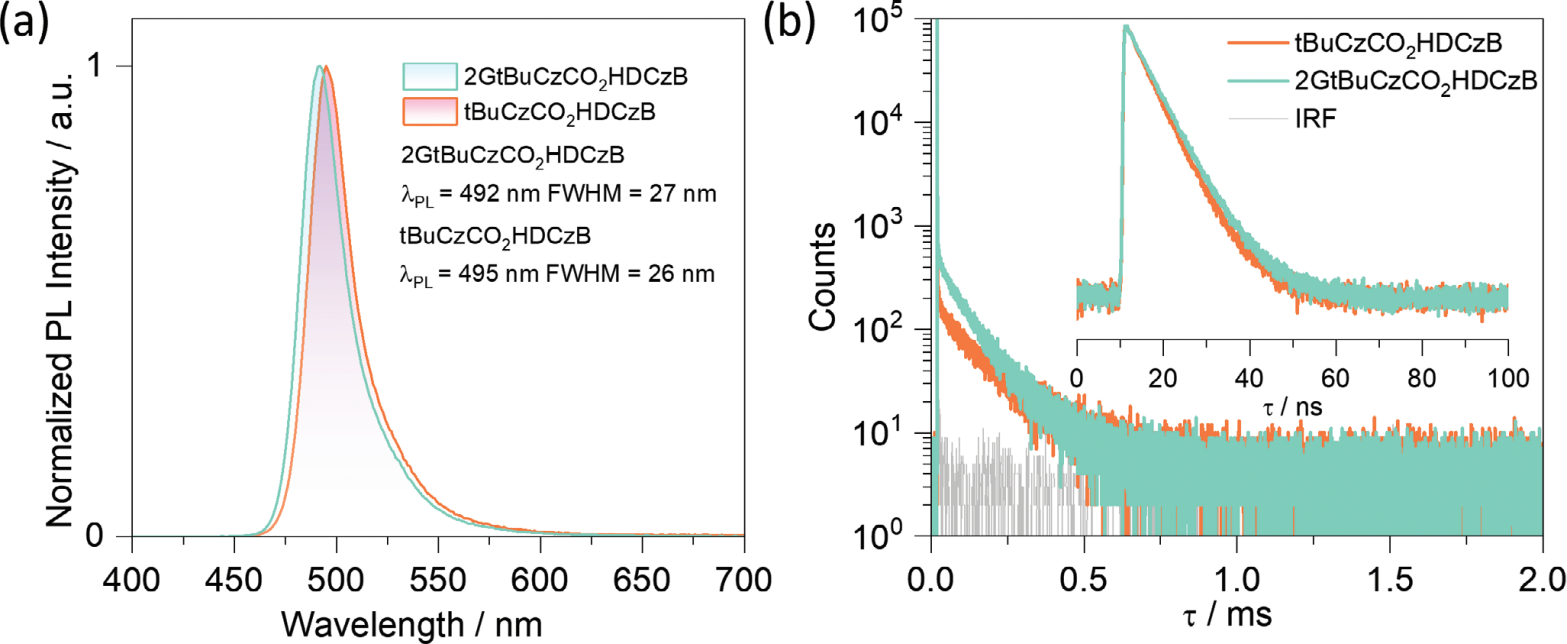
a) Steady‐state PL of **tBuCzCO_2_HDCzB** and **2GtBuCzCO_2_HDCzB** in 30 wt% doped films in *m*CP, λ_exc_ = 340 nm. b) Time‐resolved PL decays of **tBuCzCO_2_HDCzB** and **2GtBuCzCO_2_HDCzB** in 30 wt% doped films in *m*CP, λ_exc_ = 375 nm.

With a vision for using these emitters in OLEDs, we next investigated the photophysical properties in *m*CP, which was identified as the best host material because of its suitably aligned HOMO/LUMO energy levels with the adjacent layers in the OLED stack (vide infra), its high triplet energy (3.0 eV), and the high‐quality and homogeneous films that could be prepared by spin‐coating.^[^ ^41^ ^]^ Benefitting from the “encapsulated” nature of their structures, the PLs of both **2GtBuCzCO_2_HDCzB** and **tBuCzCO_2_HDCzB** showed resistance to concentration quenching (Figure S42, Supporting Information), as the doping concentration increased from 1 to 70 wt% in mCP films, no excimer emission was observed. Impressively, the narrowband emission of **2GtBuCzCO_2_HDCzB** and **tBuCzCO_2_HDCzB** observed in the 1 wt% doped film in PMMA is conserved in the 70 wt% doped films, with λ_PL_/FWHM of 496/31 and 499/29 nm, respectively (Figure S43, Supporting Information). The highest Φ_PL_ values were achieved at 30 wt% doping at 98 and 94% for **2GtBuCzCO_2_HDCzB** and **tBuCzCO_2_HDCzB**, respectively, at this doping level, the λ_PL_/FWHM are 492/27 and 495/26 nm (**Figure** **5**a and **Table** 1). Similarly, in these films **2GtBuCzCO_2_HDCzB** and **tBuCzCO_2_HDCzB** also maintain their small Δ*E*_ST_ of 0.14 and 0.15 eV, respectively (Figure S46, Supporting Information). The average prompt, τ_p_, and delayed, τ_d_, lifetimes are 7.0 ns and 101.8 µs for **2GtBuCzCO_2_HDCzB** and 6.4 ns and 143 µs for **tBuCzCO_2_HDCzB**. The corresponding *k*_ISC_ are 8.57×10^7^ and 7.81×10^7^ s^−1^ and *k*_RISC_ are 2.37 ×10^4^ and 1.23×10^4^ s^−1^ for **2GtBuCzCO_2_HDCzB** and **tBuCzCO_2_HDCzB**, respectively, which are faster than the *k*_RISC_ in PMMA doped film, which can be attributed to different host‐guest interactions that affect the Δ*E*_ST_ of the (Table 1) emitter.^[^ ^41^ ^]^

**Table 1:** Photophysical data of **tBuCzCO_2_HDCzB** and **2GtBuCzCO_2_HDCzB** in 30 wt% doped films in mCP.

Emitter	λ_PL_/nm^a)^	Φ_PL_/%^b)^	FWHM/nm^c)^	S_1_/eV^d)^	T_1_/eV^e)^	Δ*E*_ST_/eV^f)^	τ_p_/ns^g)^	τ_d_/µs^g)^	*k*_RISC_/10^4^ s^−1^ ^h)^
**tBuCzCO_2_HDCzB**	495	94	26	2.52	2.37	0.15	6.4	143	1.23
**2GtBuCzCO_2_HDCzB**	492	98	27	2.55	2.41	0.14	7.0	102	2.37

^a)^ Obtained at 298 K, λ_exc_ = 340 nm;

^b)^ The Φ_PL_ was measured in an integrating sphere under nitrogen (λ_exc_ = 340 nm);

^c)^ Full‐width at half‐maximum;

^d)^ Obtained from the onset of the SS PL spectrum at 77 K;

^e)^ Obtained from the onset of the delayed emission spectrum (1–10 ms) at 77 K (λ_exc_ = 340 nm);

^f)^ Δ*E*_ST_ = *E*(S_1_) – *E*(T_1_);

^g)^ Measured at 300 K under vacuum, λ_exc_ = 375 nm;

^h)^ Rate constant for reverse intersystem crossing, details are shown in Table S4 (Supporting Information).

### 2.5 Electroluminescence Properties

**Figure 6:**
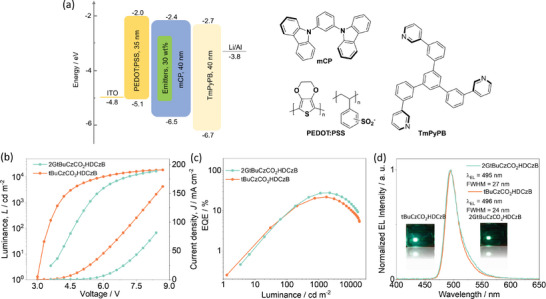
Electroluminescence characteristics of the OLED based on 30 wt% **tBuCzCO_2_HDCzB** and **2GtBuCzCO_2_HDCzB** in *m*CP. a) Device configuration, and the materials employed in the devices. b) Current density and luminance versus voltage characteristics for the devices. c) EQE versus brightness. d) Electroluminescence spectra of the devices, insets are photos of the operational devices.

We finally fabricated solution‐processed OLEDs using the following device stack: ITO/PEDOT:PSS (35 nm)/*m*CP: 30% emitters (40 nm)/1,3,5‐tri(*m*‐pyrid‐3‐yl‐phenyl)benzene (TmPyPB) (40 nm)/LiF (1 nm)/Al (100 nm), where PEDOT:PSS was used as the hole injection and transport layer, TmPyPB was used as electron transport layer and LiF was selected as electron injection layer, the latter two being vacuum deposited on top of the emissive layer. The device structures and energy level of each layer are illustrated in **Figure** **6**a. The devices with 30 wt% in *m*CP host were first fabricated as high Φ_PL_ were measured for both emitters at this doping concentration. The device performance is shown in Figures 6 and S47 (Supporting Information) and the data collected in **Table** **2**.

**Table 2:** Electroluminescence data for the devices.

Emitter	λ_EL_ ^a)^/nm	FWHM/nm	CIE^b)^/(x.y)	V_on_ ^c)^/V	CE_max_/cd A^−1^	PE_max_/lm W^−1^	L_max_ ^d)^/cd m^−2^	EQE^e)^/%
**2GtBuCzCO_2_HDCzB**								
30 wt% in mCP	495	27	0.08, 0.53	3.4	61.2	35.0	16 686	27.9/27.6/22.3
neat with 30 wt% OXD7	495	30	0.14, 0.54	2.9	57.3	47.3	12 070	24.0/20.2/10.8

**tBuCzCO_2_HDCzB**								
30 wt% in mCP	496	24	0.09, 0.53	3.0	47.3	35.4	18 754	22.0/21.4/16.3
neat with 30 wt% OXD7	498	27	0.15, 0.56	2.8	28.3	22.7	7784	11.4/9.5/3.4

^a)^ Electroluminescence emission peak at 4 V;

^b)^ Commission Internationale de L’Éclairage coordinates;

^c)^ The turn‐on voltage;

^d)^ Luminance maximum (*L*_max_) measured at highest voltage;

^e)^ For device with 30 wt% in mCP: the EQE_max_/EQE_2000_/EQE_5000_, for device neat with 30 wt% OXD7: EQE_max_/EQE_1000_/EQE_5000._

As shown in Figure 6d, the devices with **2GtBuCzCO_2_HDCzB** and **tBuCzCO_2_HDCzB** emitted at λ_EL_ of 495 nm (FWHM = 27 nm) and 496 nm (FWHM = 24 nm), producing green OLEDs with CIE coordinates of (0.08, 0.53) and CIE of (0.09, 0.53), respectively. The EL matches that of the PL, indicating that at this doping concentration there is no significant aggregation in the device (Figure S48, Supporting Information). The devices with **2GtBuCzCO_2_HDCzB** and **tBuCzCO_2_HDCzB** both exhibited low EQE at low luminance. This EQE versus luminance behavior can be attributed to the lower packing density of the emitters in the spin‐coated EMLs, which results in decreased carrier mobility and poor carrier balance.^[^ ^43^, ^44^ ^]^ Consequently, at lower driving voltages, only a portion of the emitters are activated, leading to suboptimal exciton utilization and, ultimately, lower EQE values, this phenomenon has also been observed in many reported dendrimer emitter‐based SP‐OLEDs.^[^ ^6^, ^45^, ^46^, ^47^, ^48^, ^49^ ^]^ The device with **2GtBuCzCO_2_HDCzB** achieved a high EQE_max_ of 27.9% at a luminance of 2188 cd m^−2^; the EQE drops to only 27.6% at 2000 cd m^−2^. Even at 5000 cd m^2^, the efficiency roll‐off remains low at 20% (EQE_5000_ of 22.3%). This device performance is one of the best for solution‐processed green TADF OLEDs, and the best amongst solution‐processed green MR‐TADF devices (Table S5 and Figure S52, Supporting Information). In contrast, the device with **tBuCzCO_2_HDCzB** showed an EQE_max_ of 22.0% at 1741 cd m^−2^, the EQE remained at 21.4% at 2000 cd m^−2^ and dropped to 16.3% at 5000 cd m^−2^, representing an efficiency roll‐off of 26%. Maximum luminance, *L*_max_, values of 16 686 and 18 754 cd m^−2^ were achieved for the devices with **2GtBuCzCO_2_HDCzB** and **tBuCzCO_2_HDCzB**, respectively.

**Figure 7:**
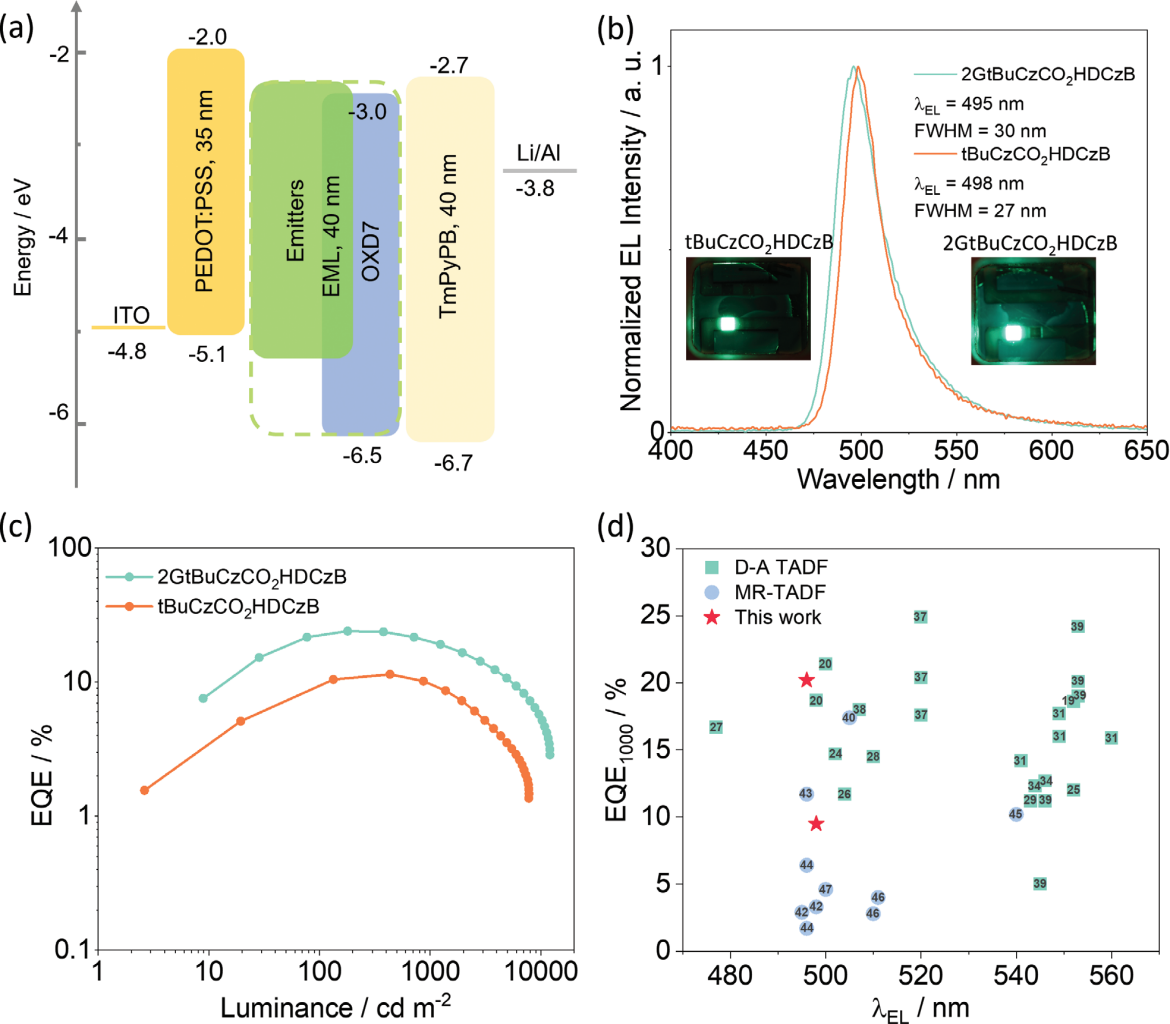
Electroluminescence characteristics of the OLED based on **tBuCzCO_2_HDCzB** and **2GtBuCzCO_2_HDCzB** with 30 wt% OXD7. a) Device configuration. b) Electroluminescence spectra of the devices. c) EQE versus brightness. d) EQE_1000_ of reported solution‐processed green TADF and MR‐TADF OLEDs as a function of λ_EL_ (Table S5, Supporting Information, summarizes the associated data used to construct this figure).

Recognizing the benefits of the “encapsuled” structure and the high Φ_PL_ of **2GtBuCzCO_2_HDCzB** at high doping concentrations, the host‐free solution‐processed devices were then fabricated, where the emissive layer consisted of 70 wt% emitters plus 30wt% of the electron‐transporting l,3‐bis[2‐(4‐*tert*‐butylphenyl)‐1,3,4‐oxadiazo‐5‐yl]benzene (OXD‐7) to aid in charge balance; indeed, we have shown this EML composition to be an effective strategy in high‐performance solution‐processed dendrimer OLEDs.^[^ ^6^ ^]^ The device structure is shown in **Figure** **7**a. The photophysical properties of the 70 wt% emitter/30 wt% OXD‐7 film are shown in Figure S49 (Supporting Information). The host‐free device with **2GtBuCzCO_2_HDCzB** emits at 496 nm (FWHM of 30 nm), Figure 7b, with associated CIE coordinates of (0.14, 0.54). The EL spectrum aligns with the PL spectrum (Figure S51, Supporting Information). The device with **2GtBuCzCO_2_HDCzB** showed an EQE_max_ of 24.0% (at 180 cd m^−2^, Figure 7c), which is comparable to our previously reported non‐doped SP‐OLEDs with the TADF dendrimer **tBuCz2m2pTRZ** (EQE_max_ of 28.4%).^[^ ^6^ ^]^ Moreover, this device showed a low efficiency roll‐off of 16%, with an EQE of 20.2% at 1000 cd m^−2^, which is better than that of SP‐OLED with **tBuCz2m2pTRZ** where at 500 cd m^−^^2^ the efficiency roll‐off was 14% (EQE_500_ of 22.7%).^[^ ^6^ ^]^ Although the device with **tBuCzCO_2_HDCzB** can also show narrowband green emission with a λ_EL_ at 498 nm and CIE coordinates of (0.15, 0.56), the EQE_max_ decreased significantly to 12.5% at 433 cd m^−2^ and the EQE_1000_ was only 9.5%. The EQE of the non‐doped device employing **2GtBuCzCO_2_HDCzB** as the emitter, at 1000 cd m^−2^, is one of the best among the green SP‐OLEDs, and the best amongst the green solution‐processed MR‐TADF OLEDs (Figure 7d, Supporting Information). Despite both emitters having only moderate *k*_RISC_, the enhanced efficiency and suppressed efficiency roll‐off of the doped and non‐doped devices with **2GtBuCzCO_2_HDCzB** can be explained by the encapsulating potential of the donor dendron, which effectively suppresses intermolecular exciton quenching. The contrasting device performance between those with **2GtBuCzCO_2_HDCzB** and with **tBuCzCO_2_HDCzB** underscores the significance of the emitter design that leverages the encapsulation of the MR‐TADF core with peripheral TADF pendant moieties.

## 3 Conclusion

By adopting an emitter design that thoroughly encapsulates the MR‐TADF emissive core **DtBuCzB** within flanking donor‐acceptor TADF groups, both **2GtBuCzCO_2_HDCzB** and **tBuCzCO_2_HDCzB** showed bright green emission that was resistant to aggregation and aggregation‐caused quenching, even as neat films. We demonstrated through a combined computational and photophysical study how the donor‐acceptor TADF groups act to harvest excitons and then efficiently transfer their energy by FRET to the MR‐TADF core where emission occurs. The use of TADF dendrimers in **2GtBuCzCO_2_HDCzB** results in improved suppression of concentration quenching. The SP‐OLEDs with **2GtBuCzCO_2_HDCzB** in 30 wt% mCP emitted narrowband green emission at λ_EL_ of 495 nm and FWHM of 27 nm and showed both a high EQE_max_ of 27.9% and a small efficiency roll‐off with EQE_5000_ of 22.3%. Though the photophysics of **tBuCzCO_2_HDCzB** was similar to that of **2GtBuCzCO_2_HDCzB** the EQE_max_ of the device with **tBuCzCO_2_HDCzB** was only 22.0% and the efficiency roll‐off was larger where the EQE_5000_ was 16.3%. The EQE_max_ of the host‐free SP‐OLEDs with **2GtBuCzCO_2_HDCzB** remained high at 24.0% and the efficiency roll‐off was low, with EQE_1000_ of 20.2%, which is comparable to our previously reported **tBuCz2m2pTRZ** dendrimer OLED (EQE_max_  =  28.7%), especially at high brightness (EQE_500_ of 22.7%). These results demonstrate the value of this emitter design to translate into high‐performance SP‐OLEDs.

## Conflict of Interest

The authors declare no conflict of interest.

## Supporting information



Supporting Information

## Data Availability

The research data supporting this publication can be accessed at https://doi.org/10.17630/625c42fc‐7aa3‐44f5‐9a9a‐7cd6fffe54f0.
