# Supplementary material for: A Multiresonant Thermally Activated Delayed Fluorescent Dendrimer with Intramolecular Energy Transfer: Application for Efficient Host‐Free Green Solution‐Processed Organic Light‐emitting Diodes
Source: Adv Mater. 2025 Jan 9;37(8):2415289. doi: 10.1002/adma.202415289 (PMC11854867; doi:10.1002/adma.202415289)
Supplement: Supplementary file 1 — Supporting Information [file ADMA-37-2415289-s001.pdf]

ADVANCED MATERIALS

Supporting Information

for *Adv. Mater.*, DOI 10.1002/adma.202415289

A Multiresonant Thermally Activated Delayed Fluorescent Dendrimer with Intramolecular Energy Transfer: Application for Efficient Host-Free Green Solution-Processed Organic Light-emitting Diodes

Sen Wu, Dongyang Chen, Xiao-Hong Zhang, Dianming Sun* and Eli Zysman-Colman**

A Multiresonant Thermally Activated Delayed Fluorescent Dendrimer Benefiting from Intramolecular Energy Transfer: Application for Efficient Host-Free Green Solution-Processed Organic Light-emitting Diodes

Sen Wu[+,a] *Dongyang Chen*[+,a,b] *Xiao-Hong Zhang,*[*b,c] *Dianming Sun,*[*a] *and Eli Zysman-Colman*[*a]

[a]Organic Semiconductor Centre, EaStCHEM School of Chemistry, University of St Andrews, St Andrews, Fife, UK, KY16 9ST, Fax: +44-1334 463808; Tel: +44-1334 463826; E-mail: eli.zysman-colman@st-andrews.ac.uk; sd235@st-andrews.ac.uk.

[b]Institute of Functional Nano & Soft Materials (FUNSOM), Joint International Research Laboratory of Carbon-Based Functional Materials and Devices, Soochow University, Suzhou, Jiangsu 215123, P. R. China.

[c]Jiangsu Key Laboratory of Advanced Negative Carbon Technologies, Soochow University, Suzhou, 215123, Jiangsu, P. R. China.

[+]Both authors contributed equally to this work.

Table of Contents

General methods **S3**

Literature examples **S7**

Compounds Characterization **S8**

Computations **S19**

Photophysical characterization **S22**

Devices **S33**

References **S37**

General methods

General Synthetic Procedures. DCzB-Bpin was synthesised according to the reported procedure.[1] The other reagents and solvents were obtained from commercial sources and used as received unless otherwise stated. Air-sensitive reactions were done under a nitrogen atmosphere using Schlenk techniques. Dry solvents used in the reaction were obtained from a MBRAUN SPS5 solvent purification system. Flash column chromatography was carried out using silica gel (Silia-P from Silicycle, 60 Å, 40-63 μm). Analytical thin-layer-chromatography (TLC) was performed with silica plates with aluminium backings (250 μm with F-254 indicator). TLC visualization was accomplished by 254/365 nm UV lamp. HPLC was conducted on a Shimadzu LC-40 HPLC system. HPLC traces were performed using a Shim-pack GIST 3μm C18 reverse phase analytical column. ^{1}H and ^{13}C and NMR spectra were recorded on a Bruker Advance spectrometer (400 MHz for ^{1}H and 126 MHz for ^{13}C). The following abbreviations have been used for multiplicity assignments: "s" for singlet, "d" for doublet, "t" for triplet, "m" for multiplet, "dd" for doublet of doublets, "dt" for doublet of triplets. ^{1}H and ^{13}C NMR spectra were referenced to the solvent peaks). Melting points were measured using open-ended capillaries on an Electrothermal 1101D Mel-Temp apparatus and are uncorrected. High-resolution mass spectrometry (HRMS) was performed at University of Edinburgh Mass Spectrometry Facility. Elemental analyses were performed by Dr. Joe Casillo at the University of Edinburgh.

Quantum chemical calculations. The calculation was performed using Density Functional Theory (DFT) with Gaussian 16[2] as well as the second order algebraic diagrammatic construction Spin-Component Scaling (ADC(2)-SCS) method[3] using the Turbomole/7.5 package.[4] For the DFT calculation, the ground state, excited triplet states were optimized with the M06-2X functional[5] and the 6-31G(d,p) basis set[6] and the excited-state calculations were performed at Time-dependent DFT (TD-DFT)[7] using the same functional and basis set as for ground state geometry optimization. For the ADC(2) calculation, the ground states was optimized with ADC(2)-SCS method and cc-pVDZ basis set.[8] Vertical excited states were performed on the ground state optimized structure using ADC(2)-SCS method. All the calculations were submitted, and all calculations were submitted using the Silico v4 software package.[9–12]

Electrochemistry measurements. Cyclic Voltammetry (CV) analysis was performed on an Electrochemical Analyzer potentiostat model 620E from CH Instruments at a sweep rate of 100 mV/s. Differential pulse voltammetry (DPV) was conducted with an increment potential of 0.004 V and a pulse amplitude, width, and period of 50 mV, 0.05, and 0.5 s, respectively.

Samples were prepared in DCM solutions, which were degassed by sparging with DCM-saturated nitrogen gas for 5 minutes prior to measurements. All measurements were performed using 0.1 M DCM solution of tetra-*n*-butylammonium hexafluorophosphate, [$^{n}Bu_4N]PF_6$. An Ag/Ag^+ electrode was used as the reference electrode while a platinum electrode and a platinum wire were used as the working electrode and counter electrode, respectively. The redox potentials are reported relative to a saturated calomel electrode (SCE) with a ferrocenium/ferrocene (Fc/Fc^+) redox couple as the internal standard (0.45 V vs SCE).[13] The HOMO and LUMO energies were determined using the relation $E_{HOMO}/_{LUMO} = -(E_{ox} / E_{red} + 4.8)$ eV, where E_{ox} and E_{red} are the onset of anodic and cathodic peak potentials, respectively calculated from DPV relative to Fc/Fc^+.[14]

Photophysical measurements. Optically dilute solutions of concentrations on the order of 10^{-5} or 10^{-6} M were prepared in spectroscopic grade solvents for absorption and emission analysis. Absorption spectra were recorded at room temperature on a Shimadzu UV-2600 double beam spectrophotometer with a 1 cm quartz cuvette. Molar absorptivity determination was verified by linear regression analysis of values obtained from at least four independent solutions at varying concentrations range from 3.0×10^{-6} to 1.0×10^{-5} with absorbance ranging from 0.05 to 0.30. For emission studies, steady-state emission and time-resolved emission spectra were recorded at room temperature using an Edinburgh Instruments FS5 fluorimeter. Samples were excited at 340 nm for steady-state measurements and 375 nm for time-resolved PL decays. Photoluminescence quantum yields for solutions were determined using the optically dilute method, in which four sample solutions with absorbances of ca. 0.10, 0.075, 0.050 and 0.025 at 340 nm was used.[15] The Beer-Lambert law was found to remain linear at the concentrations of the solutions. For each sample, linearity between absorption and emission intensity was verified through linear regression analysis with the Pearson regression factor (R^2) for the linear fit of the data set surpassing 0.9. Individual relative quantum yield values were calculated for each solution and the values reported represent the slope obtained from the linear fit of these results. The quantum yield of the sample, Φ_{PL}, was determined using the equation $\Phi_{PL} = (\Phi_r * \frac{A_r}{A_s} * \frac{I_s}{I_r} * \frac{n_s^2}{n_r^2})$,[15] where A stands for the absorbance at the excitation wavelength (λ_{exc} = 340 nm), I is the integrated area under the corrected emission curve and n is the refractive index of the solvent with the subscripts “s” and “r” representing sample and reference respectively. Φ_r is the absolute quantum yield of the external reference quinine sulfate (Φ_r = 54.6% in 1 *N* H_2SO_4).[16]

An integrating sphere (Edinburgh Instruments FS5, SC30 module) was employed for the photoluminescence quantum yield measurements of thin film samples. The Φ_{PL} of the films were then measured in air and in N_2 by purging the integrating sphere with N_2 gas flow for 2

min. The photophysical properties of the film samples were measured using an Edinburgh Instruments FS5 fluorimeter. Time-resolved PL measurements of the thin films were carried out using the multi-channel scaling (MCS) and time-correlated single-photon counting (TCSPC) technique. The samples were excited at 379 nm by a pulsed laser and were kept in a vacuum of $< 8 \times 10^{-4}$ mbar. The singlet and triplet state energies in 2-MeTHF glass and in doped film were determined from the onset values of the steady-state photoluminescence (SS PL) and time-gated (1-10 ms) phosphorescence spectra at 77 K. The singlet-triplet energy gap (ΔE_{ST}) was estimated from the difference in energy of the steady-state PL and phosphorescence spectra. The samples were excited by a xenon flashlamp emitting at 340 nm (EI FS5, SC-70).

Fitting of time-resolved luminescence measurements: Time-resolved PL measurements were fitted to a sum of exponentials decay model, with chi-squared (χ^2) values between 1 and 2, using the EI FS5 Each component of the decay is assigned with a weight, (w_i), which is the contribution of the emission from each component to the total emission.

The average lifetime was then calculated using the following expressions:

1. Two exponential decay model:

$$\tau_{AVG} = \tau_1 w_1 + \tau_2 w_2 \qquad \text{(S1)}$$

with weights defined as $w_1 = \frac{A1\tau_1}{A1\tau_1 + A2\tau_2}$ and $w_2 = \frac{A2\tau_2}{A1\tau_1 + A2\tau_2}$ where A_1 and A_2 are the preexponential-factors of each component.

2. Three exponential decay model:

$$\tau_{AVG} = \tau_1 w_1 + \tau_2 w_2 + \tau_3 w_3 \qquad \text{(S2)}$$

with weights defined as $w_1 = \frac{A1\tau_1}{A1\tau_1 + A2\tau_2 + A3\tau_3}$, $w_2 = \frac{A2\tau_2}{A1\tau_1 + A2\tau_2 + A3\tau_3}$ and $w_3 = \frac{A3\tau_3}{A1\tau_1 + A2\tau_2 + A3\tau_3}$ where A_1, A_2 and A_3 are the preexponential-factors of each component.

OLED Fabrication and Characterization: The OLED devices were fabricated in a bottom-mitting architecture on indium-doped tin oxide (ITO, 12 mm × 12 mm × 1.1 mm, resistivity: 15 Ω sq^{-1}) substrates. A pre-patterned glass substrate coated with ITO were washed sequentially by ultrasonication in acetone, and isopropanol for 20 min and then exposed to oxygen plasma for 10 min to remove all the dust and organics on the ITO surface and to increase the work function of ITO anode for better hole injection from the anode to organic layer. The hole injection layer poly(3,4-ethylenedioxythiophene):poly(styrenesulfonate) (PEDOT:PSS; Heraeus; Clevios P VP AI 4083; charge: 9001157883) were spin-coated onto the ITO surface under 4000 rpm for 1 min, and baked at 130 °C for 15 min to remove the residual water, then

transferred all substrates inside a nitrogen-filled glovebox. The emitting layer was spin-coated under 2000 rpm, and then annealed at 100 °C in the nitrogen-filled glovebox. The electron transporting layer (3,3′-(5′-(3-(pyridin-3-yl)phenyl)-[1,1′:3′,1″-terphenyl]-3,3″-diyl)dipyridine) (TmPyPB), the electron injecting layer (lithium fluoride, LiF 1 nm), and the aluminum cathode (100 nm) were successively thermally evaporated in a vacuum chamber under <1 × 10^{-6} mbar. After the evaporation, the OLEDs were taken out from the evaporator and encapsulated inside the glovebox. Then, the devices were taken out from the glovebox for current−voltage−luminance characteristics. The luminance-current-voltage characteristics were measured in an ambient environment using IV Curve Measurement System manufactured by Ossila. The electroluminescence spectra were recorded by an Andor DV420-BV CCD spectrometer.

Literature examples

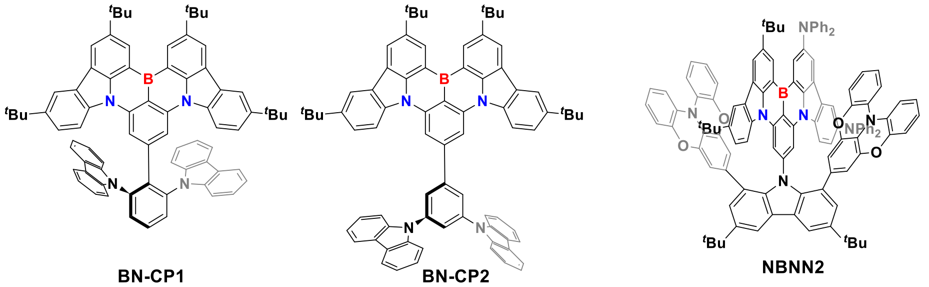

BN-CP1 **BN-CP2**

Adv. Mater. **2021,** 2106954

NBNN2

Angew. Chem.Int. Ed. **2023**, *62*, 202310943

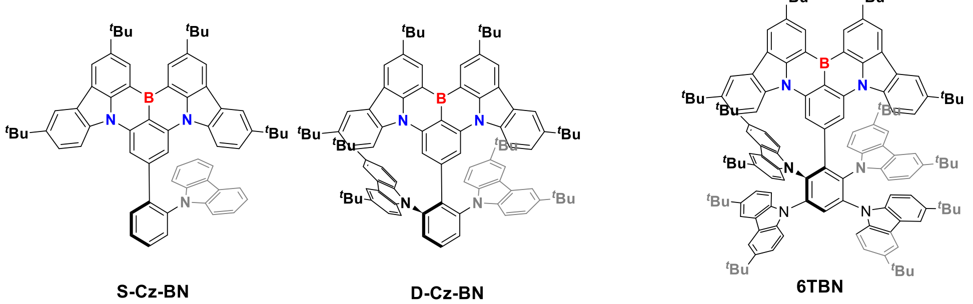

S-Cz-BN **D-Cz-BN**

Angew. Chem. Int. Ed. **2022,** *61*, 202113206

6TBN

ACS Appl. Mater. Interfaces **2023**, *15*, 39669

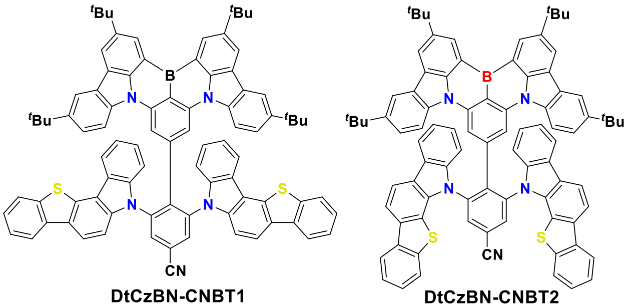

DtCzBN-CNBT1 **DtCzBN-CNBT2**

ChemRxiv, 2024, DOI: 10.26434/chemrxiv-2024-g4blr

Figure **S1**. Molecular structures of encapsulated MR-TADF emitters discussed in the main text.

Experimental section

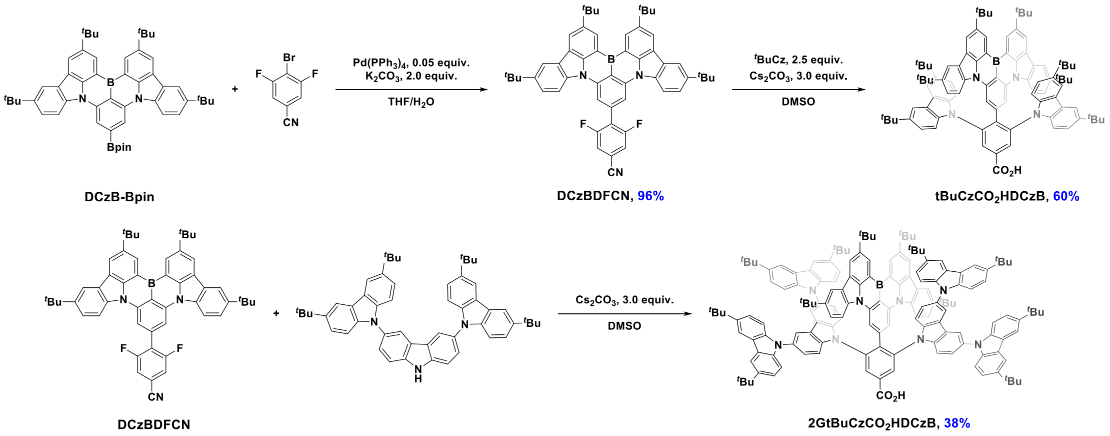

Scheme **S1**. Synthesis route of **tBuCzCO₂HDCzB** and **2GtBuCzCO₂HDCzB**.

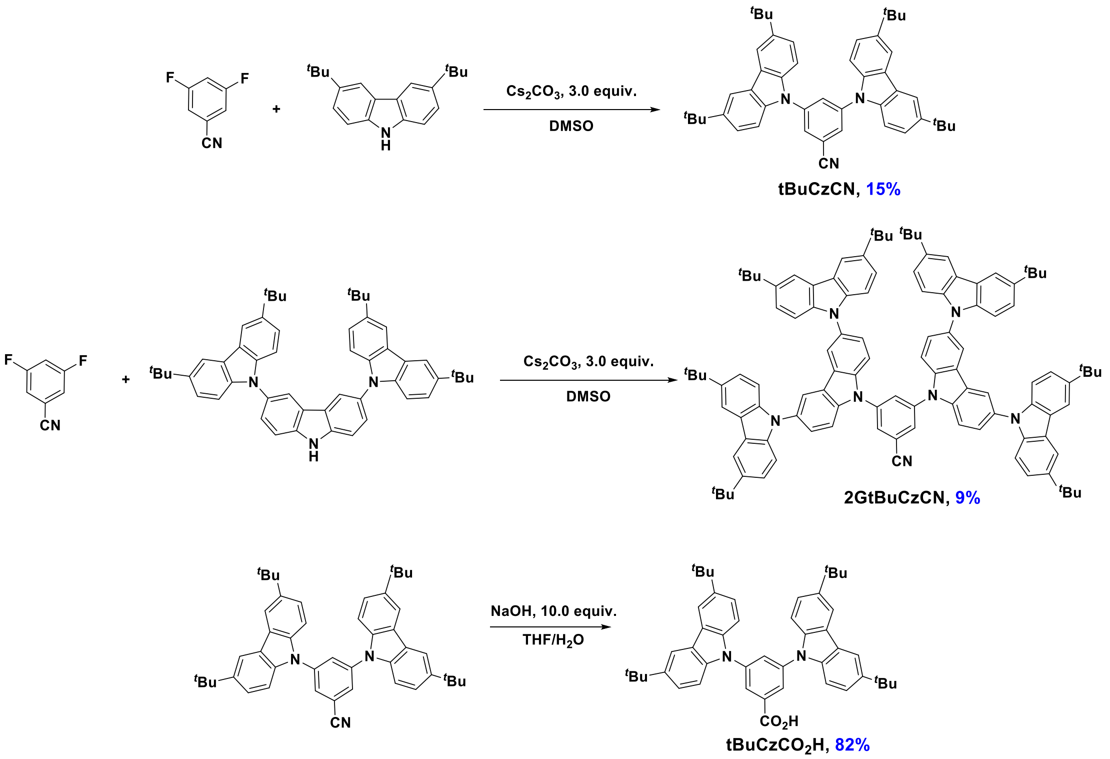

Scheme **S2**. Synthesis route of **tBuCzCN**, **tBuCzCO₂H** and **2GtBuCzCN**.

Synthesis of DCzBDFCN

Under a nitrogen atmosphere, to **DCzB-Bpin** (400 mg, 0.52 mmol 1 equiv.) were added 4-bromo-3,5-difluorobenzonitrile (171 mg, 0.78 mmol, 1.5 equiv.), THF (15 mL) and $NaCO_{3aq}$ (2 M, 0.5 mL). Under a positive flow of nitrogen, $Pd(PPh_3)_4$ (30 mg, 0.026 mmol, 0.05 equiv.) was added and the solution was then heated at reflux for 12 h. The reaction was cooled to room temperature and diluted with DCM (150 mL). The organic layer was washed with water (3 × 50 mL) and then dried with anhydrous sodium sulfate. The solvents were removed under reduced pressure. The crude product was purified by column chromatography on silica gel

(DCM:hexane = 1:3). The corresponding fractions were combined and concentrated under reduced pressure to afford bright yellow solid. **Yield:** 96% (390 mg) **R_f:** 0.50 (DCM:hexane = 1:2) **Mp:** 341-342 °C. **^{1}H NMR (500 MHz, $CDCl_3$) δ (ppm):** 9.14 (s, 2H), 8.46 (d, J = 48.5 Hz, 4H), 8.30 (d, J = 9.7 Hz, 4H), 7.68 (d, J = 8.7 Hz, 2H), 7.50 (d, J = 6.4 Hz, 2H), 1.71 (s, 18H), 1.56 (s, 18H). **^{13}C NMR (126 MHz, CDCl3) δ (ppm):** 161.23, 159.28, 145.69, 145.02, 144.06, 141.63, 138.15, 129.88, 127.14, 124.61, 123.80, 121.59, 121.07, 117.43, 116.55, 116.31, 113.83, 112.98, 109.40, 35.23, 34.85, 32.19, 31.83. **HRMS-ESI (m/z)**: $[M+H]^+$ Calculated for **$C_{53}H_{50}F_2N_3$:** 778.4147; **Found:** 778.4131.

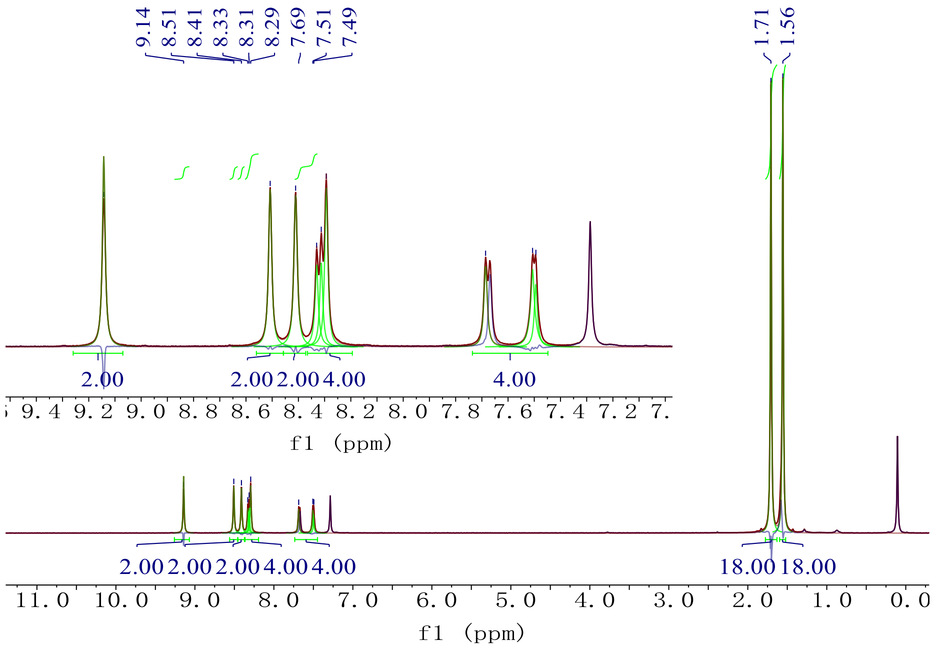

Figure **S2**. ^{1}H-NMR spectrum of **DCzBDFCN** in $CDCl_3$.

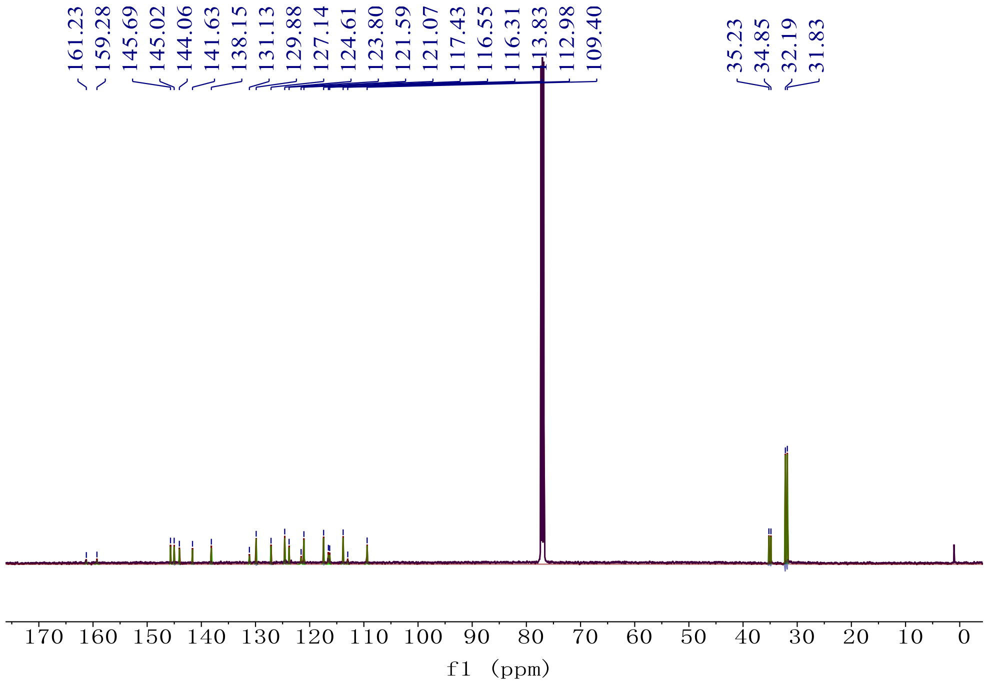

Figure **S3**. ^{13}C-NMR spectrum of **DCzBDFCN** in $CDCl_3$.

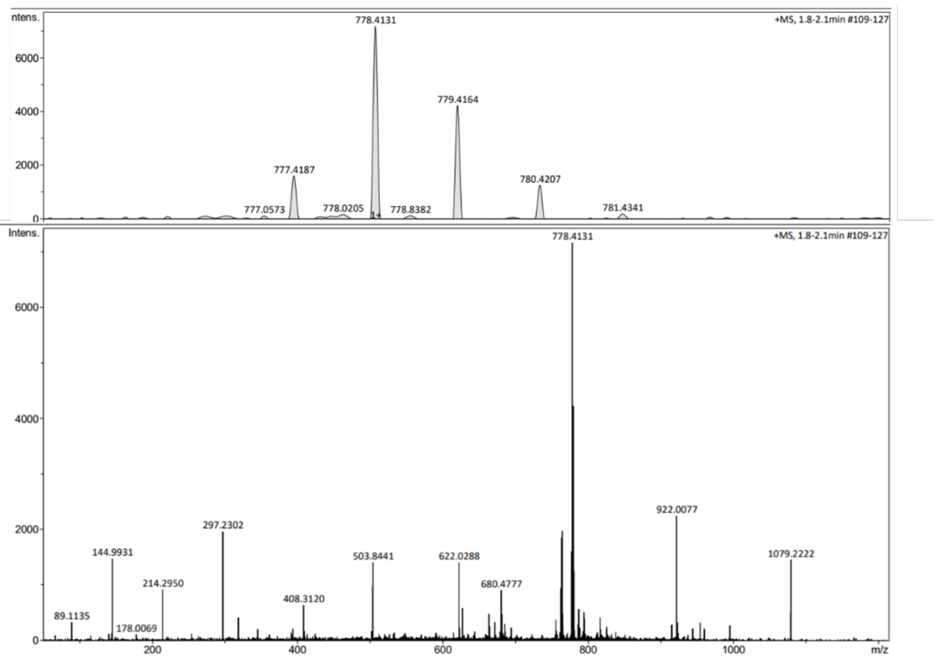

Figure **S4**. ESI-HRMS spectrum of **DCzBDFCN**.

Synthesis of 3,5-bis(3,6-di-tert-butyl-9H-carbazol-9-yl)benzonitrile (tBuCzCN)

Under nitrogen, a mixture of 3,6-di-*tert*-butyl-9H-carbazole (606 mg, 2.17 mmol, 2.01 equiv.), 3,5-difluorobenzonitrile (150 mg, 1.08 mmol, 1 equiv.) and cesium carbonate (878 mg, 2.70 mmol, 2.5 equiv.) in dry DMF (20 ml) were refluxed for 24 h. After cooling to room temperature, the reaction was extracted with chloroform and washed with water (3 × 30 mL). The organic

phase was dried over Na_2SO_4 and concentrated under reduced pressure. The crude product was purified by column chromatography on silica gel using 1:5 DCM/hexane as eluent to afford a white solid. **Yield:** 15% (105 mg) **R_f:** 0.40 (DCM:hexane = 1:5) **Mp:** 363-365 °C. **^{1}H NMR (500 MHz, $CDCl_3$) δ (ppm):** 8.23 – 8.19 (m, 4H), 8.14 (t, J = 2.0 Hz, 1H), 7.99 (d, J = 2.0 Hz, 2H), 7.56 (d, J = 1.9 Hz, 4H), 7.53 – 7.49 (m, 4H), 1.52 (s, 36H). **^{13}C NMR (126 MHz, $CDCl_3$) δ (ppm):** 144.28, 141.13, 138.32, 128.28, 127.34, 124.22, 124.09, 117.60 116.72, 115.41, 108.87, 34.87, 31.99. **HRMS-ESI (m/z)**: $[M+H]^+$ Calculated for **$C_{47}H_{51}N_3$:** 657.4078; **Found:** 657.4097.

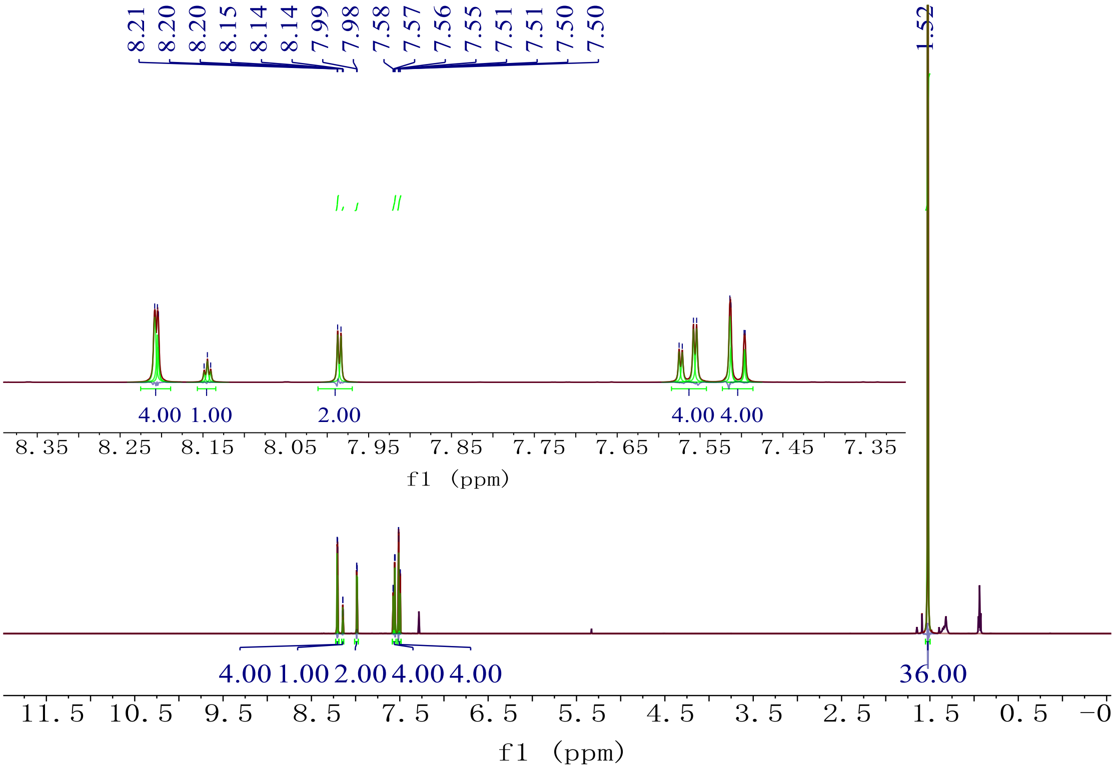

Figure **S5**. ^{1}H-NMR spectrum of **tBuCzCN** in $CDCl_3$.

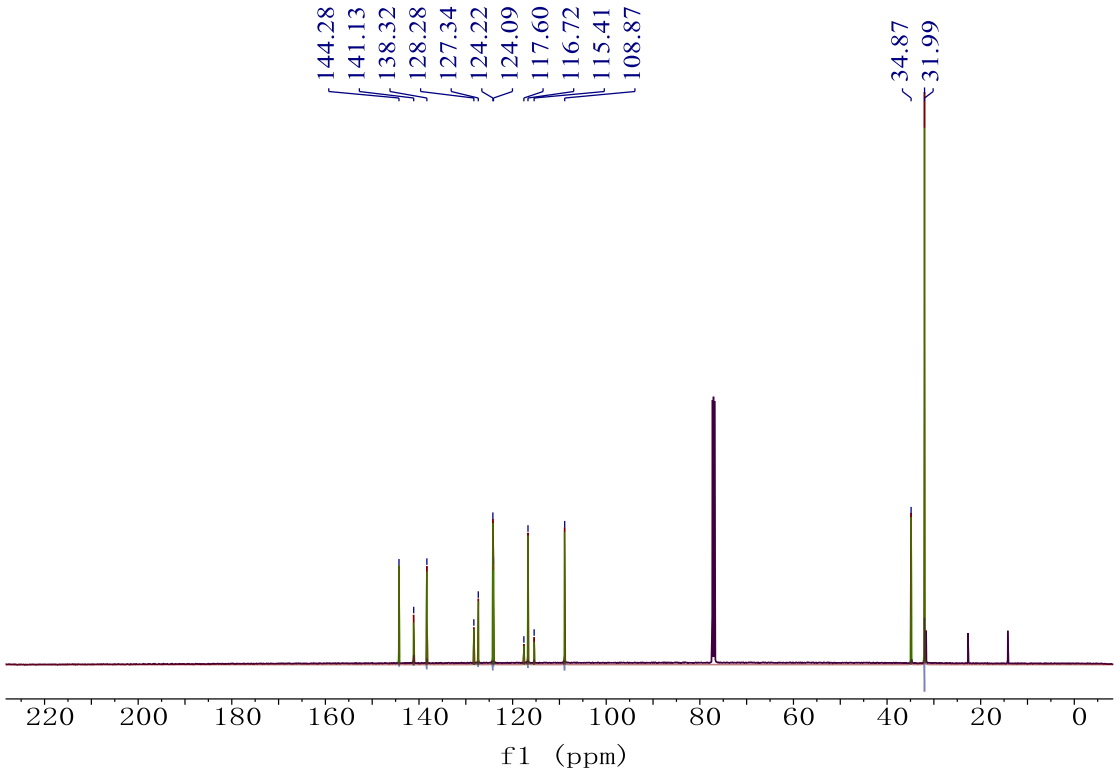

Figure **S6**. ^{13}C-NMR spectrum of **tBuCzCN** in $CDCl_3$.

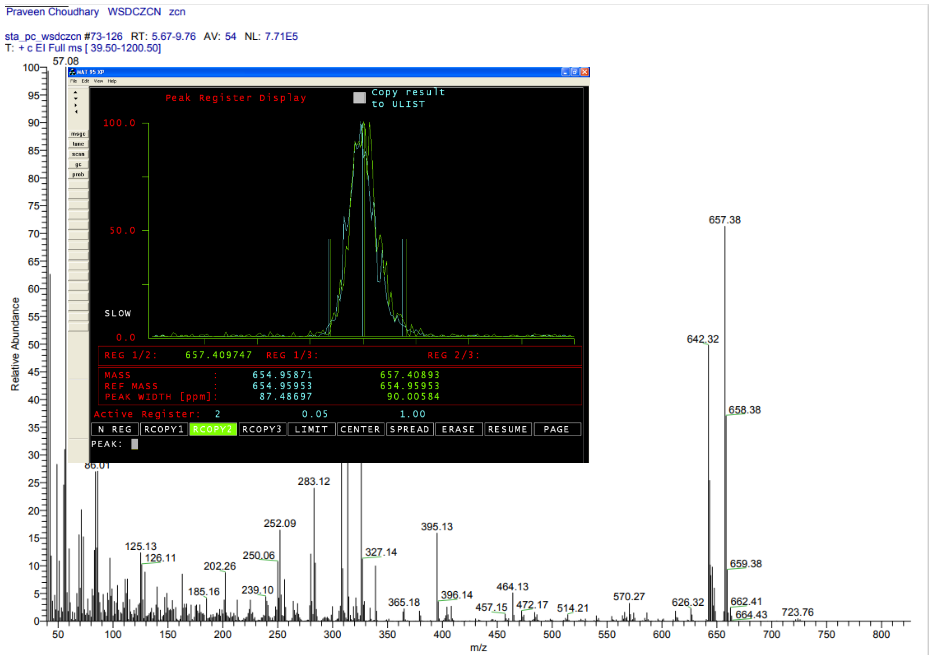

Figure **S7**. ESI-HRMS spectrum of **tBuCzCN**.

Synthesis of 3,5-bis(3,6-di-tert-butyl-9H-carbazol-9-yl)benzoic acid (tBuCzCO$_2$H)

tBuCzCN (100 mg, 0.15 mmol, 1 equiv.) was combined with sodium hydroxide (61 mg, 1.52 mmol, 10 equiv.) in 10 mL of an THF/water (1:1) mixture. The reaction was heated to reflux for 12 h. After cooling to room temperature, the pH was adjusted to 2-3 by addition of dilute

hydrochloric acid. The diacid precipitated as a light green solid and was collected by vacuum filtration and washed thoroughly with water. The product was recrystalized from methanol/THF to obtain a white powder. **Yield:** 82% (85 mg) **Mp**: 335-338 °C. **^{1}H NMR (500 MHz, CD_2Cl_2)** δ 8.44 (d, *J* = 2.0 Hz, 2H), 8.23 (t, *J* = 1.3 Hz, 4H), 8.15 (t, *J* = 2.0 Hz, 1H), 7.56 (d, *J* = 1.5 Hz, 8H), 1.50 (s, 36H). **^{13}C NMR (126 MHz, CD_2Cl_2)** δ 169.65, 143.75, 140.25, 138.71, 125.98, 124.02, 123.74, 116.50, 109.04, 34.67, 31.69. **HRMS-ESI (m/z)**: $[M+H]^+$ Calculated for **$C_{47}H_{52}N_2O_2$:** 677.4102; **Found:** 677.4103.

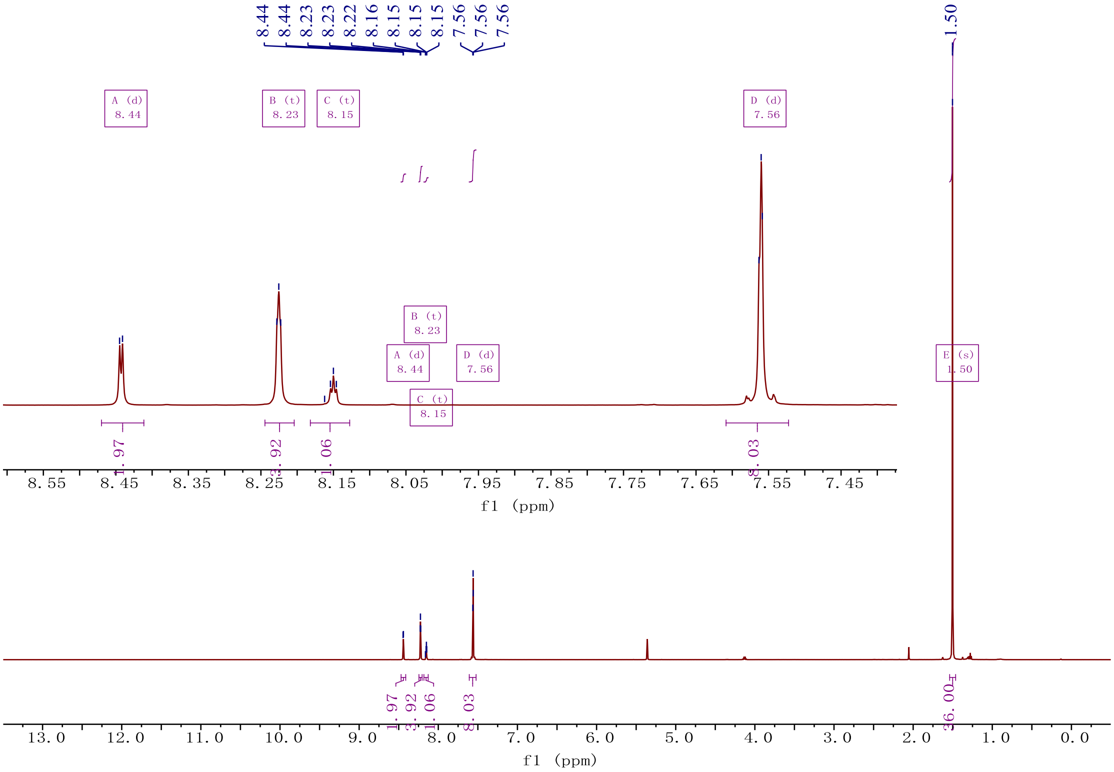

Figure **S8**. ^{1}H-NMR spectrum of **$tBuCzCO_2H$** in CD_2Cl_2.

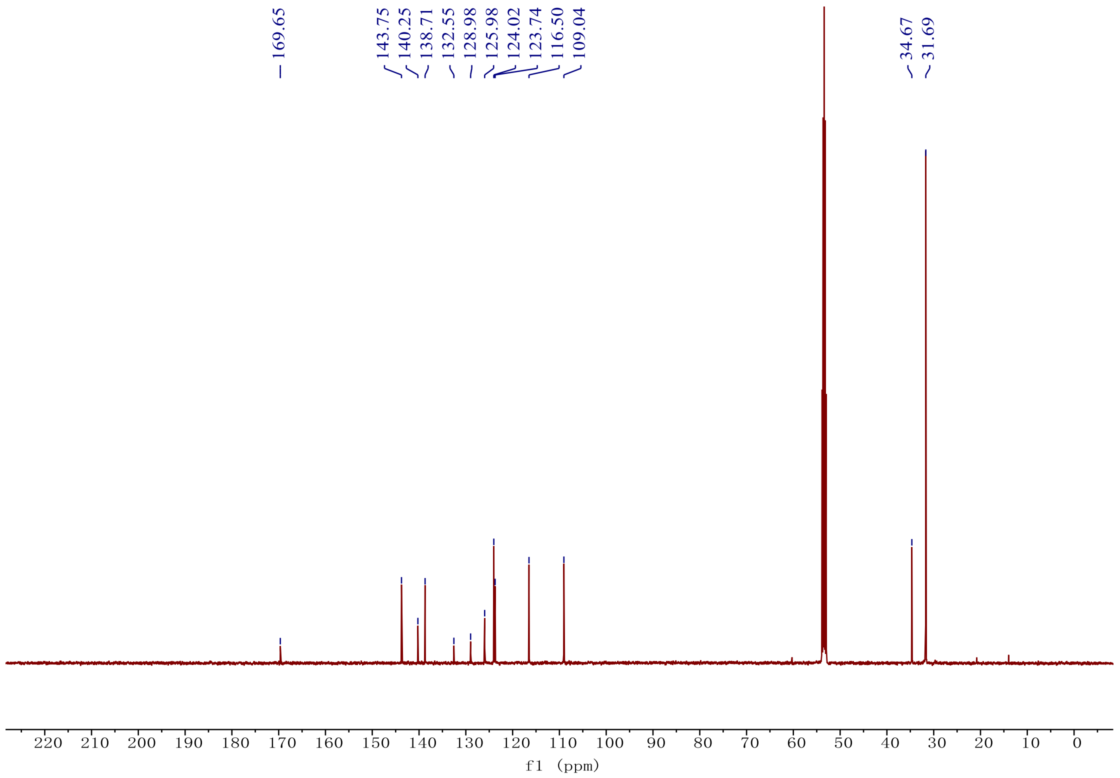

Figure **S9**. ^{13}C-NMR spectrum of **tBuCzCO$_2$H** in CD_2Cl_2.

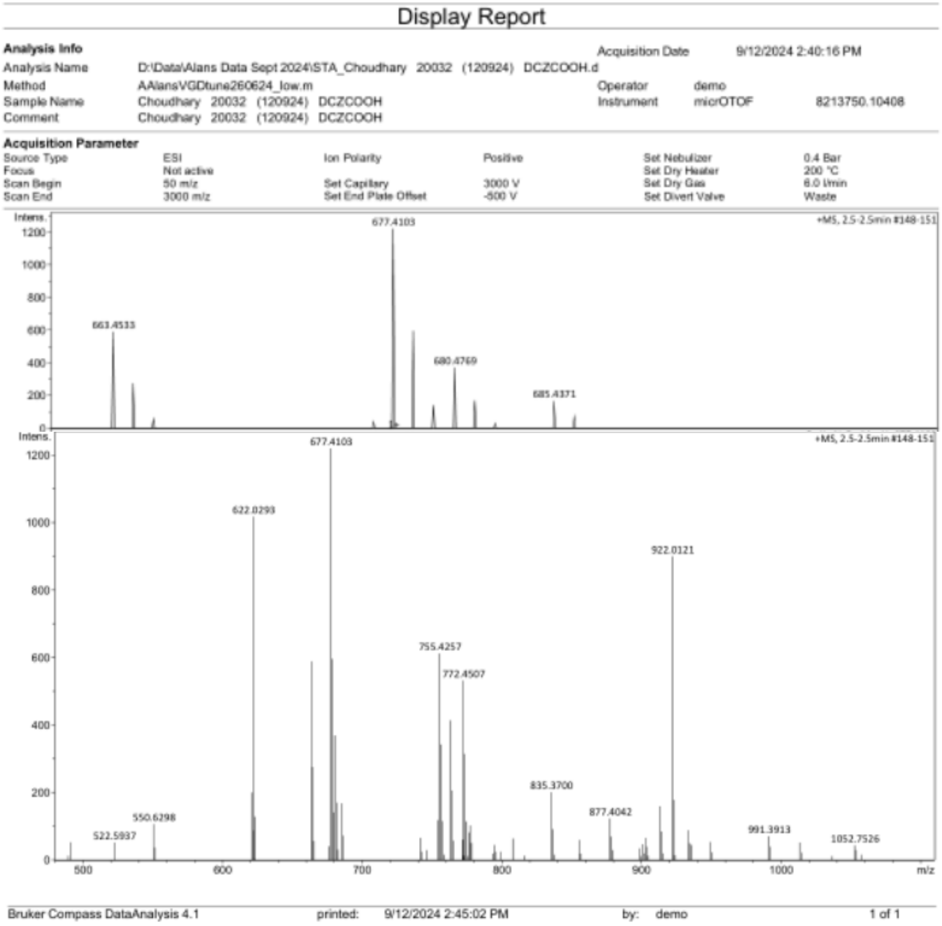

Figure **S10**. ESI-HRMS of **tBuCzCO$_2$H.**

Synthesis of 2GtBuCzCN

A similar procedure to **2GtBuCzCN** was employed but using 3,5-difluorobenzonitrile and 3,3'',6,6''-tetrakis(*tert*-butyl-9'H-9,3':6',9''-tercarbazole). **Yield:** 9% (50 mg) **R$_f$:** 0.40 (DCM:Hexane = 1:5). **Mp**: 325-327 °C. **^{1}H NMR (500 MHz, Acetone-*d$_6$*) δ (ppm):** δ 8.84 – 8.75 (m, 1H), 8.72 – 8.53 (m, 6H), 8.30 (d, *J* = 2.3 Hz, 8H), 8.11 (td, *J* = 8.5, 4.0 Hz, 4H), 7.79 (ddd, *J* = 8.0, 5.6, 2.3 Hz, 4H), 7.51 – 7.40 (m, 8H), 7.36 (ddd, *J* = 8.5, 5.4, 2.7 Hz, 8H), 1.43 (s, 72H). **^{13}C NMR (126 MHz, Acetone-*d$_6$*) δ (ppm):** 142.47, 140.27, 139.97, 131.66, 130.44, 130.01, 126.22, 124.74, 123.57, 123.25, 119.81, 117.33, 116.36, 111.53, 109.14, 34.38, 31.46. **HRMS-ESI (m/z)**: $[M]^+$ Calculated for **$C_{111}H_{111}N_7$:** 1541.8901; **Found:** 1541.9106.

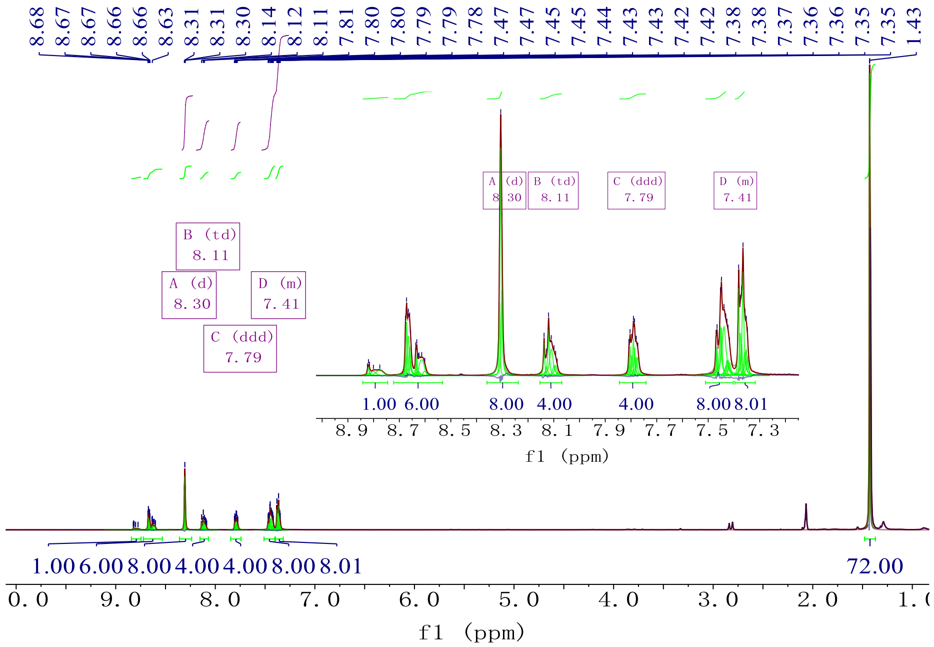

Figure **S11**. ^{1}H-NMR spectrum of **2GtBuCzCN** in Acetone-d_6.

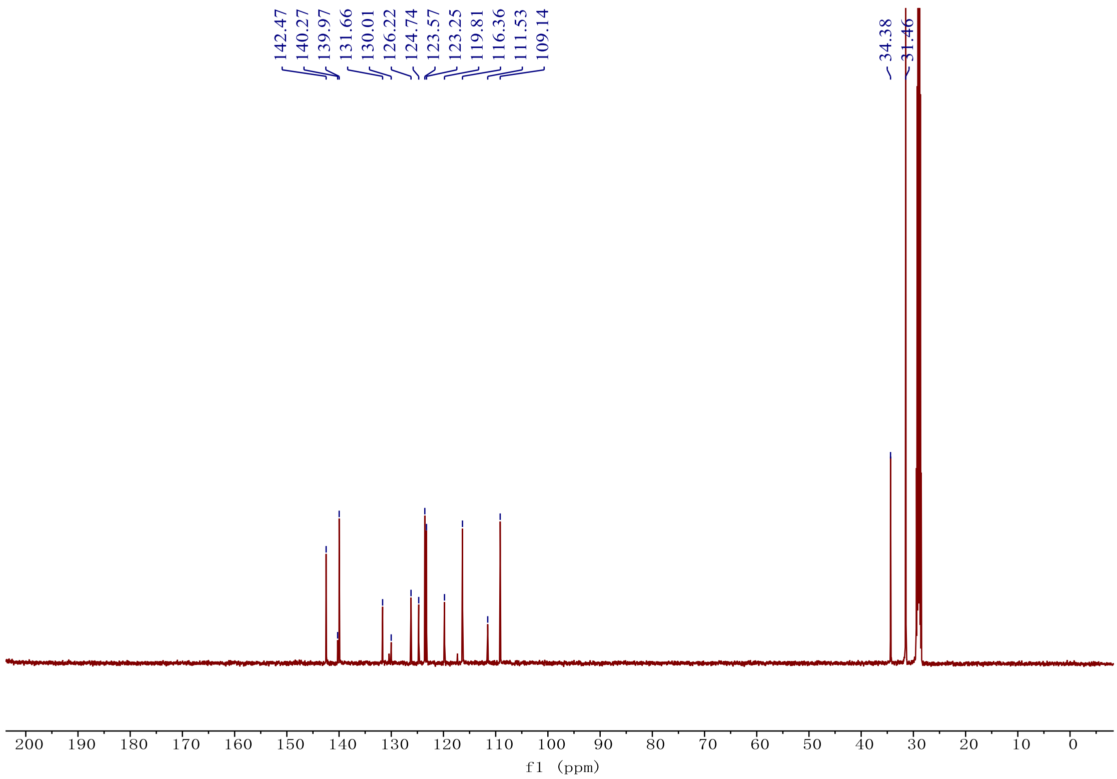

Figure **S12**. ^{13}C-NMR spectrum of **2GtBuCzCN** in Acetone-d_6.

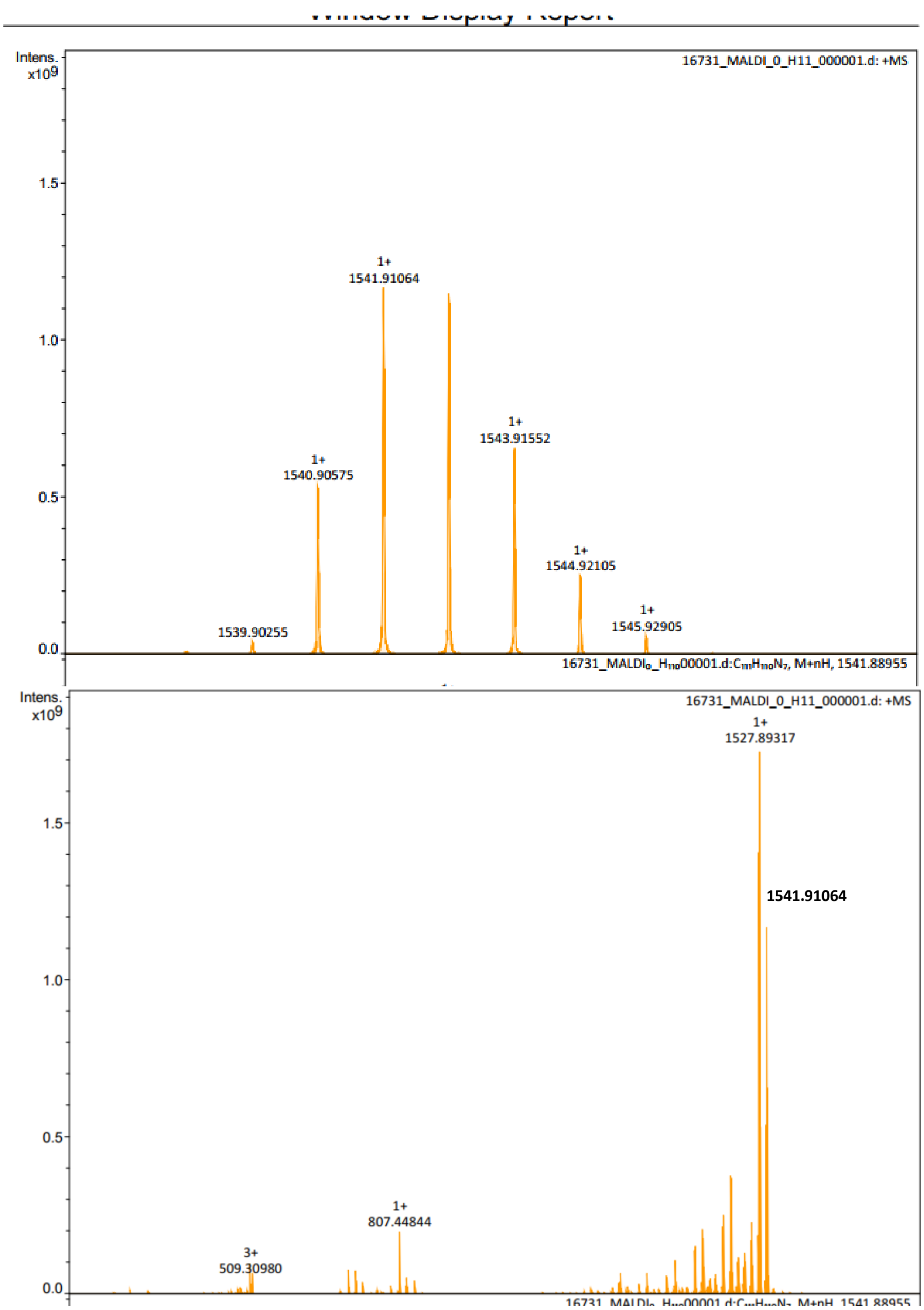

Figure **S13**. MALDI-MS spectrum of **2GtBuCzCN**.

Synthesis of tBuCzCO$_2$HDCzB.

A similar procedure to **tBuCzCNDCzB** was employed but using **DCzBDFCN** and 3,6-di-*tert*-butyl-9H-carbazole and DMSO as solvent. Under these conditions, the nitrile was hydrolyzed to the acid. **Yield:** 60% (250 mg) **R$_f$:** 0.40 (DCM:hexane = 2:5) **Mp**: 302-303 °C. **^{1}H NMR (500 MHz, CD$_2$Cl$_2$) δ (ppm):** 8.89 (d, *J* = 1.9 Hz, 2H), 8.39 (d, *J* = 1.8 Hz, 2H), 8.28 (s, 2H), 8.19 (d, *J* = 2.1 Hz, 2H), 7.90 – 7.83 (m, 6H), 7.49 – 7.42 (m, 6H), 7.39 (dt, *J* = 8.6, 1.9 Hz,

6H), 1.61 (s, 18H), 1.56 (s, 18H), 1.20 (s, 36H). **^{13}C NMR (126 MHz, CD_2Cl_2) δ (ppm):** 166.61, 145.56, 145.02, 144.59, 143.04, 142.81, 140.33, 139.38, 138.38, 137.71, 135.97, 129.47, 129.36, 126.66, 124.01, 123.49, 123.31, 120.47, 117.13, 116.47, 113.83, 109.44, 108.40, 34.95, 34.62, 34.37, 31.81, 31.63, 31.52. **HRMS-ESI (m/z)**: $[M]^+$ Calculated for **$C_{93}H_{99}BN_4O_2$:** 1314.7861; **Found:** 1314.8097. **Elemental analysis**: Calcd. for **$C_{93}H_{98}BN_5$**: C, 84.90%, H, 7.58%, and N, 4.26%. Anal. C, 84.37%, H, 6.95%, and N, 4.64%. **HPLC analysis**, 99.36% pure, retention time 6.372 min in 80% THF, 20% water.

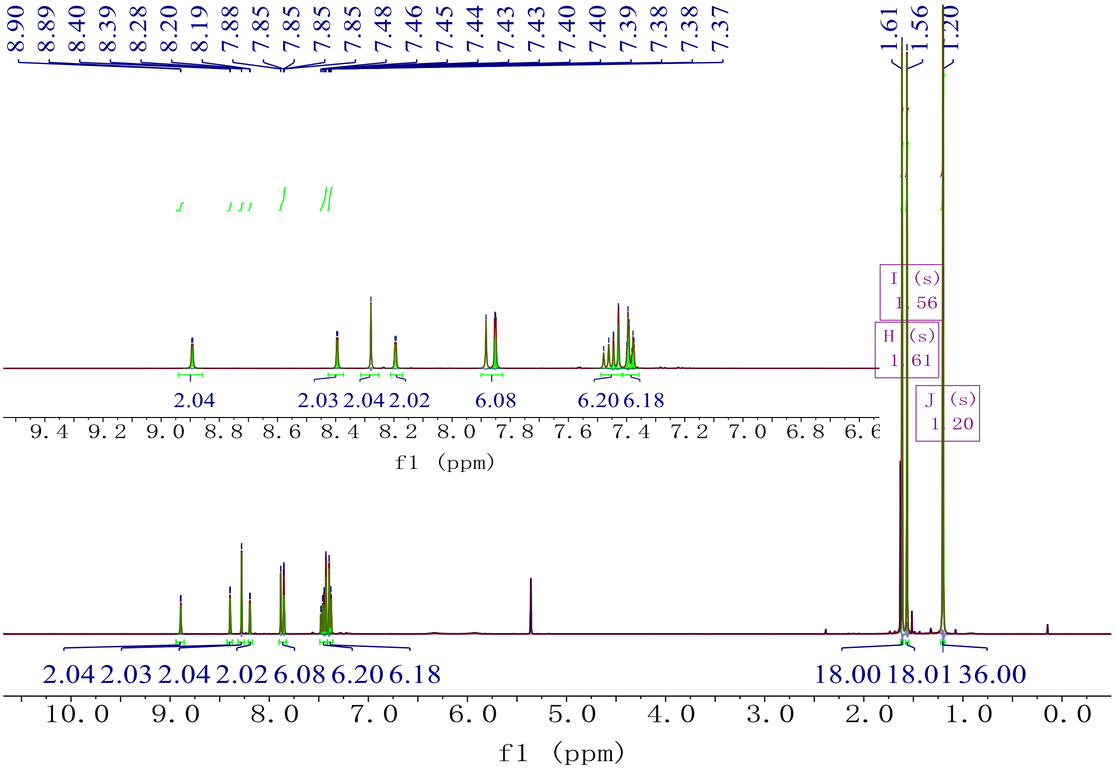

Figure **S14**. **^{1}H-NMR** spectrum of **tBuCzCO$_2$HDCzB** in CD_2Cl_2.

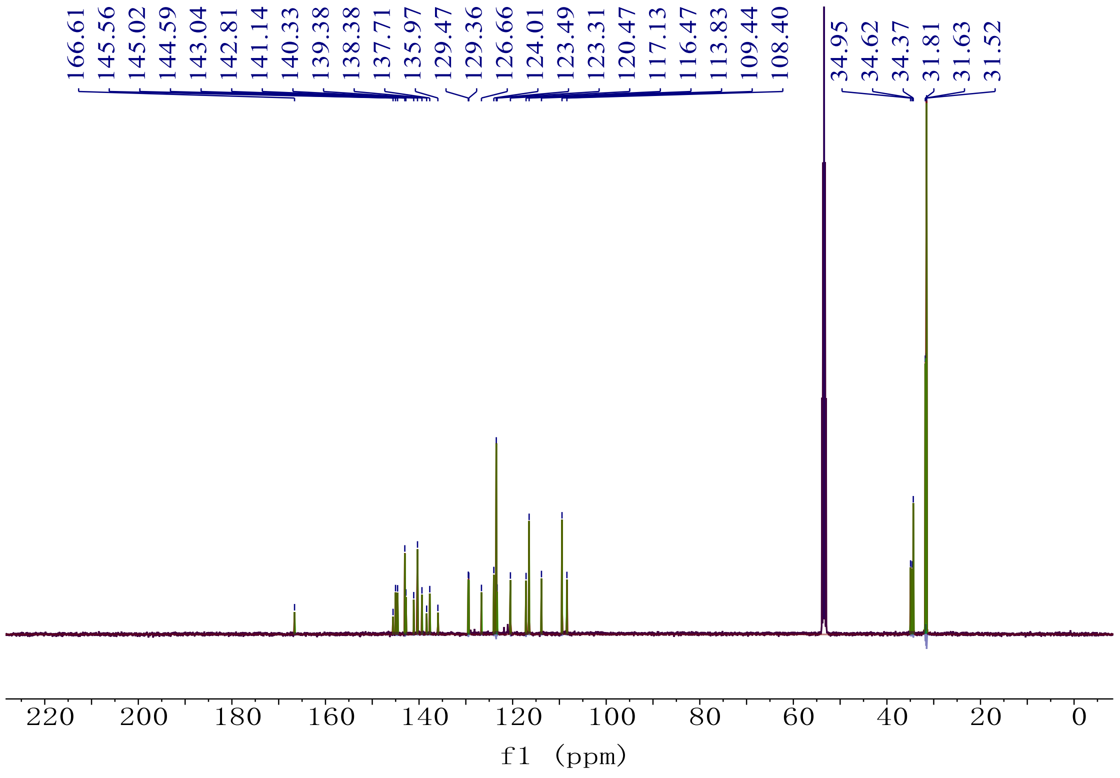

Figure **S15**. ^{13}C-NMR spectrum of **tBuCzCO$_2$HDCzB** in CD_2Cl_2.

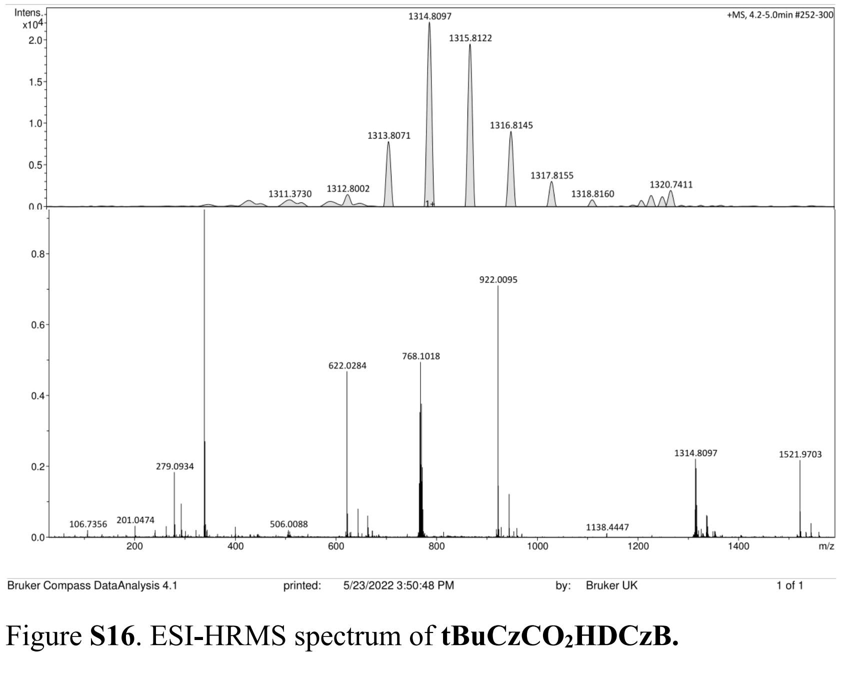

Figure **S16**. ESI-HRMS spectrum of **tBuCzCO$_2$HDCzB.**

<Sample Information>

Sample Name	: ws-dcz		
Sample ID	:		
Method Filename	: 80% THF 20% water 0.6 mlmin 20 mins.lcm		
Batch Filename	: shut down.lcb		
Vial #	: 2-11	Sample Type	: Unknown
Injection Volume	: 1 uL		
Date Acquired	: 14/07/2023 19:34:37	Acquired by	: System Administrator
Date Processed	: 14/07/2023 19:54:39	Processed by	: System Administrator

<Chromatogram>

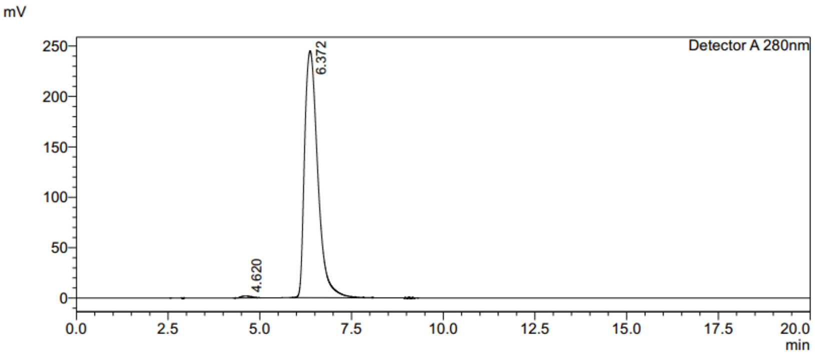

<Peak Table>

Detector A 280nm

Peak#	Ret. Time	Area	Height	Area%	Area/Height	Width at 5% Height
1	4.620	39558	1971	0.642	20.067	0.646
2	6.372	6118026	244701	99.358	25.002	0.842
Total		6157584	246672	100.000		

Figure **S17**. HPLC spectrum of **tBuCzCO$_2$HDCzB**.

Synthesis of 2GtBuCzCO$_2$HDCzB.

A similar procedure to **2GtBuCzCO$_2$HDCzB** was employed but using 3,5-difluorobenzonitrile and 3,6-di-tert-butyl-9H-carbazole. **Yield:** 38% (140 mg) **R$_f$:** 0.40 (DCM:hexane = 2:5) **Mp** > 400 °C. **^{1}H NMR (500 MHz, CD$_2$Cl$_2$) δ (ppm):** 9.08 (d, J = 2.0 Hz, 2H), 8.88 (s, 2H), 8.59 (d, J = 1.8 Hz, 2H), 8.01 (dd, J = 4.3, 2.0 Hz, 10H), 7.90 (d, J = 8.6 Hz, 4H), 7.79 (s, 2H), 7.74 – 7.69 (m, 6H), 7.56 (dd, J = 8.6, 2.0 Hz, 4H), 7.32 (dd, J = 8.8, 2.1 Hz, 2H), 6.85 (d, J = 176.9 Hz, 16H), 1.68 (s, 18H), 1.40 (s, 72H), 0.87 (s, 18H). **^{13}C NMR (126 MHz, CD$_2$Cl$_2$) δ (ppm):** 166.42, 145.27, 145.04, 143.03, 142.25, 141.24, 140.96, 139.62, 138.45, 137.91, 137.56, 136.80, 130.85, 130.38, 129.64, 126.51, 125.89, 124.80, 123.71, 123.37, 122.73, 121.50, 120.59, 119.20, 117.47, 115.76, 113.58, 111.33, 108.84, 107.15, 35.08, 34.48, 34.03, 31.90, 31.75, 30.70. **MALDI-TOF-MS (m/z)**: [M]$^+$ Calculated for **$C_{157}H_{159}BN_8O_2$:** 2199.2679; **Found:** 2199.2870. **Elemental analysis**: Calcd. for **$C_{157}H_{159}BN_8O_2$**: C, 85.68%, H, 7.28%, and N, 5.09%. Anal. C, 85.24%, H, 7.20%, and N, 5.21%. **HPLC analysis**, 99.38% pure, retention time 8.699 min in 82% THF, 18% water.

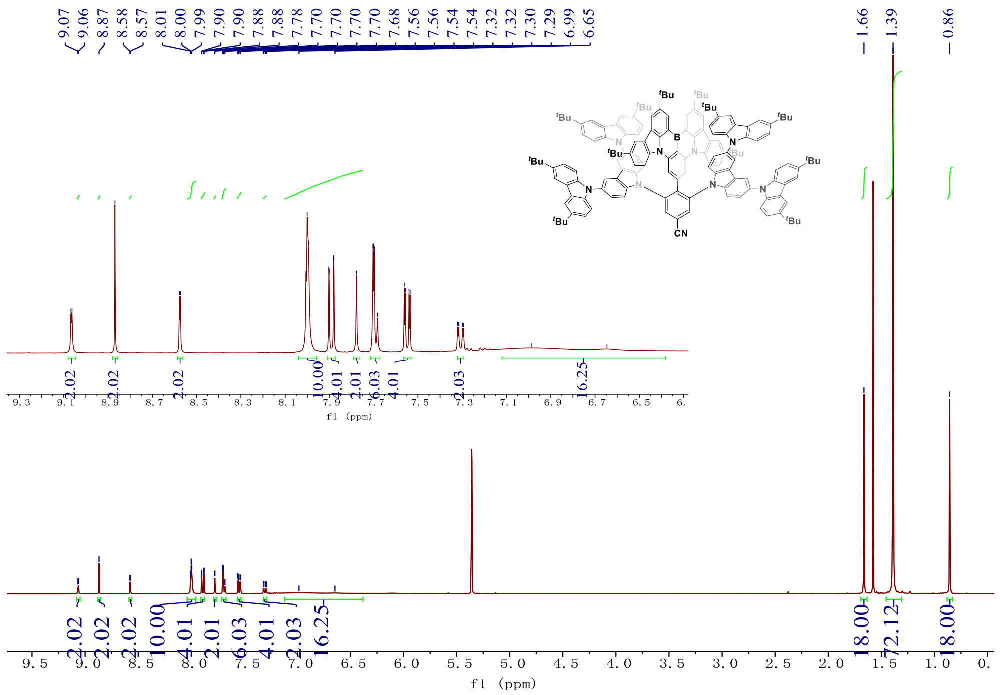

Figure **S18**. ^{1}H-NMR spectrum of **2GtBuCzCO$_2$HDCzB** in CD_2Cl_2.

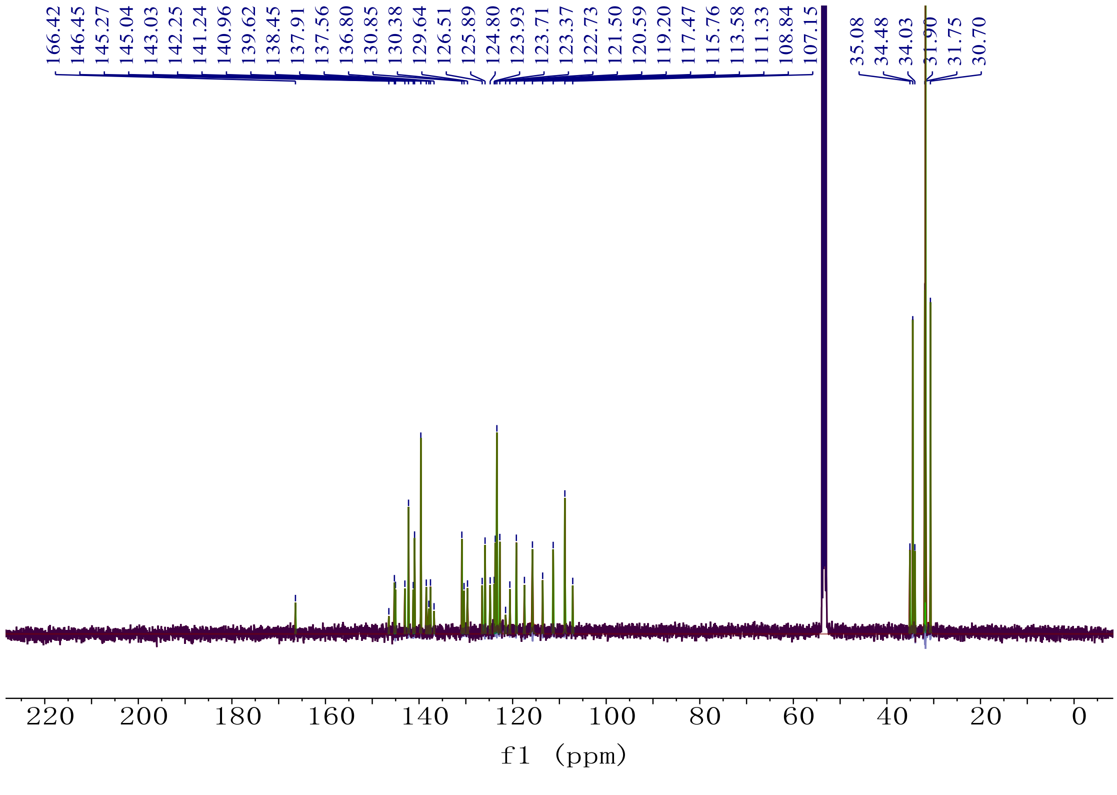

Figure **S19**. ^{13}C-NMR spectrum of **2GtBuCzCO$_2$HDCzB** in CD_2Cl_2.

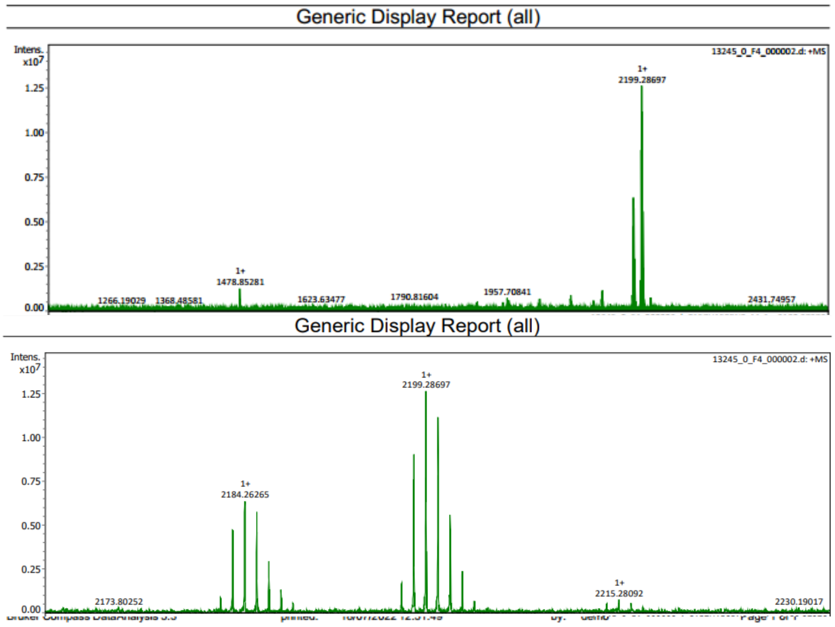

Figure **S20**. MALDI-MS spectrum of **2GtBuCzCO$_2$HDCzB**.

<Sample Information>

Sample Name	: ws-ddcz		
Sample ID	:		
Method Filename	: 82% THF 18% water 0.6 mlmin 20 mins.lcm		
Batch Filename	: shut down.lcb		
Vial #	: 2-10	Sample Type	: Unknown
Injection Volume	: 1 uL		
Date Acquired	: 14/07/2023 16:03:56	Acquired by	: System Administrator
Date Processed	: 14/07/2023 16:23:59	Processed by	: System Administrator

<Chromatogram>

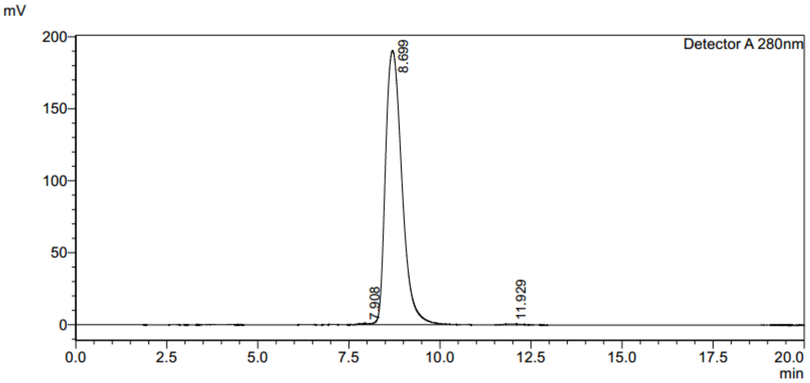

<Peak Table>

Detector A 280nm

Peak#	Ret. Time	Area	Height	Area%	Area/Height	Width at 5% Height
1	7.908	17573	819	0.291	21.470	--
2	8.699	5996413	190425	99.378	31.490	1.044
3	11.929	19981	585	0.331	34.156	0.964
Total		6033967	191829	100.000		

Figure **S21**. HPLC of **2GtBuCzCO$_2$HDCzB**.

Computations

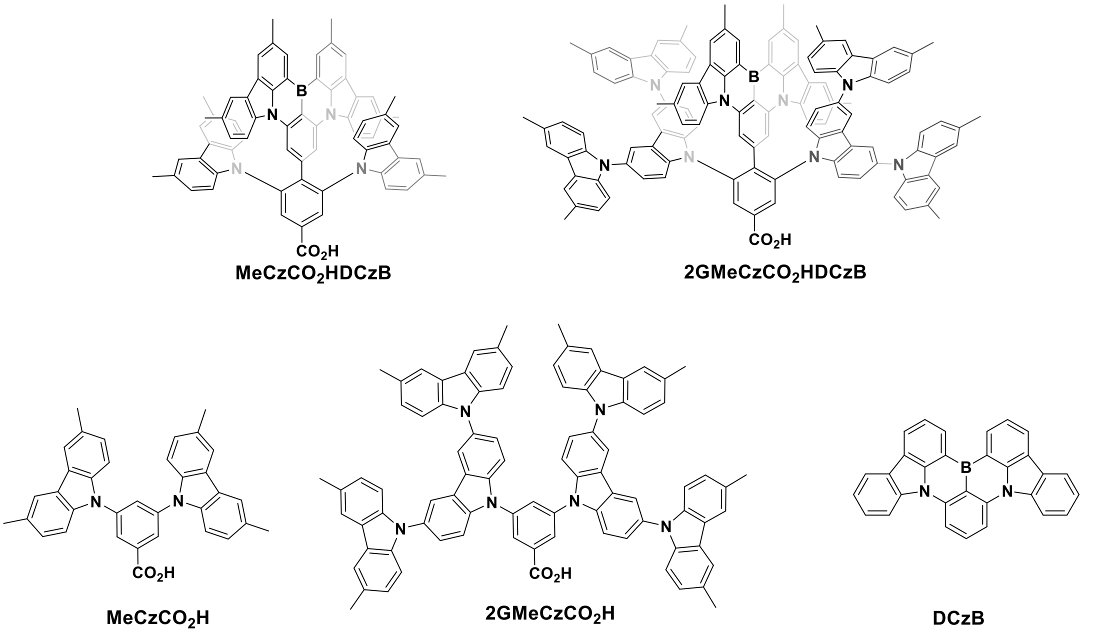

Figure **S22**. The structures of the model compounds in the DFT study where the *tert*-butyl groups have been replaced by methyl groups.

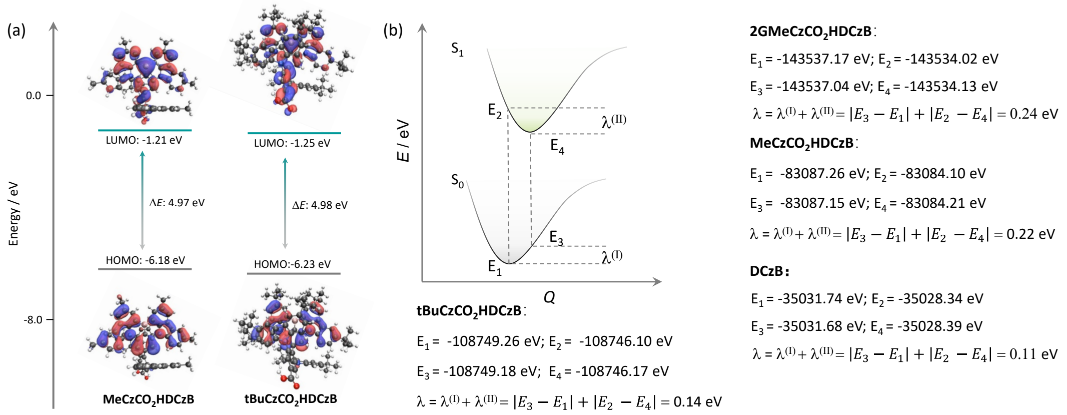

Figure **S23**. (a) Calculated HOMOs and LUMOs distribution for **tBuCzCO₂HDCzB** and **MeCzCO₂HDCzB** (isovalue: 0.02) performed by TD-DFT calculation at M06-2X/6-31G(d,p) level in the gas phase. (b) The potential energy scheme that describes the methodology to determine λ. S_0: the ground-state; S_1: excited singlet. E_1: the energy at ground-state geometry; E_2: the energy at first excited singlet state at ground-state geometry; E_3: the energy at optimized first excited singlet state geometry; E_4: the energy at first excited singlet state at optimized first excited singlet state geometry.

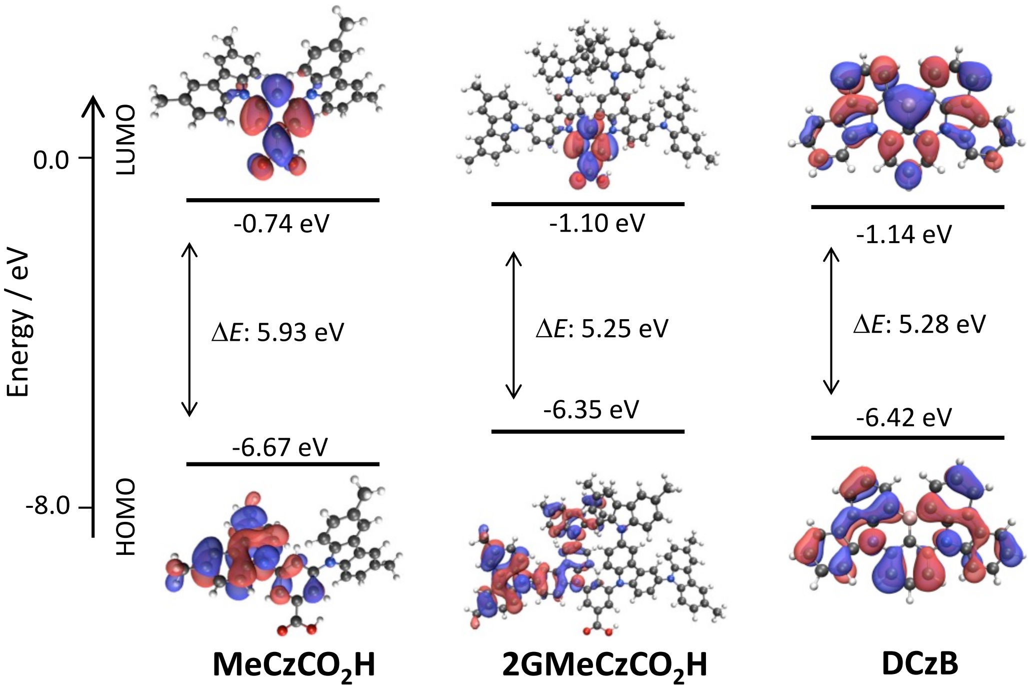

Figure **S24**. Calculated HOMOs and LUMOs distribution for **MeCzCO$_2$H**, **2GMeCzCO$_2$H** and **DCzB** (isovalue: 0.02) performed by TD-DFT calculation at M06-2X/6-31G(d,p) level in the gas phase.

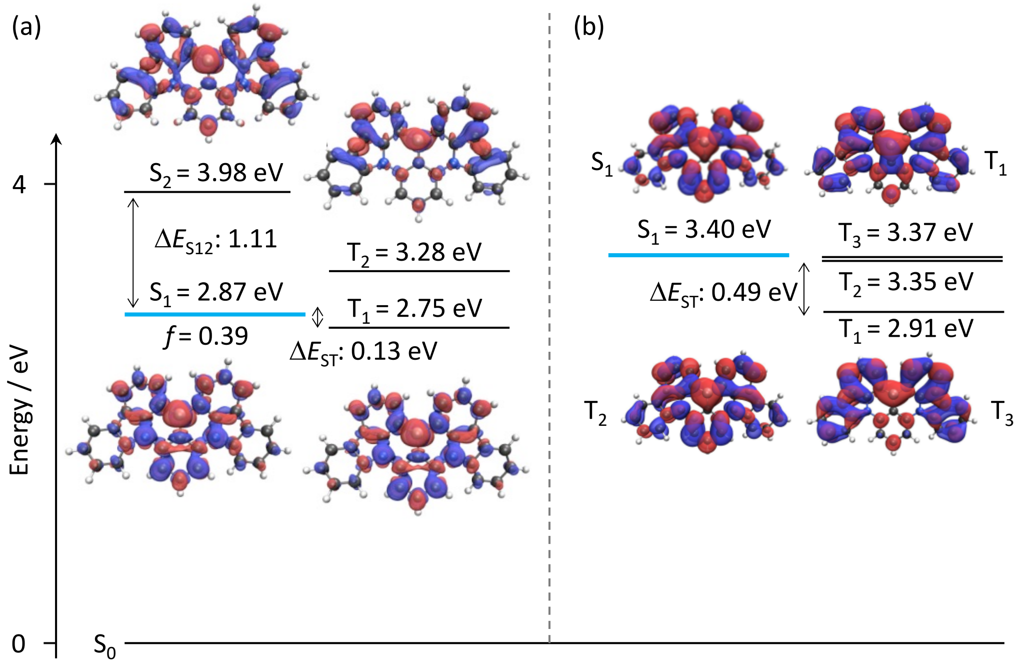

Figure **S25**. (a) Difference density plots (isovalue: 0.001) of S_1/S_2 and T_1/T_2 excited states (calculated in the gas phase at the SCS-ADC(2)/cc-pVDZ level) for **DCzB**. *f* is the oscillator strength. (b) The natural transition orbitals (NTOs) (isovalue: 0.02) for S_1, T_1, T_2 and T_3 for **DCzB** performed by TD-DFT calculation at M06-2X/6-31G(d,p) in the gas phase.

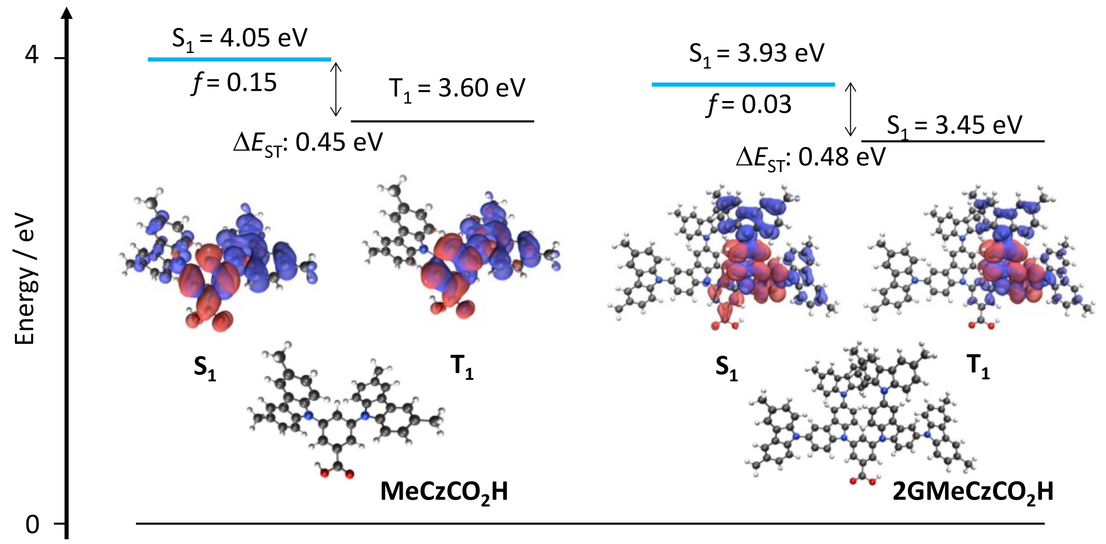

Figure **S26**. Geometrical configuration and the natural transition orbitals (NTOs) for $S_0 \rightarrow S_1$ and $S_0 \rightarrow T_1$ transition (isovalue: 0.02) performed by TD-DFT calculation at M06-2X/6-31G(d,p) in the gas phase.

Table **S1**. Calculated data in the gas phase at the M06-2X/6-31G(d,p) level.

**Compound**	**FMOs / eV**	**S_1 /eV**	**T_1 / eV**	**ΔE_{ST} / eV**	***f***
**DCzB**	H: -6.42; L: -1.14	3.40	2.91	0.49	0.50
**MeCzCO$_2$HDCzB**	H: -6.18; L: -1.21	3.16	2.71	0.46	0.33
**2GtMeCzCO$_2$HDCzB**	H: -6.20; L: -1.49	3.15	2.69	0.46	0.32
**MeCzCO$_2$H**	H: -6.67; L: -0.74	4.05	3.60	0.45	0.45
**2GtMeCzCO$_2$H**	H: -6.35; L: -1.10	3.93	3.45	0.48	0.03

Where H=HOMO， L=LUMO.

Optoelectronic Characterization

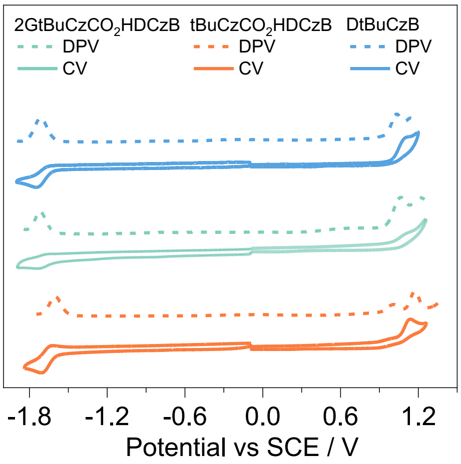

Figure **S27**. Cyclic voltammogram (CV) and differential pulse voltammetry (DPV) in degassed DMF with 0.1 M [^{n}Bu$_4$N]PF$_6$ as the supporting electrolyte and Fc/Fc$^+$ as the internal reference versus SCE (0.45 V vs. DCM).[13]

Table **S2**. Electrochemical data

Material	E_{ox} /V[a]	E_{red} /V[a]	ΔE /V[b]	HOMO / eV[c]	LUMO /eV[c]
**DtBuCzB**	1.03	-1.73	2.76	-5.37	-2.61
**2GtBuCzCO$_2$HDCzB**	1.06	-1.71	2.77	-5.40	-2.63
**tBuCzCO$_2$HDCzB**	1.01	-1.61	2.62	-5.35	-2.73

[a] E_{ox} and E_{red} are the peak of anodic and cathodic potentials from DPV versus SCE. In degassed DMF with 0.1 M [^{n}Bu$_4$N]PF$_6$ as the supporting electrolyte and Fc/Fc$^+$ as the internal reference (0.45 V vs. SCE).[13] [b] $\Delta E_g = E_{ox}$-E_{red}. [c] $E_{HOMO/LUMO}$ = -(E_{ox} / E_{red} vs. Fc/Fc$^+$ + 4.8) eV.[14]

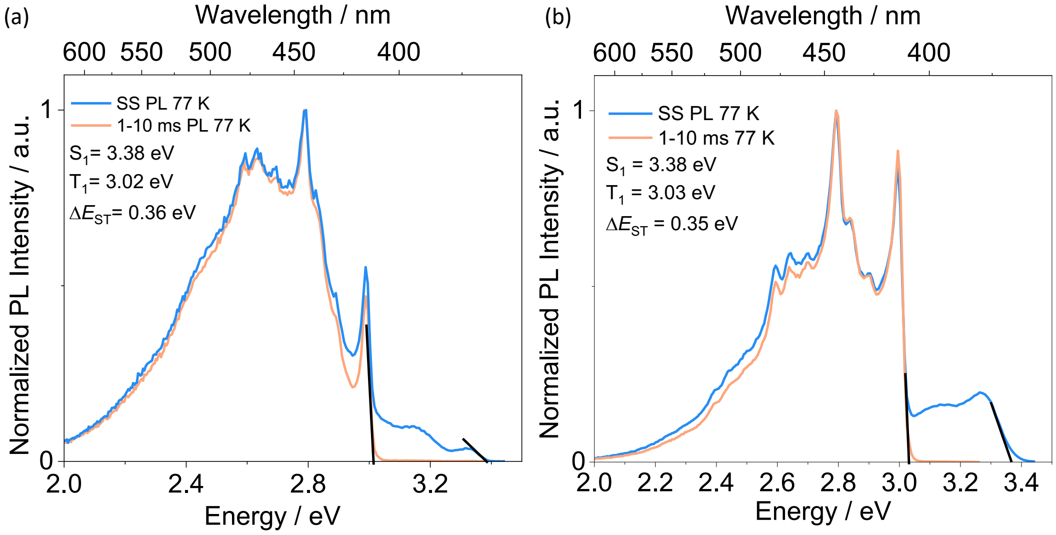

Figure **S28**. Steady-state PL and delayed emission spectra (1–10 ms) at 77 K of (a) **tBuCzCO$_2$H** and (b) **tBuCzCN** in toluene, λ_{exc} = 340 nm.

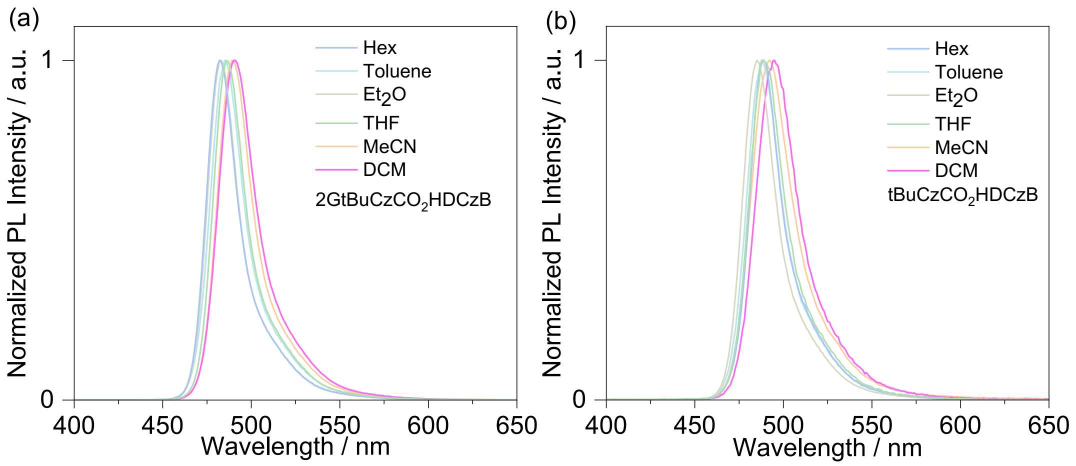

Figure **S29**. Solvatochromism PL study for **2GtBuCzCO₂HDCzB** and **tBuCzCO₂HDCzB**, λ_{exc} = 340 nm.

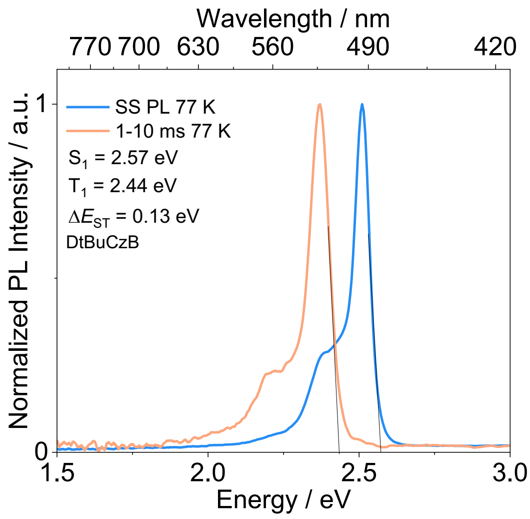

Figure **S30**. Steady-state PL and delayed emission spectra (1–10 ms) at 77 K of **DtBuCzB** in toluene, λ_{exc} = 340 nm.

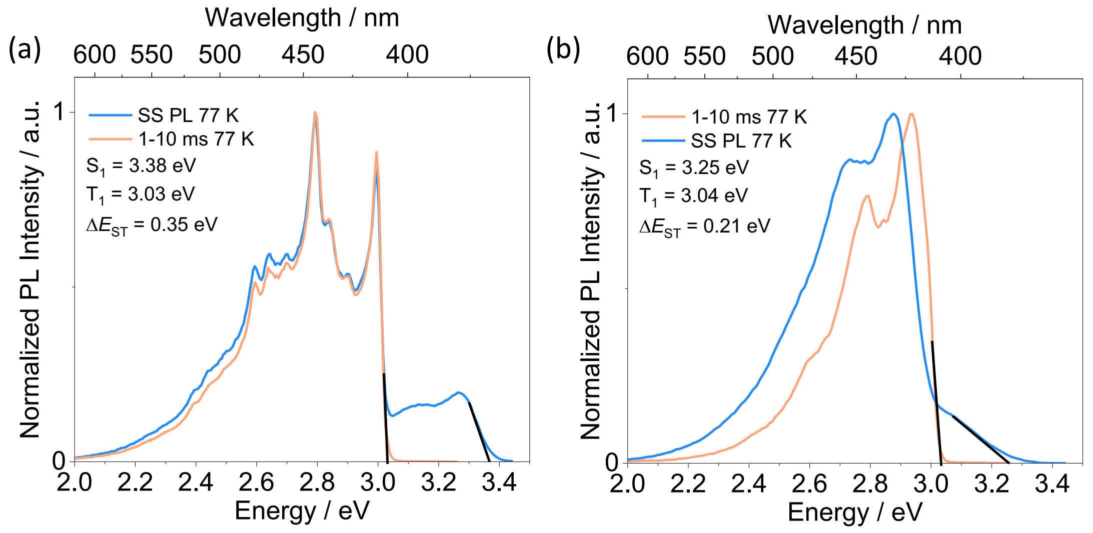

Figure **S31**. Steady-state PL and delayed emission spectra (1–10 ms) at 77 K of (a) **tBuCzCN** and (b) **2GtBuCzCN** in toluene, λ_{exc} = 340 nm.

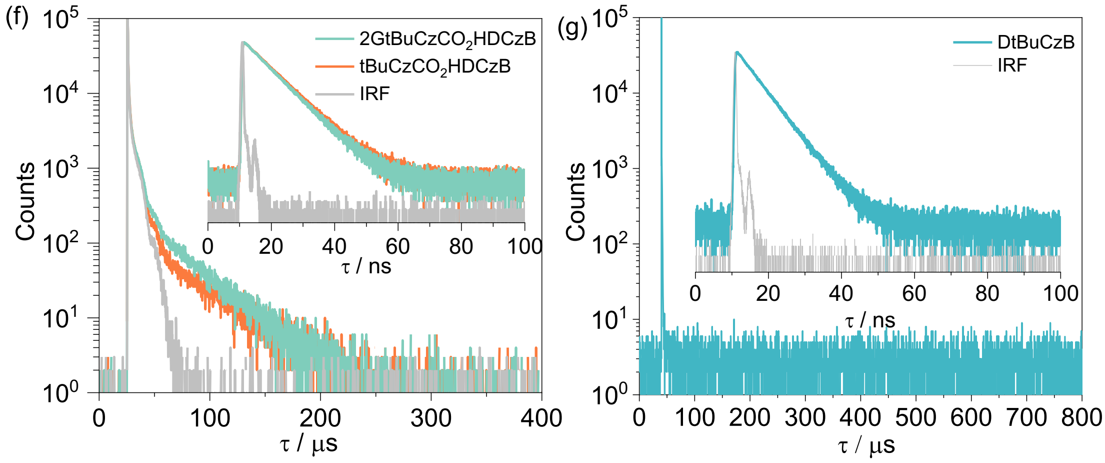

Figure **S32**. Time-resolved PL decay of (a) **2GtBuCzCO₂HDCzB** and **tBuCzCO₂HDCzB**, and (b) **DtBuCzB** in degassed toluene, λ_{exc} = 340 nm. Inset: Time-resolved PL decay of the prompt component, λ_{exc} = 375 nm.

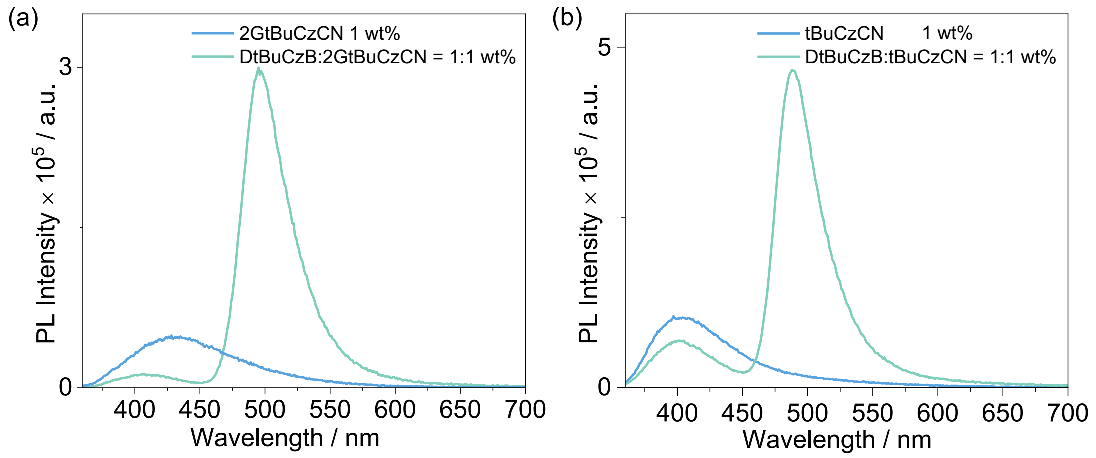

Figure **S33**. Steady-state PL of (a) **2GtBuCzCN** and (b) **tBuCzCN** and their mixtures with **DtBuCzB** in 1:1 ratio of 1 wt% doped film in PMMA, λ_{exc} = 340 nm.

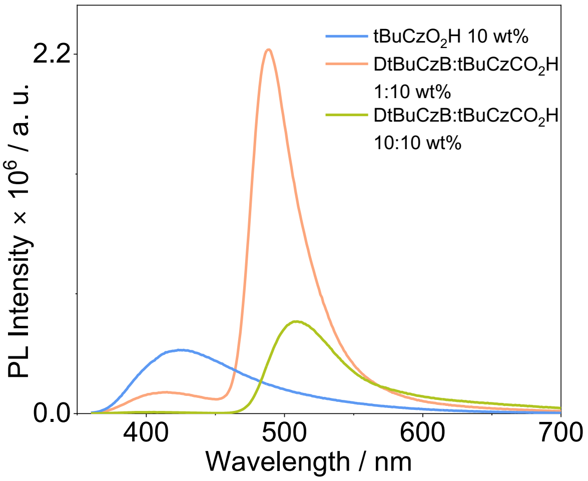

Figure **S34**. Steady-state PL spectra of **2GtBuCzCO₂H** and its mixtures with **DtBuCzB** in 10:10 ratio of 10 wt% doped film in PMMA, λ_{exc} = 340 nm.

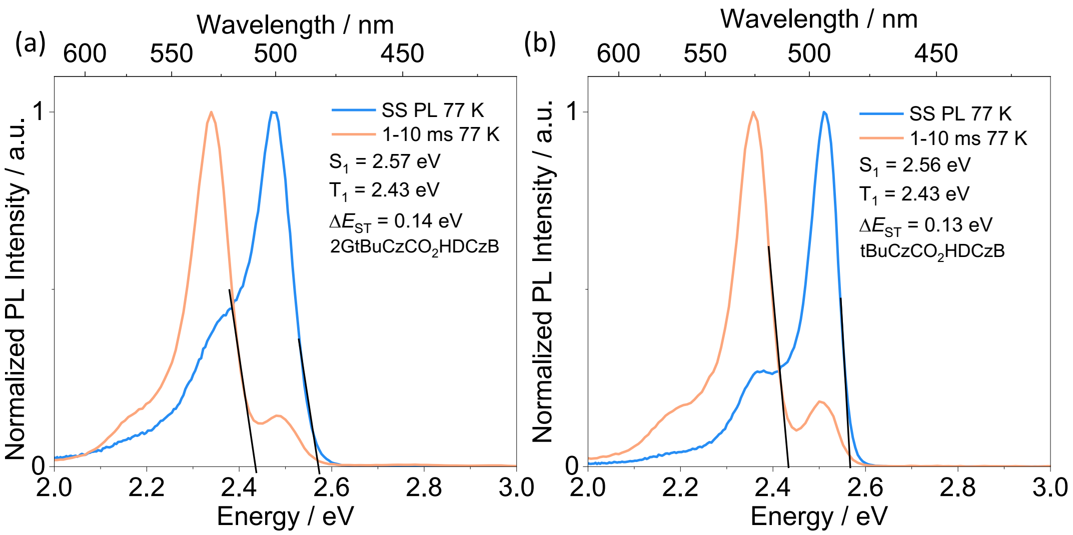

Figure **S35.** Steady-state PL and delayed emission spectra (1–10 ms) at 77 K of (a) **2GtBuCzCO₂HDCzB** and (b) **tBuCzCO₂HDCzB** in 1 wt% doped films in PMMA, $\lambda_{exc} = 340$ nm.

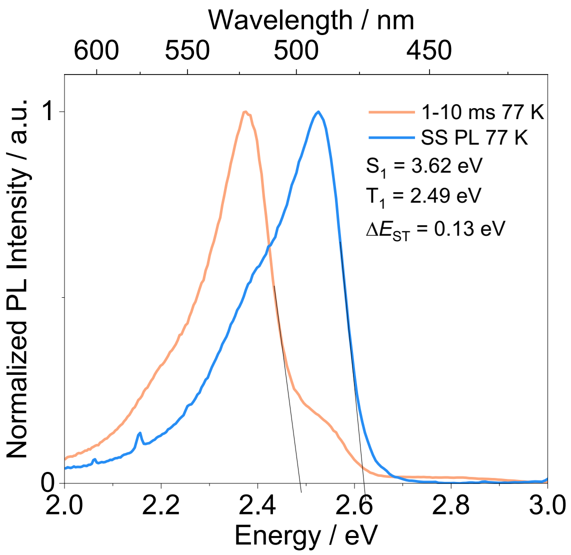

Figure **S36.** Steady-state PL and delayed emission spectra (1–10 ms) at 77 K of **DtBuCzB** in 1 wt% doped film in PMMA, $\lambda_{exc} = 340$ nm.

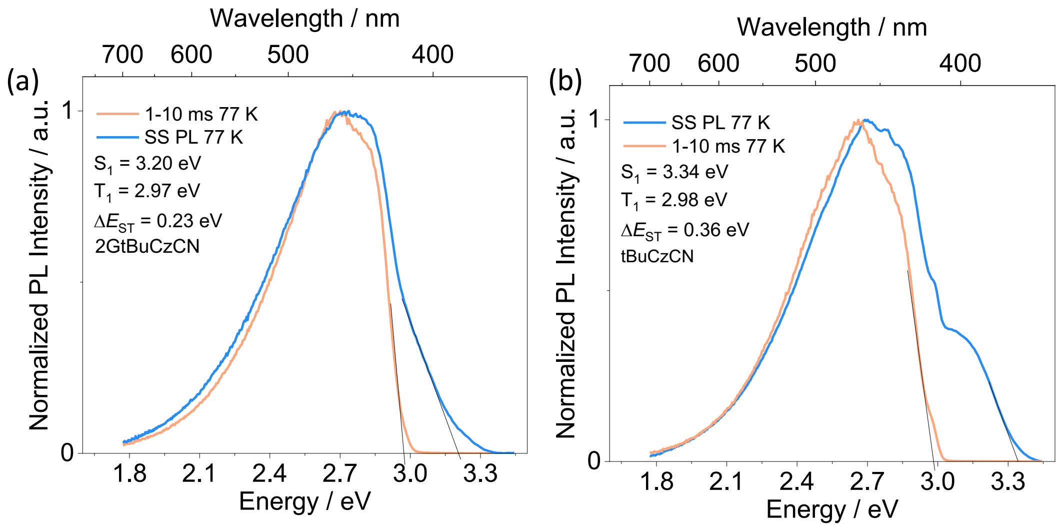

Figure **S37.** Steady-state PL and delayed emission spectra (1–10 ms) at 77 K of (a) **2GtBuCzCN** and (b) **tBuCzCN** in 1 wt% doped film in PMMA, λ_{exc} = 340 nm.

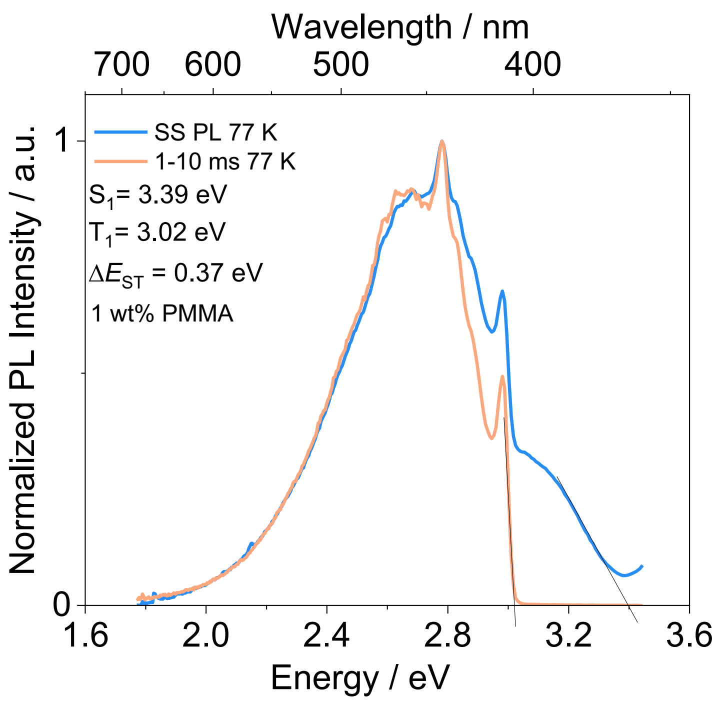

Figure **S38.** Steady-state PL and delayed emission spectra (1–10 ms) at 77 K of **tBuCzCO₂H** in 1 wt% doped film in PMMA, λ_{exc} = 340 nm.

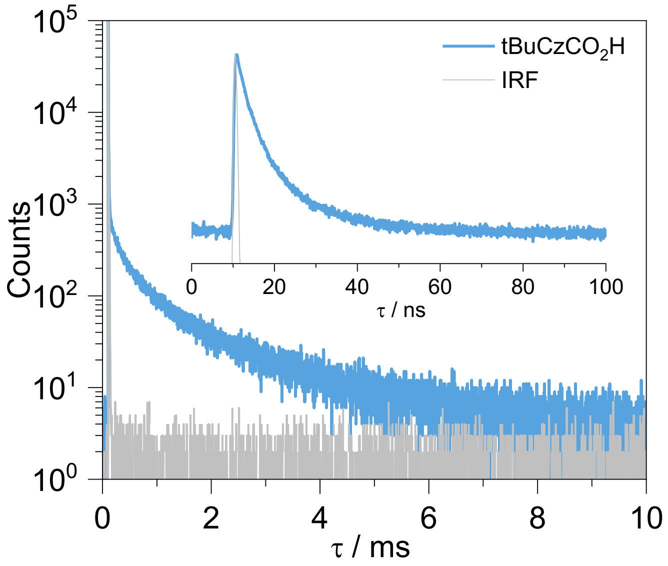

Figure **S39**. Time-resolved PL decay of **$tBuCzCO_2H$** in 1 wt% doped film in PMMA, λ_{exc} = 340 nm, and inset figure is the TRPL of the prompt component, λ_{exc} = 375 nm.

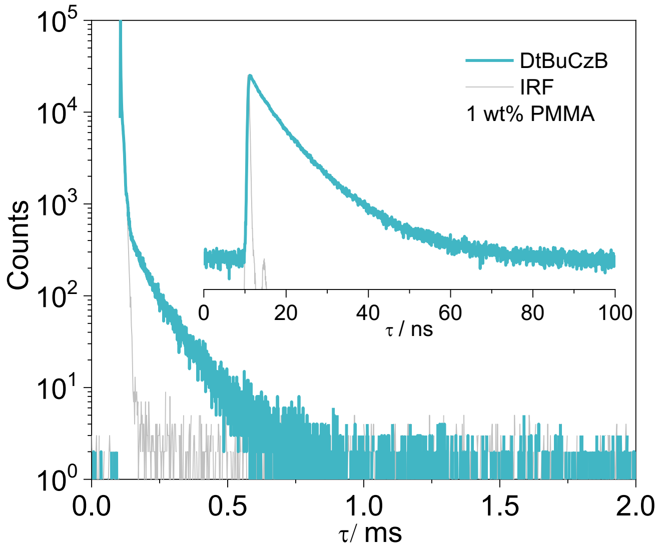

Figure **S40.** Time-resolved PL decay of **DtBuCzB** in 1 wt% doped film in PMMA, λ_{exc} = 340 nm (inset: the TRPL of the prompt component), λ_{exc} = 375 nm.

Table **S3**. Photophysical data in 1 wt% doped films in PMMA.

Emitter	λ_{PL} /nm[a]	FWHM / nm[b]	Φ_{PL} / %[c]	S_1 /eV[d]	T_1 /eV[e]	ΔE_{ST} /eV[f]	τ_p /ns[g]	τ_d /µs[g]
**DtBuCzB**	488	37	43	2.62	2.49	0.13	7.6	85
**tBuCzCO₂HDCzB**	495	29	56	2.56	2.43	0.13	8.1	91
**2GtBuCzCO₂HDCzB**	490	23	71	2.57	2.43	0.14	8.3	159

[a]Obtained at 298 K, λ_{exc} = 340 nm. [b]Full-width at half-maximum; [c]The Φ_{PL} was measured in an integrating sphere under nitrogen (λ_{exc} = 340 nm). [d]Obtained from the onset of the SS PL spectrum at 77 K. [e]Obtained from the onset of the delayed emission spectrum (1-10 ms) at 77 K (λ_{exc} = 340 nm). [f]$\Delta E_{ST} = E(S_1) - E(T_1)$. [g]Measured at 300 K under vacuum, λ_{exc} = 379 nm.

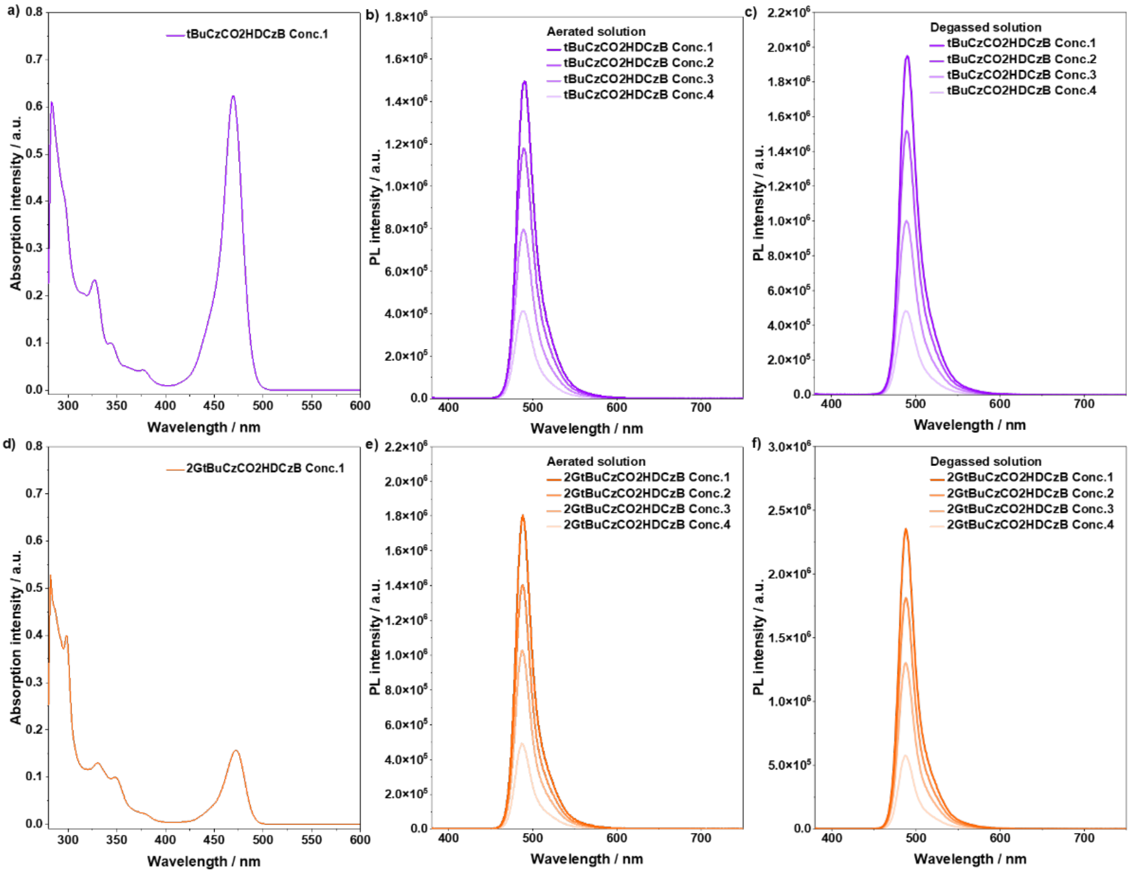

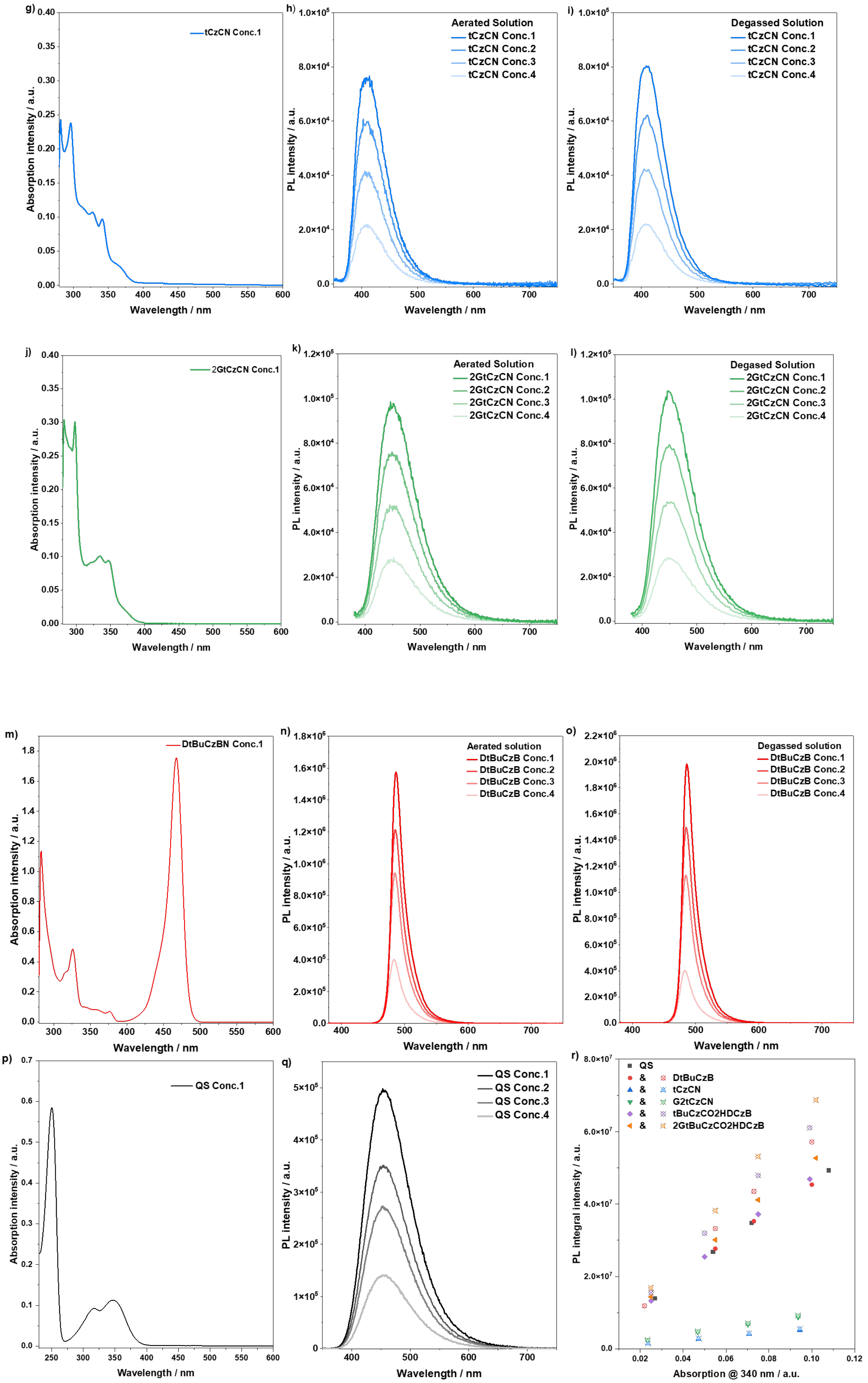
g)
tCzCN Conc.1
Absorption intensity / a.u.
Wavelength / nm
h)
Aerated Solution
tCzCN Conc.1
tCzCN Conc.2
tCzCN Conc.3
tCzCN Conc.4
PL intensity / a.u.
Wavelength / nm
i)
Degassed Solution
tCzCN Conc.1
tCzCN Conc.2
tCzCN Conc.3
tCzCN Conc.4
PL intensity / a.u.
Wavelength / nm
j)
2GtCzCN Conc.1
Absorption intensity / a.u.
Wavelength / nm
k)
Aerated Solution
2GtCzCN Conc.1
2GtCzCN Conc.2
2GtCzCN Conc.3
2GtCzCN Conc.4
PL intensity / a.u.
Wavelength / nm
l)
Degased Solution
2GtCzCN Conc.1
2GtCzCN Conc.2
2GtCzCN Conc.3
2GtCzCN Conc.4
PL intensity / a.u.
Wavelength / nm
m)
DtBuCzBN Conc.1
Absorption intensity / a.u.
Wavelength / nm
n)
Aerated solution
DtBuCzB Conc.1
DtBuCzB Conc.2
DtBuCzB Conc.3
DtBuCzB Conc.4
PL intensity / a.u.
Wavelength / nm
o)
Degassed solution
DtBuCzB Conc.1
DtBuCzB Conc.2
DtBuCzB Conc.3
DtBuCzB Conc.4
PL intensity / a.u.
Wavelength / nm
p)
QS Conc.1
Absorption intensity / a.u.
Wavelength / nm
q)
QS Conc.1
QS Conc.2
QS Conc.3
QS Conc.4
PL intensity / a.u.
Wavelength / nm
r)
QS
& DtBuCzB
& tCzCN
& G2tCzCN
& tBuCzCO2HDCzB
& 2GtBuCzCO2HDCzB
PL integral intensity / a.u.
Absorption @ 340 nm / a.u.

Figure **S41**. Absorption and PL intensity of **tBuCzCO₂HDCzB** (a)-(c), **2GtBuCzCO₂HDCzB** (d)-(f), **tCzCN** (g)-(i), **G2tCzCN** (j)-(l), **DtBuCzB** (m)-(o), quinine sulphate (QS) (p)-(q), and (r) their absorption intensity *versus* PL integral intensity in progressively diluted toluene solution, (Solid symbols: aerated solution, hollow symbols: degassed solution) (λ_{exc}: 340 nm).

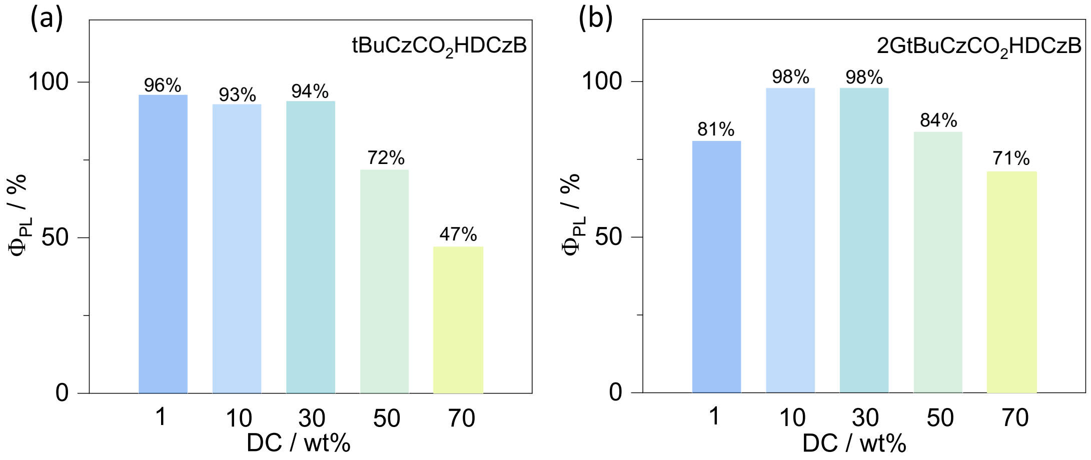

Figure **S42**. Concentration-dependent Φ_{PL} of (a) **tBuCzCO$_2$HDCzB** and (b) **2GtBuCzCO$_2$H-DCzB** in mCP doped films under nitrogen, λ_{exc} = 340 nm, DC is the doping concentration.

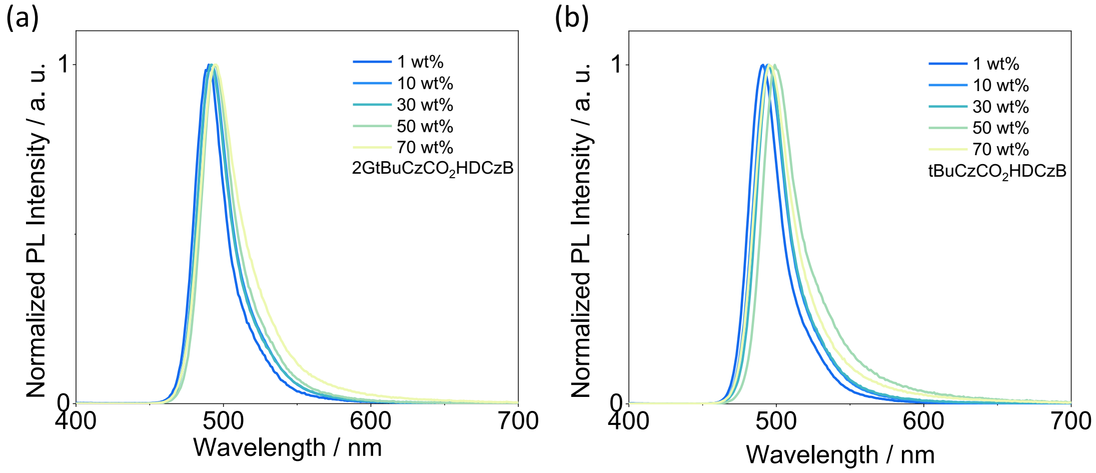

Figure **S43**. Concentration-dependent PL of (a) **2GtBuCzCO$_2$HDCzB** and (b) **tBuCzCO$_2$H-DCzB** in mCP doped films, λ_{exc} = 340 nm.

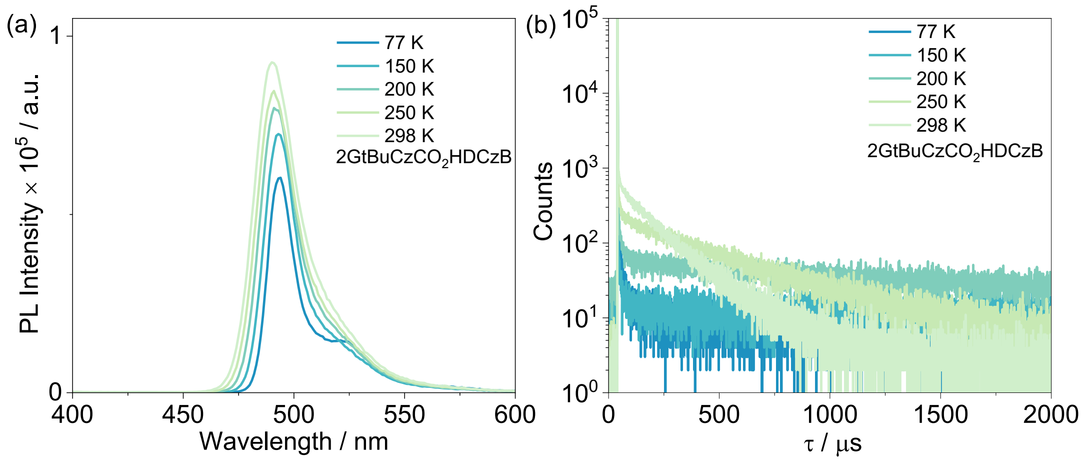

Figure **S44**. Temperature-dependent (a) SS-PL spectra and (b) TR-PL decays of **2GtBuCzCO$_2$HDCzB** in 1 wt% doped films in PMMA, λ_{exc} = 379 nm.

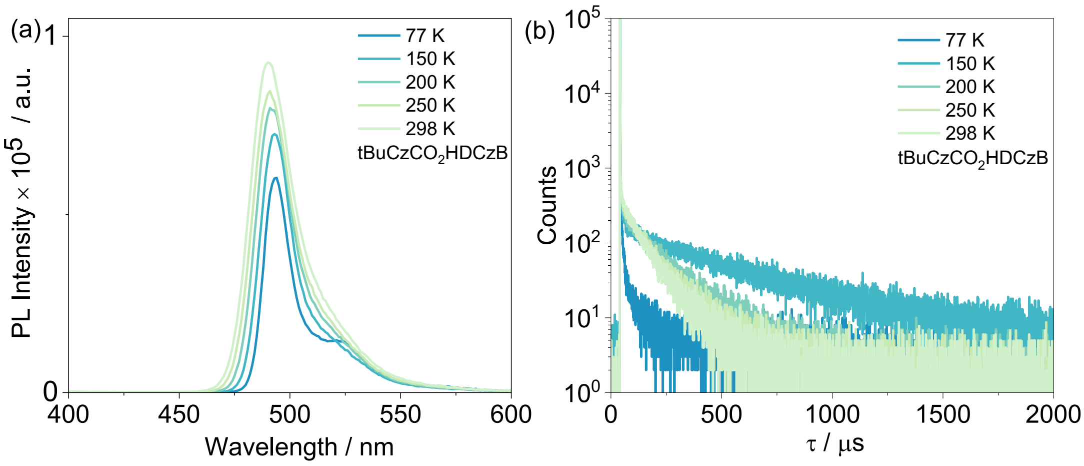

Figure **S45**. Temperature-dependent (a) SS-PL spectra and (b) TR-PL decays of **tBuCzCO$_2$HDCzB** in 1 % doped films in PMMA, λ_{exc} = 379 nm.

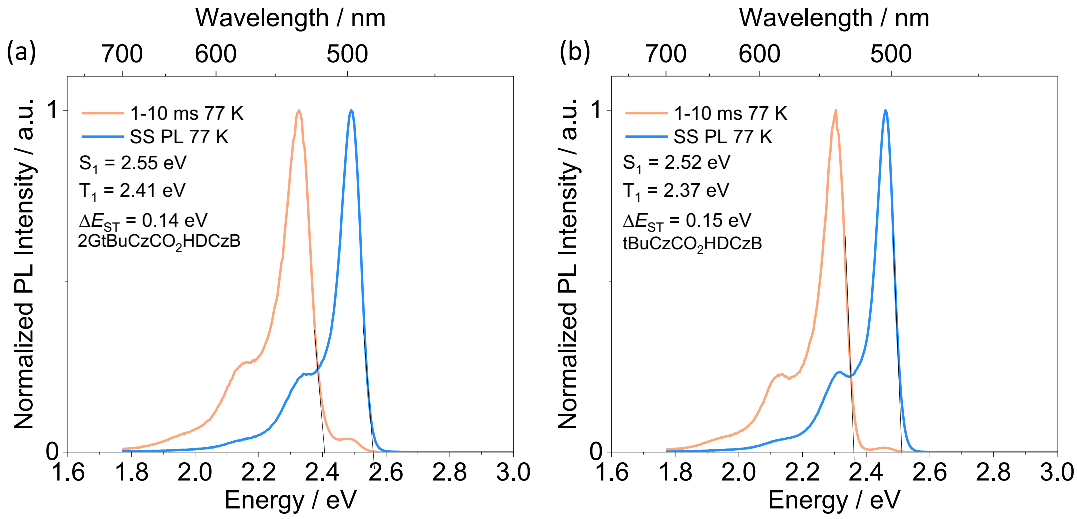

Figure **S46**. Steady-state PL and delayed emission spectra (1–10 ms) at 77 K of (a) **2GtBuCzCNDCzB** and (b) **tBuCzCNDCzB** in 30 wt% doped films in mCP, λ_{exc} = 340 nm.

For a TADF system, the main exciton loss channels are either singlet or triplet nonradiative transition processes. Considering the high Φ_{PL} > 90% of **tBuCzCNDCzB** and (b) **2GtBuCzCNDCzB**, the singlet nonradiative decay (k_{nr}^{S}) can be assumed to be 0 s^{-1}, therefore the exciton loss can be attributed to the triplet nonradiative transition process (k_{nr}^{T}). The kinetics parameters were calculated according to our previous reported method and summarized in Table **S4**.[17,18]

Table **S4** Summary of kinetics parameters.

**Compounds**	**Φ_{PL} /%**	**Φ_{P} /%**	**Φ_{d} /%**	**τ_p/ns, τ_d/ μs**	**k_p / 10^8 s^{-1}**	**k_d / 10^3 s^{-1}**	**k_r^S /10^7 s^{-1}**	**k_{nr}^T /10^3 s^{-1}**	**k_{ISC} /10^7 s^{-1}**	**k_{RISC} /10^4 s^{-1}**
**DtBuCzB**[a]	43	37	6	7.6, 85	1.32	11.8	4.87	10.6	8.29	0.30
**tBuCzCO2HDCzB**[a]	56	39	17	8.1, 91	1.23	11.0	4.81	7.93	7.53	0.79
**2GtBuCzCO2HDCzB**[a]	71	37	34	8.3, 159	1.28	6.29	4.46	2.90	7.59	0.92
**tBuCzCO2HDCzB**[b]	94	44	50	6.4, 143	1.56	6.99	7.81	0.84	7.81	1.23
**2GtBuCzCO2HDCzB**[b]	98	40	58	7.0, 102	1.43	9.82	5.71	0.32	8.57	2.37

[a] Measured in spin-coated 1 wt% doped thin films in PMMA; [b] Measured in spin-coated 30 wt% doped thin films in mCP.

OLEDs

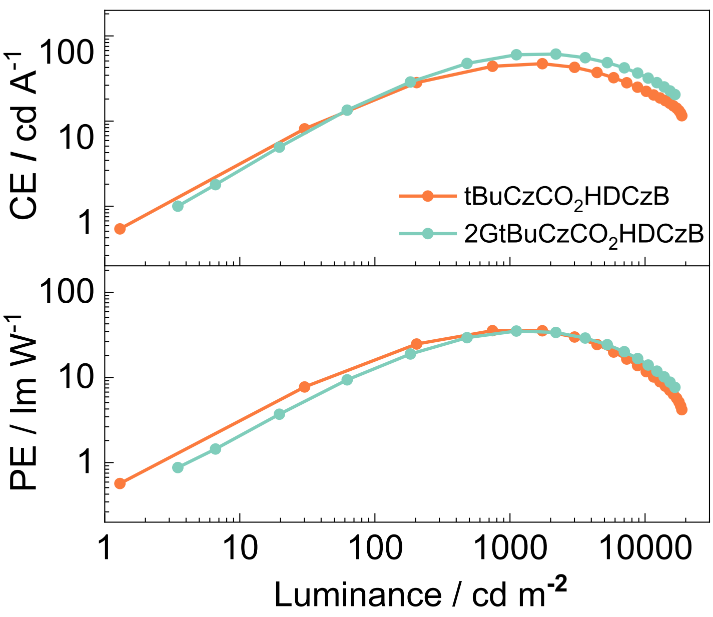

Figure **S47**. Current efficiency and power efficiency versus luminance curves for solution-processed devices with **tBuCzCO₂HDCzB** and **2GtBuCzCO₂HDCzB** in mCP.

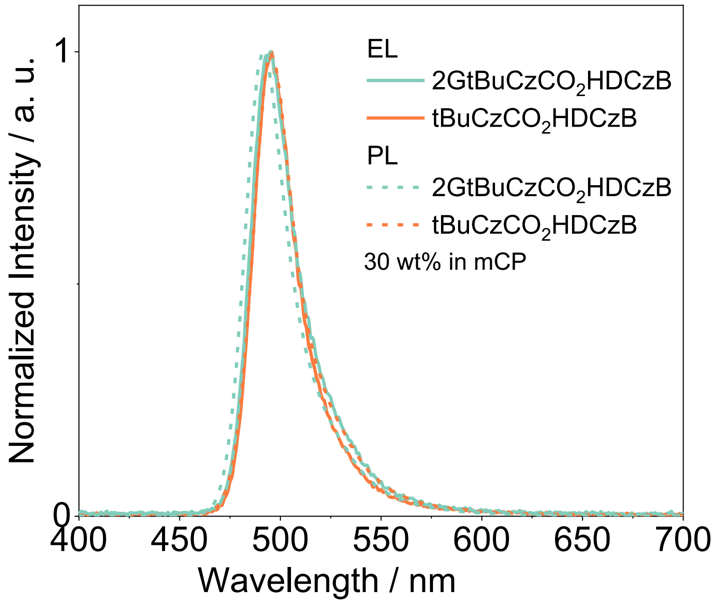

Figure **S48**. Comparation of SS-PL (λ_{exc} = 340 nm) and EL spectra of **tBuCzCO₂HDCzB** and **2GtBuCzCO₂HDCzB**.

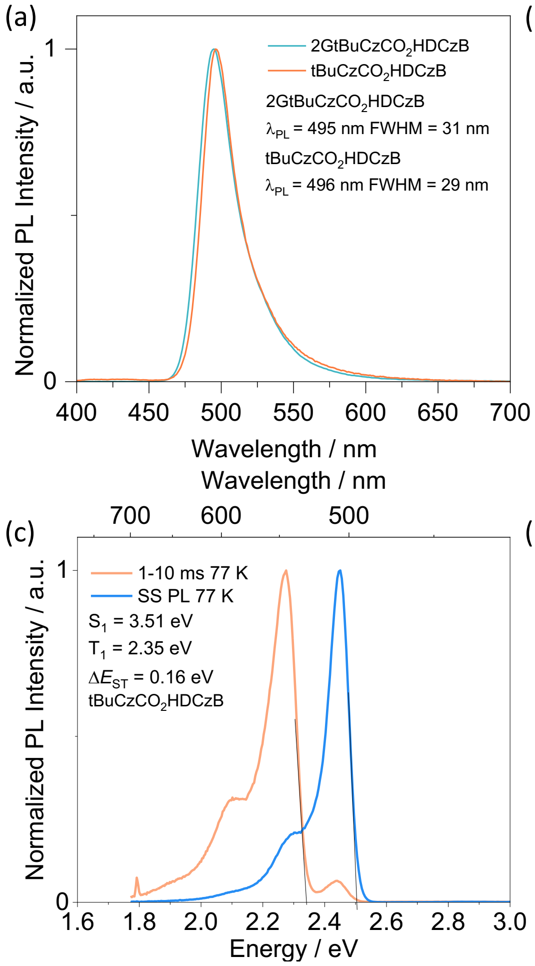

Figure **S49**. (a) Steady-state PL spectra of **tBuCzCO$_2$HDCzB** and **2GtBuCzCO$_2$HDCzB** in films with 30 wt% OXD-7, λ_{exc} = 340 nm. (B) Time-resolved PL decays, λ_{exc} = 340 nm, in in neat films with 30 wt% OXD-7. Steady-state PL and delayed emission spectra (1–10 ms) at 77 K of (c) **tBuCzCO$_2$HDCzB** and (d) **2GtBuCzCO$_2$HDCzB** in films with 30 wt% OXD-7, λ_{exc} = 340 nm.

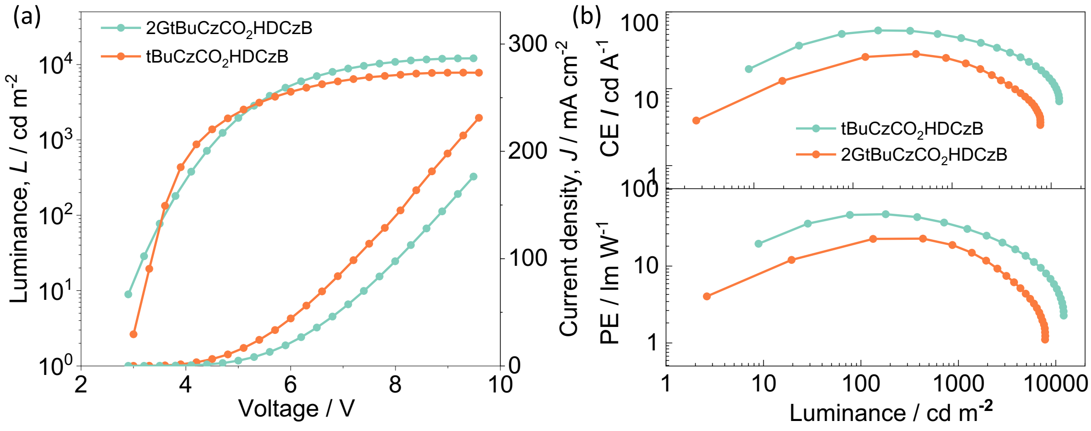

Figure **S50**. (a) Current density and luminance versus voltage characteristics. (b) Current efficiency and power efficiency versus luminance curves for solution-processed devices.

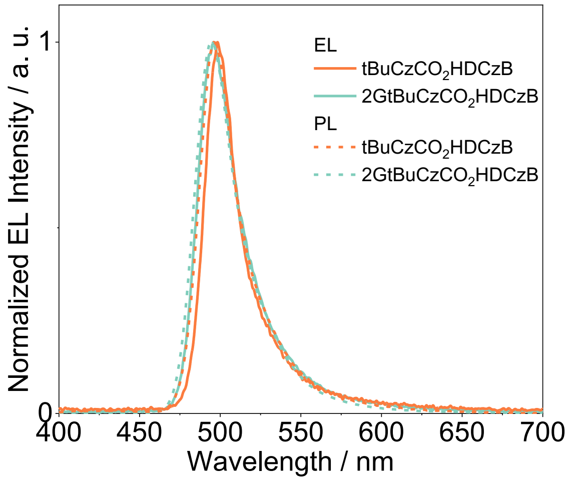

Figure **S51**. Comparation of SS-PL (λ_{exc} = 340 nm) and EL spectrum of **tBuCzCO₂HDCzB** and **2GtBuCzCO₂HDCzB.**

Table **S5** Key parameters of reported green solution-processed TADF OLEDs (EQE_{max}>10%).

TADF type	Compound	EML	λ_{EL} / nm	CIE (x,y)	EQE_{max} / %	EQE_{1000} / %	Ref
D-A TADF	**YD–TF**	Neat	552	0.41, 0.54	21.9	18.6	[19]
	**DCz-DPS-Cz**	Neat	498	0.23, 0.42	23.3	18.7	[20]
	**DCz-DPS-TBuCz**	Neat	500	0.24, 0.45	24.0	21.4	
	**tBuCz4mTRZ**	Neat	536	0.36, 0.58	23.8	N/A	[21]
	**tBuCz2m2pTRZ**	Neat	540	0.37, 0.37	28.7	N/A	[22]
	**tBuTBuCz-DPyM**	8 wt% in mCBP	503	0.25, 0.48	20.4	N/A	[23]
	**tBuG2B**	Neat	502	0.27, 0.52	17.0	14.7	[24]
	**CDE1**	Neat	552	0.40, 0.54	13.8	12.0	[25]
	**CzDMAC-DPS**	Neat	504	0.22, 0.44	12.2	11.7	[26]
	**BD–Cy**	Neat	477	0.18, 0.28	18.2	16.7	[27]
	**Cz-CzCN**	Neat	510	0.26, 0.52	17.1	14.5	[28]
	**TA-3Cz**	Neat	546	0.39, 0.56	11.8	11.2	[29]
	**PCzDP-10**	Neat	496	0.24, 0.40	16.1	N/A	[30]
	**PABPC25**	Neat	560	0.44, 0.54	16.1	15.9	[31]
	**PABPC10**	Neat	549	0.40, 0.56	16.2	16.0	
	**PABPC5**	Neat	549	0.40, 0.56	18.1	17.7	
	**PABPC1**	Neat	541	0.36, 0.57	15.4	14.2	
	**LEP**	Neat	N/A	0.32, 0.56	10	N/A	[32]
	**PPxPhO**	20 wt% in	550	N/A	11.8	N/A	[33]

		mCP					
	**R-P**	10 wt% in mCP	546	0.41, 0.57	14.9	12.7	[34]
	**S-P**		544	0.40, 0.57	15.8	12.3	
	**P1-05**	Neat	483	0.20, 0.37	11.3	N/A	[35]
	**pBP-PXZ**	Neat	584	0.52, 0.48	13.7	N/A	[36]
	**DMAC-BP-*t*BuCz**	30 wt% in mCPCN	520	0.28, 0.52	20.9	20.4	[37]
	**DMAC-BP-*t*Bu3Cz**		520	0.28, 0.52	28.0	24.9	
	**DMAC-BP-*t*Bu7Cz**		520	0.29, 0.52	25.6	17.6	
	**poly(AcBPCz-TMP)**	Neat	507	0.25,0.52	23.5	18.0	[38]
	**P1**	Neat	545	0.38, 0.55	15.7	5.0	[39]
	**P2**	Neat	543	0.38, 0.55	14.4	11.2	
	**P3**	Neat	553	0.44,0.54	21.9	20.1	
	**P4**	Neat	553	0.44,0.54	20.3	19.0	
	**P5**	Neat	553	0.42,0.55	25.4	24.2	
MR-TADF	**OAB-ABP-1**	98 wt% in polymer B	505	0.12, 0.63	21.8	17.4	[40]
	**PCzBN1**	In mCP	491	0.10, 0.43	17.8	N/A	[41]
	**PCzBN3**		496	0.12, 0.54	17.5	N/A	
	**BN-36Cz-BN**	5 wt% in DMIC-TRZ	495	0.11, 0.50	27.1	2.9	[42]
	**BN-27Cz-BN**		498	0.13, 0.57	20.9	3.3	
	**4FICzBN**	16 wt% in mCP	496	0.10, 0.50	12.2	11.0[a]	[43]
	**6TBN**	16 wt% in mCP	496	0.09, 0.52	23.0	6.4	[44]
		Neat	496	0.11, 0.51	12.3	1.7	
	**BNB'-1**	1.5 wt% in **PhCzBCz**	540	N/A	36.2	N/A	[45]
	**PCzTBN3**	Neat	510	0.17, 0.63	15.0	2.8	[46]
	**PCzTBN5**	Neat	511	0.18, 0.62	22.2	4.0	
	**4FlCzBN**	16 wt% in mCP	500	0.11,0.57	10.9	4.6	[47]
	**Cy-tmCPBN**	8 wt% in Cy-tmCP	496	0.13, 0.55	21.9	N/A	[48]
	**2GtBuCzCO_2HDCzB**	30 wt% in mCP	495	0.08, 0.53	27.9	22.3[a]	This work
		Neat	496	0.14, 0.54	24.0	20.2	
	**tBuCzCO_2HDCzB**	30 wt% in	496	0.09, 0.53	22.0	16.3[a]	

		mCP					
		Neat	498	0.15, 0.56	11.4	9.5	

[a] The EQE at 5000 cd/ m^2。

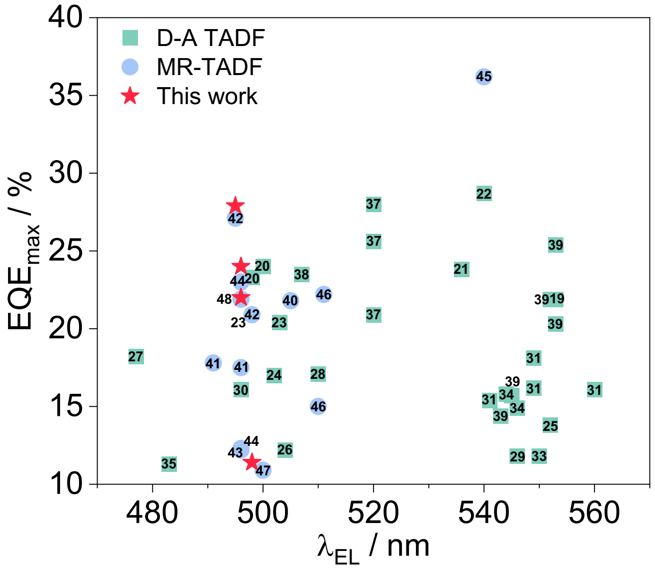

Figure **S52**. EQE$_{max}$ of reported green solution-processed OLEDs as a function of λ_{EL}. Only examples with EQE$_{max}$ > 10% are shown.

References

[1] M. J. Frisch, G. W. Trucks, H. B. Schlegel, G. E. Scuseria, M. A. Robb, J. R. Cheeseman, G. Scalmani, V. Barone, G. A. Petersson, H. Nakatsuji, X. Li, M. Caricato, A. V. Marenich, J. Bloino, B. G. Janesko, R. Gomperts, B. Mennucci, H. P. Hratchian, J. V. Ortiz, A. F. Izmaylov, J. L. Sonnenberg, Williams, F. Ding, F. Lipparini, F. Egidi, J. Goings, B. Peng, A. Petrone, T. Henderson, D. Ranasinghe, V. G. Zakrzewski, J. Gao, N. Rega, G. Zheng, W. Liang, M. Hada, M. Ehara, K. Toyota, R. Fukuda, J. Hasegawa, M. Ishida, T. Nakajima, Y. Honda, O. Kitao, H. Nakai, T. Vreven, K. Throssell, J. A. Montgomery Jr., J. E. Peralta, F. Ogliaro, M. J. Bearpark, J. J. Heyd, E. N. Brothers, K. N. Kudin, V. N. Staroverov, T. A. Keith, R. Kobayashi, J. Normand, K. Raghavachari, A. P. Rendell, J. C. Burant, S. S. Iyengar, J. Tomasi, M. Cossi, J. M. Millam, M. Klene, C. Adamo, R. Cammi, J. W. Ochterski, R. L. Martin, K. Morokuma, O. Farkas, J. B. Foresman, D. J. Fox, Wallingford, CT 2016

[2] R. Dennington, T. A. Keith, J. M. Millam, *Semichem Inc Shawnee Mission KS* 2019.

[3] N. O. C. Winter, C. Hättig, *J. Chem. Phys.* 2011, *134*, 184101.

[4] S. Hirata and M. Head-Gordon, *Chem. Phys. Lett.,* 1999, 314, 291–299.

[5] Y. Zhao, D. G. Truhlar, *Theor. Chem. Acc.* 2008, *120*, 215.

[6] T. H. Dunning Jr., *J. Chem. Phys.* 1989, *90*, 1007.

[7] E. Runge, E. K. U. Gross, *Phys. Rev. Lett.* 1984, *52*, 997.

[8] K. B. Wiberg, *J. Comput. Chem.* 2004, *25*, 1342.

[9] N. M. O'Boyle, A. L. Tenderholt, K. M. Langner, *J Comput. Chem.* 2008, *29*, 839.

[10] J. D. Hunter, *Computing in Science & Engineering* 2007, *9*, 90.

[11] M. M. McKerns, L. Strand, T. Sullivan, A. Fang, M. A. G. Aivazis, S. M. J. van der Walt, 2011.

[12] O. S. Lee, E. Zysman-Colman, Digichem (version 6) InSilico Computing, St Andrews, Scotland, 2024.

[13] N. G. Connelly, W. E. Geiger, *Chem. Rev.* 1996, *96*, 877.

[14] C. M. Cardona, W. Li, A. E. Kaifer, D. Stockdale, G. C. Bazan, *Adv. Mater.* 2011, *23*, 2367.

[15] G. A. Crosby, J. N. Demas, *J. Phys. Chem.* 1971, *75*, 991.

[16] W. H. Melhuish, *J. Phys. Chem.* 1961, *65*, 229.

[17] K. Masui, H. Nakanotani, C. Adachi, *Org. Electron.* 2013, *14*, 2721.

[18] Y. Tsuchiya, S. Diesing, F. Bencheikh, Y. Wada, P. L. dos Santos, H. Kaji, E. Zysman-Colman, I. D. W. Samuel, C. Adachi, *J. Phys. Chem. A* 2021, *125*, 8074.

[19] X. Wang, J. Hu, J. Lv, Q. Yang, H. Tian, S. Shao, L. Wang, X. Jing, F. Wang, *Angew. Chem., Int. Ed. Engl.* 2021, *60*, 16585.

[20] C. Li, A. K. Harrison, Y. Liu, Z. Zhao, C. Zeng, F. B. Dias, Z. Ren, S. Yan, M. R. Bryce, *Angew. Chem., Int. Ed. Engl.* 2022, *61*, 202115140.

[21] D. Sun, R. Saxena, X. Fan, S. Athanasopoulos, E. Duda, M. Zhang, S. Bagnich, X. Zhang, E. Zysman-Colman, A. Köhler, *Adv. Sci.* 2022, *9*, 202201470.

[22] D. Sun, E. Duda, X. Fan, R. Saxena, M. Zhang, S. Bagnich, X. Zhang, A. Köhler, E. Zysman-Colman, *Adv. Mater.* 2022, *34*, 202110344.

[23] C. Zhang, H. Yan, Y. He, Y. Chai, D. Zhou, *Mater. Chem. Front.* 2022, *6*, 3442.

[24] K. Matsuoka, K. Albrecht, A. Nakayama, K. Yamamoto, K. Fujita, *ACS Appl. Mater. Interfaces.* 2018, *10*, 33343.

[25] Y. Li, G. Xie, S. Gong, K. Wu, C. Yang, *Chem. Sci.* 2016, *7*, 5441.

[26] J. Luo, S. Gong, Y. Gu, T. Chen, Y. Li, C. Zhong, G. Xie, C. Yang, *J. Mater. Chem. C*, 2016, *4*, 2442.

[27] X. Wang, J. Hu, J. Lv, Q. Yang, H. Tian, S. Shao, L. Wang, X. Jing, F. Wang, *Angew. Chem., Int. Ed. Engl.* 2021, *60*, 16585.

[28] X. Ban, A. Zhu, T. Zhang, Z. Tong, W. Jiang, Y. Sun, *ACS Appl. Mater. Interfaces* 2017, *9*, 21900.

[29] K. Sun, Y. Sun, T. Huang, J. Luo, W. Jiang, Y. Sun, *Org. Electron.* 2017, *42*, 123.

[30] G. Xie, J. Luo, M. Huang, T. Chen, K. Wu, S. Gong, C. Yang, *Adv. Mater.* 2017, *29*, 201604223.

[31] Y. Yang, S. Wang, Y. Zhu, Y. Wang, H. Zhan, Y. Cheng, *Adv. Funct. Mater.* 2018, *28*, 201706916.

[32] A. E. Nikolaenko, M. Cass, F. Bourcet, D. Mohamad, M. Roberts, *Adv. Mater.* 2015, *27*, 7236.

[33] J. Zhang, Q. Wei, L. Lyu, L. Cao, M. Zhao, N. Fei, T. Wang, Z. Ge, *Macromol. Chem. Phys.* 2022, *223*, 202200023.

[34] J. M. Teng, D. W. Zhang, Y. F. Wang, C. F. Chen, *ACS Appl. Mater. Interfaces* 2022, *14*, 1578.

[35] J. Hu, Y. Chang, F. Chen, Q. Yang, S. Shao, L. Wang, *J. Polym. Sci.* 2022, *60*, 1855.

[36] Z. Zhao, Y. Liu, L. Hua, S. Yan, Z. Ren, *Adv. Funct. Mater* 2022, *32*, 202200018.

[37] K. Shi, Y. Xie, L. Hua, S. Li, Z. Yang, Y. Yin, Z. Wang, S. Ying, Y. Liu, Z. Ren, S. Yan, *ACS mater. lett.* 2024, *6*, 1491.

[38] J. Rao, X. Liu, X. Li, L. Yang, L. Zhao, S. Wang, J. Ding, L. Wang, *Angew. Chem., Int. Ed. Engl.* 2020, *59*, 1320.

[39] Y. Liu, Y. Xie, L. Hua, X. Tong, S. Ying, Z. Ren, S. Yan, *CCS Chemistry* 2023, *5*, 1005.

[40] N. Ikeda, S. Oda, R. Matsumoto, M. Yoshioka, D. Fukushima, K. Yoshiura, N. Yasuda, T. Hatakeyama, *Adv. Mater.* 2020, *32*, 202004072.

[41] T. Wang, Y. Zou, Z. Huang, N. Li, J. Miao, C. Yang, *Angew. Chem., Int. Ed. Engl.* 2022, *61*, 11172.

[42] T. Wang, X. Yin, X. Cao, C. Yang, *Angew. Chem., Int. Ed. Engl.* 2023, *62*, 01988.

[43] N. Peethani, N. Y. Kwon, C. W. Koh, S. H. Park, J. M. Ha, M. J. Cho, H. Y. Woo, S. Park, D. H. Choi, *Adv. Opt. Mater.* 2023, DOI 10.1002/adom.202301217.

[44] F. M. Xie, H. Z. Li, K. Zhang, H. Y. Wang, Y. Q. Li, J. X. Tang, *ACS Appl. Mater. Interfaces* 2023, *15*, 39669.

[45] H. Wang, X. C. Fan, J. X. Chen, Y. C. Cheng, X. Zhang, H. Wu, X. Xiong, J. Yu, K. Wang, X. H. Zhang, *Adv. Funct. Mater.* 2023, *33*, 202306394.

[46] T. Wang, Z. Huang, H. Zhang, J. Miao, C. Yang, *Adv. Funct. Mater* 2024, 202408119.

[47] N. Peethani, N. Y. Kwon, C. W. Koh, S. H. Park, J. M. Ha, M. J. Cho, H. Y. Woo, S. Park, D. H. Choi, *Adv. Opt. Mater.* 2024, *12*, 202301217.

[48] N. Y. Kwon, H. Kwak, H. Y. Kim, S. H. Park, J. Y. Park, M. J. Kang, C. W. Koh, S. Park, M. J. Cho, D. H. Choi, *Chem. Sci.* 2024, *15*, 12361.
